# A DNA barcode reference library for the Tipulidae (Insecta, Diptera) of Germany

**DOI:** 10.3897/BDJ.12.e127190

**Published:** 2024-09-24

**Authors:** Moritz Fahldieck, Björn Rulik, Jana Thormann, Ximo Mengual

**Affiliations:** 1 Museum Koenig, Leibniz-Institut zur Analyse des Biodiversitätswandels, Bonn, Germany Museum Koenig, Leibniz-Institut zur Analyse des Biodiversitätswandels Bonn Germany

**Keywords:** Tipulidae, DNA barcoding, crane flies, species identification, biodiversity, biomonitoring, taxonomy, COI barcodes, German Barcode of Life initiative (GBOL), species delimitation algorithm

## Abstract

Tipulidae, commonly known as true crane flies, represent one of the most species-rich dipteran families, boasting approximately 4,500 known species globally. Their larvae serve as vital decomposers across diverse ecosystems, prompting their frequent and close observation in biomonitoring programs. However, traditional morphological identification methods are laborious and time-consuming, underscoring the need for a comprehensive DNA barcode reference library to speed up species determination.

In this study, we present the outcomes of the German Barcode of Life initiative focused on Tipulidae. Our DNA barcode library comprises 824 high-quality cytochrome c oxidase I (COI) barcodes encompassing 76 crane fly species, counting for ca. 54% of the German tipulid fauna. Our results significantly increased the number of European tipulid species available in the Barcode of Life Data System (BOLD) by 14%. Additionally, the number of barcodes from European tipulid specimens more than doubled, with an increase of 118%, bolstering the DNA resource for future identification inquiries.

Employing diverse species delimitation algorithms — including the multi-rate Poisson tree processes model (mPTP), Barcode Index Number assignments (BIN), Assemble Species by Automatic Partitioning (ASAP), and the TaxCI R-script — we successfully match 76-86% of the morphologically identified species. Further validation through neighbor-joining tree topology analysis and comparison with 712 additional European tipulid barcodes yield a remarkable 89% success rate for the species identification of German tipulids based on COI barcodes.

This comprehensive DNA barcode dataset not only enhances species identification accuracy but also serves as a pivotal resource for ecological and biomonitoring studies, fostering a deeper understanding of crane fly diversity and distribution across terrestrial landscapes.

## Introduction

The superfamily Tipuloidea (Insecta, Diptera), commonly known as crane flies, comprises four families, namely Tipulidae, Pediciidae, Cylindrotomidae, and Limoniidae (Oosterbroek 2023). However, it is worth noting that Limoniidae appears to represent a paraphyletic grouping of all crane fly genera that do not fit into one of the other families (Petersen et al. 2010, Zhang et al. 2016; but see Starý (2021)). The sister group of crane flies is Trichoceridae, whose members are known as winter gnats, and together they form the Tipulomorpha clade.

The best-known crane flies are the tipulids, which constitute a very large family comprising approximately 4,500 known species worldwide (Oosterbroek 2023). Within Europe, there are 519 recorded species, with 142 taxa present in Germany, grouped into nine genera: *Ctenophora* Meigen, 1803 (7 species), *Dictenidia* Brulle, 1833 (1 species), *Dolichopeza* Curtis, 1825 (2 species), *Nephrotoma* Meigen, 1803 (20 species), *Nigrotipula* Hutson and Vane-Wright, 1969 (1 species), *Phoroctenia* Coquillett, 1910 (1 species), *Prionocera* Loew, 1844 (4 species), *Tanyptera* Latreille, 1804 (2 species), and *Tipula* Linnaeus, 1758 (104 species). The substantial number of species within the genus *Tipula* is a result of the work done by Charles P. Alexander (1889 – 1981), who used subgenera to further classify various clades within this family. In Germany, the genus *Tipula* is represented by members of 14 subgenera (Oosterbroek 2023).

**Figure 1.**
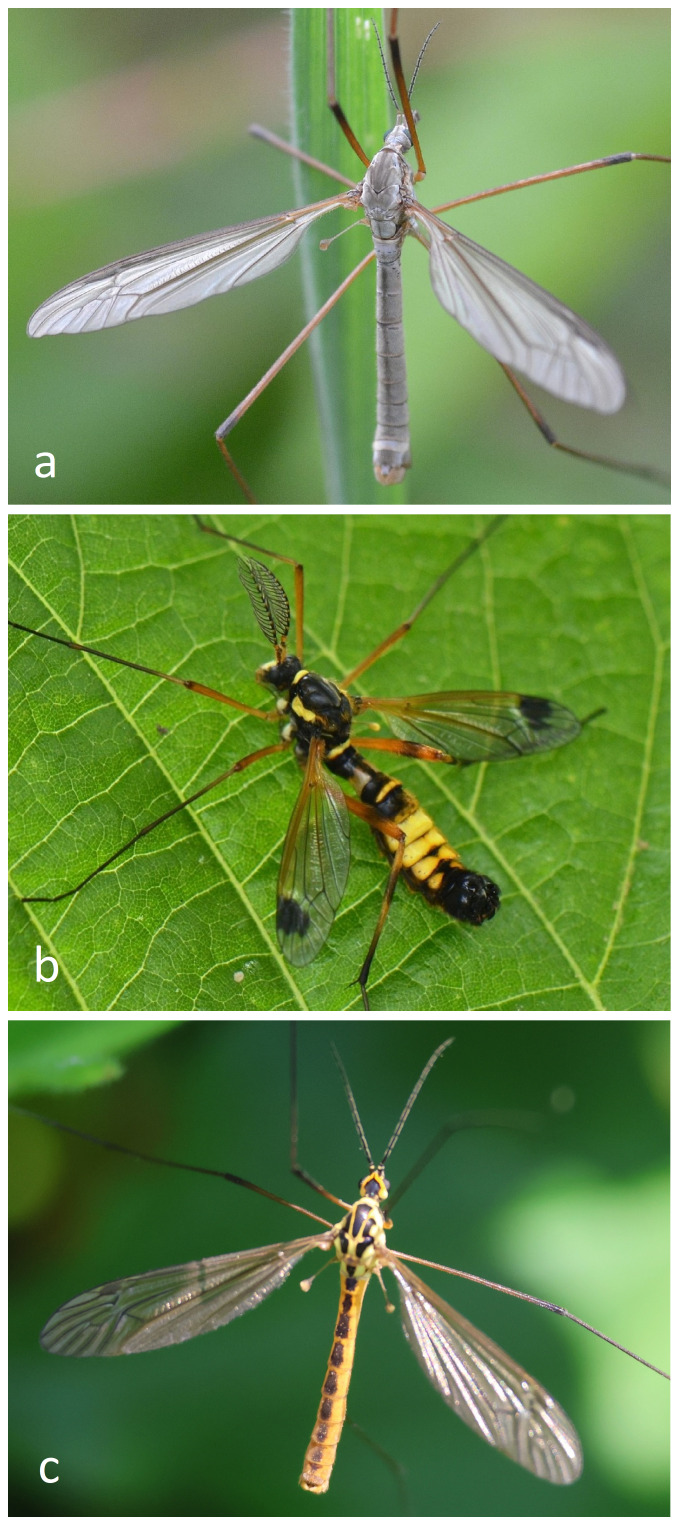
Examples for the three main morphotypes of German Tipulidae. (a) Tipula (Tipula) paludosa Meigen, 1830, ♂, © Chris walker (CC BY 4.0); (b) Ctenophora (Cnemoncosis) festiva Meigen, 1804, ♂, © Bartholomeus van der Geer (CC BY 4.0); and (c) *Nephrotomaflavescens* (Linnaeus, 1758), ♂, © jerry2018 (CC BY 4.0); all photos taken from iNaturalist, https://www.inaturalist.org, accessed 08.05.2024.

Tipulidae are nematoceran flies with elongated bodies, long wings, thread-like antennae, and very long legs. Like most nematoceran flies, one of the most reliable characteristics for identifying them is wing venation, which is quite uniform and therefore family-specific for the crane flies. Tipulidae species are often the largest among crane flies, making them well-known even to non-entomologists. Species of the genus *Tipula* are typically large and exhibit a dull and relatively uniform coloration, ranging from brown to grey or yellowish (Fig. 1a). However, some species within this genus may have distinct color markings (usually black, but also brown, grey or white) on the wing (Stubbs 2021). In contrast, species of the genera *Ctenophora* and *Tanyptera* can display striking wing and body colors, featuring black and red or black and yellow body patterns. They are usually large with prominent dark patches on their wings (Fig. 1b). Males of these genera also possess long, feathery antennae (Stubbs 2021). They mimic ichneumonids and wasps (Insecta: Hymenoptera) in their overall appearance (Stubbs 2021). A third primary tipulid morphotype can be observed in the genus *Nephrotoma*, where specimens are typically slightly smaller than those in the previous groups and exhibit a characteristic patchy body pattern of black and yellow (Fig. 1c). The wings of these species usually lack any distinctive patterns, except for a possible black stigma (Stubbs 2021). Fig. 1 provides an overview about the three primary morphotypes.

The larvae of Tipulidae develop in soil that typically contains at least some degree of moisture or may even be fully water-saturated, such as along the shores of lakes or riverbanks. In Tipulidae, the vast majority of larvae feed on decaying plant material, including leaf litter, humus, or decaying wood (as seen for *Ctenophora*). Some larvae can also feed on higher plants, like Tipula (Tipula) paludosa Meigen, 1830, which feeds on the roots and leaves of grass and can, under certain circumstances, be considered a pest (Stubbs 2021). Aside from these rare cases, Tipulidae play an important role in the ecosystems where they are found. They are significant organic matter decomposers due to their activity in the soil, and because of their large size and high biomass, they serve as a crucial food source for birds, bats, and other small mammals, as well as for predatory insects such as dragonflies, assassin flies, robber flies, social wasps, among others (Stubbs 2021). In contrast to their larval stage, adults have a short lifespan. They typically only survive for a few days after emerging from the pupa. During this brief adult life, they usually do not feed but rather seek a mate for reproduction. Therefore, adults tend to appear in small, species-specific and climate-sensitive intervals throughout the year (Devlin et al. 2022), except during the winter months.

Due to their high species and specimen numbers across a wide range of ecosystems, crane flies are frequently targeted in biomonitoring programs, either intentionally or incidentally as bycatch (e.g. Wagner et al. (2011), Geiger et al. (2016a)). Furthermore, they are included in national taxa lists within the states of the European Union, used for assessing water bodies as part of the implementation of the European Union's Water Framework Directive 2000/60/EC (see e.g. Mauch et al. (2003) for Germany). Morphological identification of all observed species and specimens from species-rich taxa like Tipulidae would significantly enhance the informativeness of such biomonitoring approaches. However, the analysis of the samples from most traditional biomonitoring methods is exceedingly time-consuming, and experts are either lacking or at capacity with other responsibilities. It is crucial to extend the existing approaches and boost their determination speed and informative output to tackle the massive losses in biodiversity that are recognized not just in Germany but worldwide (Kolbert 2014, Hallmann et al. 2017, Cowie et al. 2022).

Therefore, the International Barcode of Life initiative (iBOL) was formed in the first decade of the 21^st^ century with the aim of addressing the gap between the enormous quantities of material and data that need to be analyzed and the lack of expertise. The initiative seeks to establish DNA barcoding as a standard, cost- and time-efficient approach for the analysis of biomonitoring study results (Hebert et al. 2003). In the case of animals, the 5’ end of the cytochrome c oxidase subunit I (COI) gene is used as DNA barcode (Hebert et al. 2003). In 2007, the Barcode of Life Data System (BOLD) was created as a central workbench and database for both already produced and future DNA barcodes (Ratnasingham and Hebert 2007). The German Barcode of Life project (GBOL), as part of the iBOL initiative, began in 2012 with the goal of collecting and barcoding all species of German animals, plants, and fungi while making the data publicly available (Geiger et al. 2016a, Geiger et al. 2016b). This initial approach to building a barcode database is crucial for accurate and effective biomonitoring because species identification through DNA barcoding or bulk sample methods like metabarcoding relies on correctly identified, well-documented, and vouchered specimens. GBOL is currently in its third phase (GBOL III: Dark Taxa) and focuses on the so-called “dark taxa”. The term “dark taxa” was first created by Roderic D. M. Page in 2011. It refers to species listed in genetic databases, such as GenBank or BOLD, without formal scientific names. These taxa are typically identified by genus names and alphanumeric codes or identifiers, hindering precise taxonomic classification and potentially representing both undiscovered and previously described but unlinked species (Page 2011, Page 2016). In this sense GBOL is focusing on the megadiverse orders Diptera and Hymenoptera, which constitute a significant portion of the biodiversity in environmental and bulk samples but remain severely understudied (Hausmann et al. 2020). Despite their large size, beauty, or morphological species-level identifiability, some Tipulidae species can still be considered a “dark taxon” in terms of the number of unlinked specimens represented in BOLD.

Since the start of the GBOL project, several important and valuable studies have been published, presenting and testing built-up DNA barcode databases for other arthropod groups in Germany, such as Heteroptera (Raupach et al. 2014, Havemann et al. 2018), Neuropterida (Morinière et al. 2014), Coleoptera (Hendrich et al. 2014, Raupach et al. 2016, Rulik et al. 2017, Raupach et al. 2020), Arachnida (Ahrens et al. 2016, Muster et al. 2021), Orthoptera (Hawlitschek et al. 2016), Ephemeroptera, Plecoptera, and Trichoptera (Morinière et al. 2017), some Hymenoptera groups (Schmid‐Egger et al. 2018), or Odonata (Geiger et al. 2021). Regarding the two-winged insect order, a DNA barcode library for 5,200 German dipterans was published by Morinière et al. (2019), including 46 Tipulidae Barcode Index Numbers (BINs).

Beyond GBOL, numerous other initiatives and approaches focusing on Diptera or crane flies have also emerged globally. For instance, Meier and Zhang (2009) evaluated the effectiveness of DNA barcoding for species identification and taxonomy in Diptera, using 4,261 COI sequences from 1,001 species. Similarly, the InBIO Barcoding Initiative (IBI) produced a dataset of 412 specimens of crane flies (Limoniidae, Pediciidae, and Tipulidae) from Portugal, representing 83 species — 55% of all known crane fly species in the country (Ferreira et al. 2021).

Our aim with this study is to present the most comprehensive library of high-quality DNA barcodes for German Tipulidae, along with detailed quality checks for each barcode and species hypothesis based on morphological species identification of all specimens, neighbor-joining tree topology analysis, four different species delimitation algorithms, and a comparison with other already published Tipulidae DNA barcodes on BOLD.

## Material and Methods

### Specimens and taxonomy

This study includes all the COI barcode sequences of Tipulidae specimens that have been sequenced as part of the German Barcode of Life (GBOL) project and are currently housed at the Leibniz Institute for the Analysis of Biodiversity Change/Museum Koenig (LIB/ZFMK) in Bonn, Germany.

Prior to sequencing, specimens were morphologically identified either by associated taxon experts or by entomologists working in the project. The majority of the included specimens were initially identified or re-identified by the first author using the identification keys from Stubbs (2021), in combination with a comparison of pictures and data provided by Oosterbroek (2023) and other specimens from the GBOL voucher specimens collection at the LIB/ZFMK. Some morphological species identifications also rely on original descriptions or additional taxonomic publications. In a very few cases, specimens were identified in reverse after sequencing, combining morphological identifications with sequence cluster analyses. Some females, which were not morphologically identifiable with certainty, were identified through cluster analysis after their barcodes clustered together with correctly identified males. Additionally, a single larval specimen was also identified using this approach.

### Laboratory protocols

Wet lab work was primarily conducted in the laboratory of the LIB/ZFMK. A small portion of the samples was part of the Global Malaise Trap program and processed at the Canadian Centre for DNA Barcoding (CCDB) in Guelph, Canada. At the LIB/ZFMK, DNA was typically isolated from one leg and muscle tissue from the coxa using a Qiagen (Hilden, Germany) BioSprint96 magnetic bead extractor and corresponding kits. Polymerase chain reactions (PCR) for the 5’ segment of the mitochondrial cytochrome c oxidase subunit 1 (COI) gene were carried out in total reaction volumes of 20 μl. These included 2 μl of undiluted DNA template, 0.8 μl of each primer (10 pmol/μl), and standard amounts of the reagents provided with the Multiplex PCR kit from Qiagen. LCO1490-JJ [5’-CHACWAAYCATAAAGATATYGG-3’] and HCO2198-JJ [5’-AWACTTCVGGRTGVCCAAARAATCA-3’] designed by Astrin and Stüben (2008) were used as standard primers. Thermal cycling was performed using Applied Biosystems 2720 Thermal Cyclers (Life Technologies, Carlsbad, CA, USA) with a PCR program comprising two cycle sets, combining a “touchdown” and a “step-up” routine. The first cycle set (15 repeats) involved 35 seconds of denaturation at 94 °C, 90 seconds of annealing at 55°C (decreasing by 1 °C per cycle), and 90 seconds of extension at 72 °C. The second cycle set (25 repeats) included 35 seconds of denaturation at 94 °C, 90 seconds of annealing at 45 °C, and 90 seconds of extension at 72 °C. Unpurified PCR products were subsequently sent for bidirectional Sanger sequencing to BGI (Hong Kong, China) or, in some cases, to Macrogen (Amsterdam, Netherlands).

### Data analysis and species delimitation algorithms

The obtained sequences were checked for the occurrence of stop-codons or hints of nuclear mitochondrial DNA segments (NUMTs) and validated using Geneious R7 and Geneious Prime (Biomatters Ltd.) before being linked to their respective entries in the GBOL database through the Diversity Collection module in Diversity Workbench. The complete dataset, including all COI barcode sequences, identifications, and metadata, was uploaded to BOLD (Dataset: DS-GDIPTIP; https://doi.org/10.5883/DS-GDIPTIP) and subsequently submitted to GenBank (ON843162-ON842360, OR178275-OR178255).

Intra- and interspecific uncorrected distances (p-distances) for the dataset as well as pairwise p-distances were calculated using the DiStats Perl-script (Astrin et al. 2016) available on GitHub (https://github.com/mptrsen/distats, accessed 28.09.2023). The sequences were aligned with the MUSCLE alignment implementation in Geneious R7. For barcoding gap analysis, the “Barcode Gap Analysis” tool implemented in BOLD was used with the distance model option “Pairwise Distances” and ambiguous base/gap handling option “Pairwise Deletion”.

To better understand the intra- and interspecific p-distances in our dataset, we calculated the mean and mode of these two values. The mean was directly calculated using the DiStats Perl-script (Astrin et al. 2016). For the mode, we grouped different intraspecific distance values into ranges of 0.10%; in other words, we categorized values between 0.0% and 0.09%, between 0.10% and 0.19%, and so on. For interspecific p-distance values, we used a different grouping: instead of 0.10%, we used 1.0%. Thus, we grouped values between 0.0% and 0.99%, between 1.0% and 1.99%, and so forth to calculate the mode.

DNA-based species delimitation was applied to the sequences in the dataset using multiple methods, including the multi-rate Poisson tree processes (mPTP) model (Kapli et al. 2017), Barcode Index Number (BIN) assignments (Ratnasingham and Hebert 2013), the Assemble Species by Automatic Partitioning (ASAP) method (Puillandre et al. 2020), and the TaxCI R-script (Rulik et al. 2017).

The mPTP model (Kapli et al. 2017) is an improved version of PTP (Zhang et al. 2013) that is faster, does not require a similarity threshold as input, and uses Markov Chain Monte Carlo sampling for comprehensive delimitation evaluation. mPTP analysis was conducted using the mPTP webserver (https://mptp.h-its.org/#/tree, accessed on 28.09.2023). It utilizes a neighbor-joining tree in Newick format, incorporating a defined outgroup, p-distances, and pairwise deletion. The tree was created with MEGA11 based on a MUSCLE alignment in FASTA format obtained from Geneious R7. The used tree file included all obtained COI barcode sequences, along with an additional COI barcode of a Limoniidae: *Limonianubeculosa*, Meigen 1804 specimen (ZFMK-TIS-2010991) as outgroup.

The ASAP method (Puillandre et al. 2020) was implemented using the ASAP webserver (https://bioinfo.mnhn.fr/abi/public/asap, accessed on 28.09.2023). It utilizes the FASTA format MUSCLE alignment of all obtained COI sequences created with Geneious R7, with p-distances used as the setting. ASAP partitions species based on pairwise genetic distances, providing 10 different partitioning results ranked by a score. The best result, indicated by the lowest score, was selected for this study.

The BIN Discordance tool in BOLD uses the methodology developed by Ratnasingham and Hebert (2013). They introduced the Barcode Index Number (BIN) system to categorize and manage DNA barcode sequences for all animal species. This approach enables the differentiation of distinct BINs for different putative species, facilitating more precise taxonomic delineation and aiding in the organization and analysis of genetic data on a large scale. The BIN Discordance tool in BOLD was utilized with the dataset to identify molecular operational taxonomic units (MOTUs) based on BIN assignments.

Finally, the TaxCI R-script (Rulik et al. 2017) was used to generate a graphical tree with cluster analysis based on prior morphological species identifications. This script uses the MUSCLE alignment in PHYLIP format created with Geneious R7, a neighbor-joining tree produced using MEGA 11 with p-distances and pairwise deletion, in Newick format based on the alignment, and a data sheet containing all relevant specimen taxonomic information and metadata in CSV format, created using Microsoft® Excel 14.

For the sequences of the dataset, the accuracy of the species delimitation methods with prior morphological species identifications was assessed by the match ratio (Ahrens et al. 2016): 2 × Nmatch / (Nmol + Nmorph), where Nmatch is the number of exact matches of morphospecies (all individuals) with MOTUs of different delimitation methods, Nmol is the number of MOTUs that resulted from different delimitation methods, and Nmorph is the number of morphospecies.

As an additional attempt to further scrutinize the dataset, BOLD (https://www.boldsystems.org, accessed on 28.09.2023) was searched for all European tipulid COI barcodes with a length of at least 550 bp and with a species identification of the vouchered specimen. Sequences flagged by BOLD or containing stop codons were excluded. These already published barcodes are used to test the species hypotheses of the specimens in this study. Therefore, a second graphical tree including the GBOL Tipulidae sequences together with the additional European Tipulidae barcodes was created with the TaxCI script. The BOLD dataset DS-EUDIPTIP (https://doi.org/10.5883/DS-EUDIPTIP) comprises all utilized sequences, including flagged ones and those containing stop codons.

For both datasets, one containing only the GBOL Tipulidae sequences and the other incorporating both the GBOL Tipulidae sequences and additional European Tipulidae barcodes, we generated new graphical trees using the TaxCI script. These trees had the same topologies but different tree tip categories for analysis. Instead of analyzing the species names assigned to the sequences as suggested by Rulik et al. (2017), we examined the BINs assigned to them to identify potential misidentifications. This novel approach allowed for the marking of potential misidentifications using the color code of the TaxCI script (Rulik et al. 2017).

Subsequently, a final tree analyzing the species names and incorporating the GBOL Tipulidae and the additional European Tipulidae specimens while excluding clearly misidentified samples, was created using the TaxCI script.

## Results

### DNA barcoding

**Figure 2.**
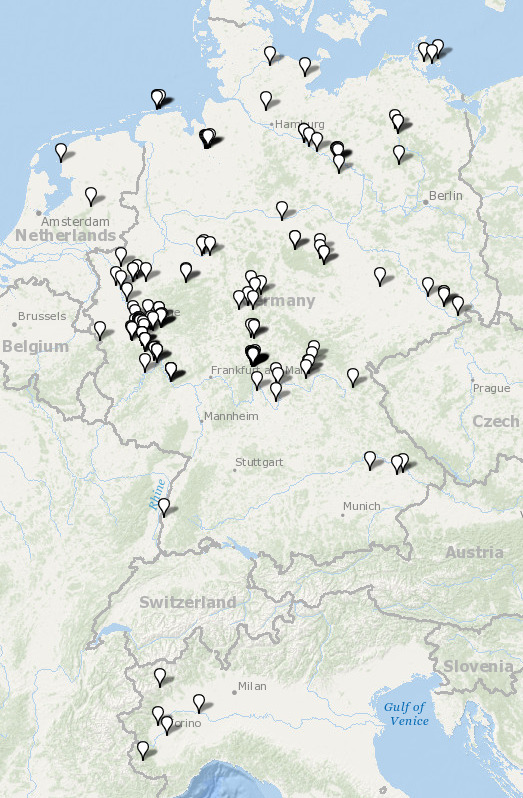
Collection sites of the 824 specimens of the GBOL Tipulidae.

**Figure 3.**
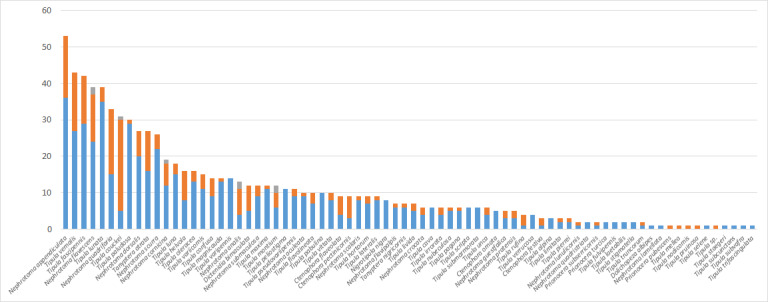
Number of analyzed specimens per species of the GBOL Tipulidae, sorted from most to least. Male specimens are represented in blue, female specimens in orange, and specimens with unidentifiable sex in grey.

**Figure 4.**
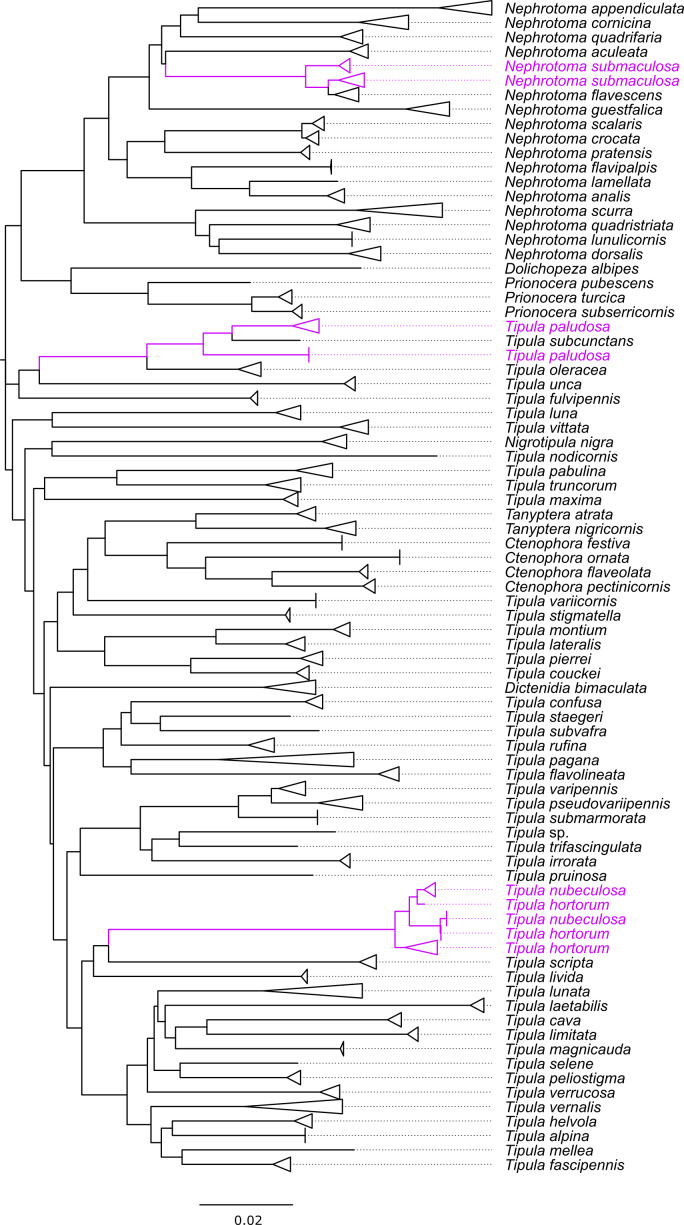
Neighbor-joining tree of the 76 species of the GBOL Tipulidae based on morphological identification. Species with more than one specimen are compressed. Pink species names indicate more than one cluster of the same species. Inter- and intraspecific p-distances are to scale.

We successfully sequenced the DNA barcode region of 824 tipulid specimens, representing 76 out of the 142 recorded species from Germany (54%). The fragment lengths of the analyzed DNA barcodes ranged from 555 to 658 bp, with only 34 barcodes (4%) being shorter than 658 bp. Additionally, the average base composition of the sequences was: A = 28.6%, C = 16.8%, G = 16.8%, and T = 37.8%. Tables S1.1 to S1.6 (Suppl. material 1) display the distribution of sex and life stage among the specimens, the country of origin, collection year, collection method, and storage method. Additionally, Fig. 2 illustrates the geographical locations where the specimens were sampled. The number of analyzed specimens per species varies from one (considered as singletons in 12 species) to 53 in *Nephrotomaappendiculata* (Pierre, 1919), as shown in Fig. 3. Furthermore, Fig. 4 depicts a neighbor-joining tree of the morphologically identified species, while Figure S1 (Suppl. material 2) illustrates the TaxCI tree for all 824 specimens of the GBOL Tipulidae.

The inclusion of GBOL Tipulidae specimen barcodes expands BOLD by 813 specimens and 53 species for Germany, and by 824 specimens and 14 species for Europe. In terms of specimen numbers for Germany, there is an increase of more than six fold (from 122 specimens before, an increase of 666%), while in terms of species for Germany, we now almost triple the previous number (from 30 species before, an increase of 177%). Concerning specimen numbers for Europe, we also more than double the previous number (from 698 specimens before, an increase of 118%), and in terms of species for Europe, we increase the previous number by 10% (140 species before). All numbers and percentages are based on barcodes with a minimum length of 550 bp. Notably, the 14 species new to BOLD for Europe represent first-time records, not just at a European level, but also globally. These species are Ctenophora (Cnemoncosis) festiva Meigen, 1804, Ctenophora (Ctenophora) flaveolata (Fabricius, 1794), *Nephrotomalamellata* (Riedel, 1910), Tipula (Lunatipula) alpina Loew, 1873, Tipula (Lunatipula) laetabilis Zetterstedt, 1838, Tipula (Lunatipula) livida van der Wulp, 1859, Tipula (Lunatipula) mellea Schummel, 1833, Tipula (Lunatipula) selene Meigen 1830, Tipula (Lunatipula) verrucosa Pierre, 1919, Tipula (Mediotipula) stigmatella Schummel, 1833, Tipula (Pterelachisus) pabulina Meigen, 1818, Tipula (Pterelachisus) sp., Tipula (Pterelachisus) trifascingulata Theowald, 1980, and Tipula (Savtshenkia) subvafra Lackschewitz, 1936.

Exploring the Barcode of Life Data System (BOLD, https://www.boldsystems.org, accessed on 01.08.2022) for previously-published COI barcode sequences of identified Tipulidae from Europe, with a minimum length of 550 bp, resulted in 700 specimen barcodes. However, four specimens were excluded from further analyses: one (PII-20110076) contained a stop codon, while three others were flagged by BOLD. The flagged specimens ABOL-BioBlitz 2019 Szucsich-0234 and bf-dip-00503 were suspected of possible contamination or misidentification, and PII-20110081 was marked due to a putative reading frame shift issue. Consequently, the dataset from BOLD comprised 696 specimen barcodes from 141 species.

Tables S2.1 to S2.3 (Suppl. material 3) present the distribution of sex, life stages, and collection countries for these specimens. Figure S2 (Suppl. material 4) showcases a TaxCI graphical analysis tree containing all GBOL Tipulidae specimens alongside these 696 European specimen barcodes, totaling 1520 DNA barcodes.

Two TaxCI trees scrutinizing the BINs of the barcodes are presented as Figure S3 (Suppl. material 5) for GBOL Tipulidae and as Figure S4 (Suppl. material 6) for both GBOL Tipulidae and the European specimens from BOLD. These analyses highlighted eight misidentifications as interpreted by us, exclusively among the European specimens from BOLD (BC-ZSM-DIP-24041-E05, bf-dip-00132, bf-dip-00140, JES-20120002, JES-20120110, NI_Tip_1, NMS-10003395, and NMS-10004058). A subsequent TaxCI tree, Figure S5 (Suppl. material 7), was constructed without these eight sequences.

### Data analysis and species delimitation algorithms

Tables S3 and S4 (Suppl. materials 8, 9) display the outcomes of the DiStats calculations (Astrin et al. 2016). Table S3 (Suppl. material 8) comprises the intra- and interspecific p-distances among the 76 species, while Table S4 (Suppl. material 9) has the pairwise p-distances among all 824 specimens.

**Figure 5.**
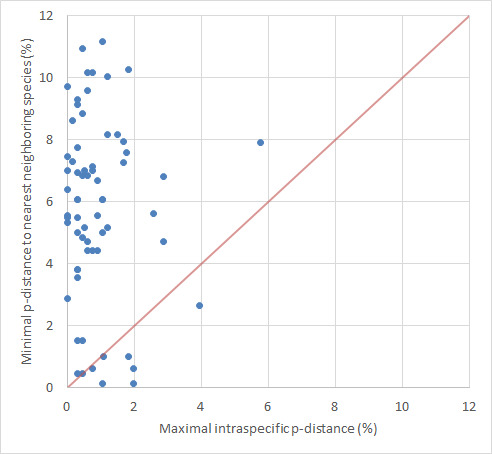
Maximal intra-specific p-distances plotted against the minimal p-distances to the nearest neighboring species for the GBOL Tipulidae.

Although a barcoding gap analysis was conducted, no distinct, generalized barcoding gap was identified. Nevertheless, most species exhibit a barcoding gap, shown by significantly higher intraspecific genetic distance compared to interspecific distances (see Fig. 5). Notably, the minimum intraspecific p-distance was 0% (observed in 54 non-singleton species), and the maximum intraspecific p-distance recorded was 5.78% (in T. (S.) pagana Meigen, 1818). The mean intraspecific p-distance was 0.35%. The mode of the mean intraspecific p-distances falls within the range of 0.10% to 0.19%, with the most frequent value occurring in 14 out of 64 cases.

Regarding distances to the nearest neighboring species, the minimum interspecific p-distance recorded was 0.15% (between T. (V.) hortorum Linnaeus, 1758 and T. (V.) nubeculosa Meigen, 1804), while the maximum was 12.92% (between T. (Odonatisca) nodicornis Meigen, 1818 and T. (P.) trifascingulata). The mean p-distance to the nearest neighboring species stood at 5.94%. The mode of the p-distances to the nearest neighboring species falls within the range of 5.00% to 5.99%, with the most frequent value occurring in 14 out of 76 cases.

**Figure 6.**
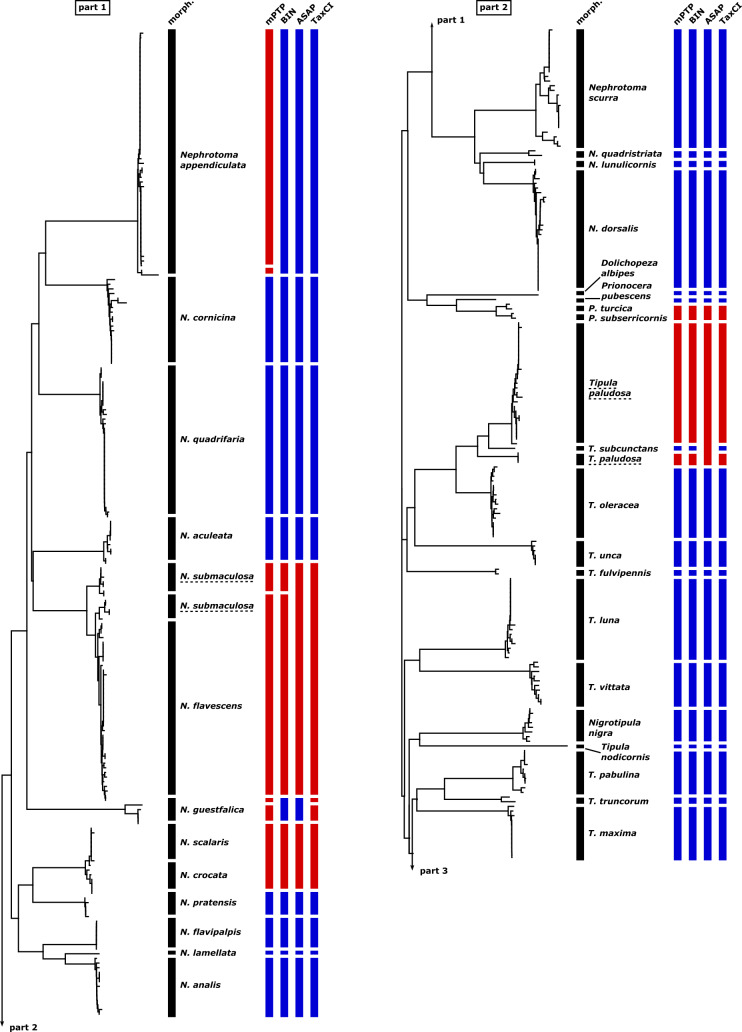
Neighbor-joining tree of the 76 species of the GBOL Tipulidae based on morphological identification, contrasted with the outcomes from the species delimitation methods mPTP, BIN, ASAP, and TaxCI applied. Dashed underlined species names indicate more than one cluster of the same species. Blue boxes indicate agreement between molecular species delimitation method and morphological species identification while red boxes indicate disagreement.

**Figure 7.**
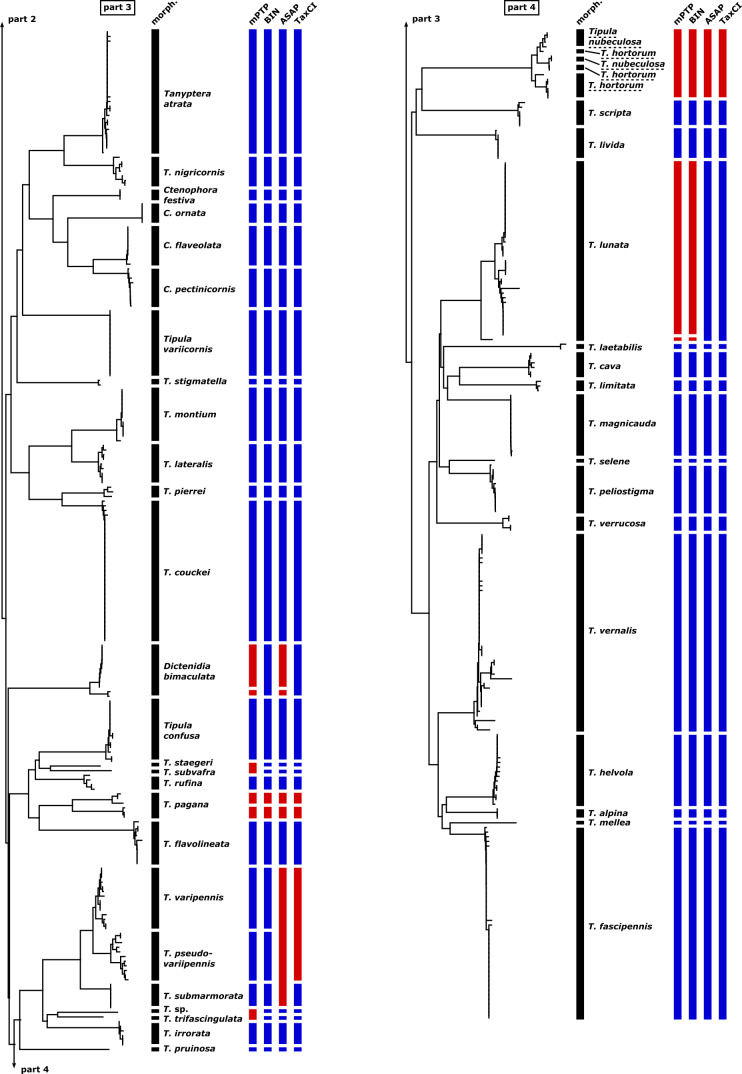
Continued.

In Table 1, the match ratios (Ahrens et al. 2016) derived from the four different DNA-based species delimitation methods (mPTP, BIN, ASAP, and TaxCI) applied to the sequences are presented. Overall, there is a success rate of 76-86% between the morphologically identified species and the four species delimitation algorithms, with the BIN algorithm having the highest rate (86%) and mPTP the lowest (76%). Figs 6, 7 offer a comprehensive visualization of our results, showcasing the neighbor-joining tree based on morphological species identification, contrasted with the outcomes from the four species identification algorithms applied.

### Species clusters

Four species pairs are recognized as the same species by at least one of the four species delimitation algorithms, i.e., mPTP, BIN, ASAP, and TaxCI. These pairs also exhibit very low interspecific p-distances (0.15-2.43; see Table S3: Suppl. material 8) and are not easily distinguishable solely based on their COI barcodes: *N.submaculosa* Edwards, 1928 and *N.flavescens* (Linnaeus, 1758); *N.crocata* (Linnaeus, 1758) and *N.scalaris* (Meigen, 1818); *Prionocerasubserricornis* (Zetterstedt, 1851) and *P.turcica* (Fabricius, 1787); and T. (V.) hortorum and T. (V.) nubeculosa. In the first three mentioned species pairs – *N.submaculosa* and *N.flavescens*, *N.crocata* and *N.scalaris*, as well as *P.subserricornis* and *P.turcica* – the barcodes of GBOL specimens are well separated by species but become mixed when we add additional barcodes from other European tipulid specimens. In contrast, the cluster of T. (V.) hortorum and T. (V.) nubeculosa barcodes is the only one in the neighbor-joining tree where the barcodes of the two different species (identified using morphology) do not form distinct clusters but instead create a unique mixed cluster from the outset.

Two other species pairs, with high interspecific p-distances (1.02-3.19 and 6.23; see Table S3: Suppl. material 8) are also recognized as the same species by at least one of the four species delimitation algorithms. In contrast to the four former mentioned species pairs, they remain well-separated even with additional barcode sequences from other European tipulid specimens added to the GBOL dataset. These species pairs are T. (P.) varipennis Meigen, 1818 and T. (P.) pseudovariipennis Czizek, 1912; and T. (S.) staegeri Nielsen, 1922 and T. (S.) subvafra.

Three different species are resolved into two unique barcode clusters each: T. (Tipula) paludosa, T. (S.) pagana Meigen, 1818, and the already challenging to identify *N.submaculosa*. The two clusters of T. (T.) paludosa are not neighboring in the graphical tree, displaying a relatively high p-distance between them (minimal inter-cluster p-distance = 3.34%). For T. (S.) pagana, the two neighboring clusters exhibit an even higher p-distance (minimal inter-cluster p-distance = 5.47%). The two clusters of *N.submaculosa* are not adjacent in the graphical representation and show a low p-distance between them (minimal inter-cluster p-distance = 1.52%). We were unable to find diagnostic morphological characteristics to distinguish these clusters.

Moreover, we have two species clusters featuring single DNA barcodes with relatively high p-distances (1.67 and 1.37; see Table S4: Suppl. material 9) with respect to their nearest neighbor from the same cluster: *N.guestfalica* (Westhoff, 1879) and T. (L.) lunata Linnaeus, 1758. In both species, two of the four species delimitation algorithms identify these outlier barcodes as an additional distinct species. We did not find morphological differences in these specimens.

Additionally, one female specimen of *T.* (*Pterelachisus)* could not be identified to species level. We retained it in the dataset as its barcode did not cluster with any other barcode from the GBOL Tipulidae or with barcodes from other European tipulid specimens in BOLD. For more detailed comments on all species clusters, see Appendix 1 (Suppl. material 10).

#### Nephrotomasubmaculosa Edwards, 1928 and Nephrotomaflavescens (Linnaeus, 1758)

The combined cluster of *N.submaculosa* and *N.flavescens* presents a complex topology (see Figure S1: Suppl. material 2). The cluster primarily comprises *N.submaculosa* and *N.flavescens*, with an additional “intermediate” form showing morphological resemblance to *N.submaculosa* but demonstrating COI barcodes more akin to *N.flavescens*. All four species delimitation algorithms distinguish the whole cluster as either a single species or occasionally two species, but never as three distinct species (see Figs 6, 7).

Upon the inclusion of more European tipulid barcodes, the pattern changes (see Figure S2: Suppl. material 4). A cluster including four Portuguese specimens of *N.submaculosa* emerges within the combined cluster of *N.submaculosa* and *N.flavescens*. Furthermore, a single barcode from a specimen of *N.flavescens* from Finland neighbors the combined cluster, while a separate cluster of 15 *N.submaculosa* specimens from Portugal, displaying a significant p-distance to the combined cluster, appears in the tree.

Diagnostic morphological characters of the two species are the black marking on the head, which is narrow with straight lateral margins in *N.submaculosa* and broad with rounded lateral margins in *N.flavescens*; the three black prescutal stripes, which are completely shiny in *N.submaculosa* and have a dull border for the medial stripe and dull anterior parts after the bend for the lateral stripes in *N.flavescens*; and the abdominal lateral black stripes, which are almost continuous in *N.submaculosa* and broken to single dots in *N.flavescens* (Stubbs 2021, Oosterbroek 2023).

**Comments.** Distinguishing between *N.submaculosa* and *N.flavescens* based on adult morphology poses a significant challenge, according to the first author's experience in identifying Tipulidae specimens. The diagnostic characters exhibit transitional variations without clear differentiation. Neither easily recognizable morphological features nor barcoding provides definitive separation between these species. Further molecular markers might be needed in combination with larval and adult morphology to diagnose these taxa.

#### Nephrotomacrocata (Linnaeus, 1758) and Nephrotomascalaris (Meigen, 1818)

The barcodes of specimens from *N.crocata* and *N.scalaris* form distinct clusters with a remarkably low minimal interspecific p-distance (0.46%; see Figure S1: Suppl. material 2). As a result, the two species clusters merge into one large distinctive cluster with a notably high minimal p-distance compared to any other barcode (6.08%). Within each species' cluster, the maximal intraspecific p-distance matches the minimal p-distance between both clusters (0.46%). All four species delimitation algorithms, categorize the barcode sequences of both species as part of a single entity (see Figs 6, 7).

After incorporating additional European tipulid COI barcodes from BOLD into the tree, an additional sequence from a Dutch *N.crocata* specimen emerges, neighboring the primary combined cluster of *N.crocata* and *N.scalaris* (see Figure S2: Suppl. material 4).

Morphological characteristics for distinguishing between the two species include: the head, mostly black with a yellow to orange spot in the center for *N.crocata*, while for *N.scalaris*, the head is mostly yellow with a small triangular spot; the curved part of the lateral stripe on the thorax back is matt in *N.crocata*, but glossy in *N.scalaris*; and the upper side of the abdomen features three to four almost straight transverse bands in *N.crocata*, compared to five to six more or less triangular bands in *N.scalaris* (Peeters and Oosterbroek 2013).

**Comments.** The exceptionally low interspecific p-distance suggests that relying solely on COI barcodes may not always definitively differentiate between *N.crocata* and *N.scalaris*. This hypothesis gains support from the outcome of all four species delimitation algorithms. Additionally, the inclusion of an additional Dutch *N.crocata* specimen from BOLD further indicates potential ambiguity in distinguishing these species based solely on their barcodes. Morphologically, however, the two species are easily discernible.

#### Prionocerasubserricornis (Zetterstedt, 1851) and Prionoceraturcica (Fabricius, 1787)

Within the dataset, there are two barcodes for each species, *P.subserricornis* and *P.turcica*. The barcodes of both species form distinct clusters, with very low intraspecific p-distances (0.30% in *P.subserricornis* and 0.46% in *P.turcica*; see Figure S1: Suppl. material 2). The minimal interspecific p-distance between COI sequences from each cluster is also relatively low (1.52%). All four applied species delimitation algorithms consistently classify the barcode sequences of both species as belonging to the same cluster (see Figs 6, 7).

When additional COI barcode sequences from other European tipulids are added to the *P.subserricornis*/*P.turcica* cluster, it results in a combined cluster containing four species of *Prionocera*. In this expanded cluster, all species' barcodes still form separate and distinct clusters with relatively low interspecific distances to each other. However, one Icelandic specimen's barcode of *P.turcica* clusters with the barcodes of *P.subserricornis* (see Figure S2: Suppl. material 4).

Both species are morphologically distinguishable primarily by the characters of the male genitalia. In *P.turcica*, tergite 9 exhibits four strongly pointed horizontal processes, with the inner pair positioned closely together and splayed. In contrast, in *P.pubescens*, tergite 9 features only two pointed processes, and the splayed projections are much further apart (Stubbs 2021).

**Comments.** Given the small sample size and the already low minimal interspecific p-distance, along with the species delimitation algorithms grouping both species as one, it is conceivable that distinguishing these two species solely based on their COI barcodes may be challenging. However, morphologically, these two species are easily identifiable and distinguishable by examining the shape of the male genitalia.

#### Tipulahortorum Linnaeus, 1758 and Tipula (Vestiplex) nubeculosa Meigen, 1804

The barcodes of the specimens of T. (V.) hortorum and T. (V.) nubeculosa form one shared unique cluster without any recognizable separation pattern (see Figure S1: Suppl. material 2).This clustering phenomenon is not attributed to poor sampling practices (all barcodes have a length of 658 bp), nor is it due to a small sample size (a total of 15 specimens' barcodes). All four applied species delimitation algorithms consistently classify the barcode sequences of both species as belonging to the same cluster (see Figs 6, 7).

The inclusion of sequences from four Bavarian specimens and three Finnish specimens of T. (V.) nubeculosa, as well as one Finnish specimen of T. (V.) hortorum from BOLD, does not alter the cluster's composition (see Figure S2: Suppl. material 4).

Morphologically, both species are primarily distinguishable by the characteristics of the male genitalia. In T. (V.) hortorum, the last tergite features short lateral lobes, and the last sternite bears processes on the hind apical corners, which are obliquely truncate and almost twin-spined at the apex. In contrast, in T. (V.) nubeculosa, the last tergite exhibits long sinuous lateral lobes, and the last sternite shows short, simple processes on the hind apical corners (Stubbs 2021).

**Comments.** T. (V.) nubeculosa and T. (V.) hortorum share the same haplotype in their COI barcode sequences, making them indistinguishable based on their barcodes. Morphologically, these two species are easily distinguishable and can be separated based on the shape of the male genitalia.

#### Tipula (Pterelachisus) varipennis Meigen, 1818 and Tipula (Pterelachisus) pseudovariipennis Czizek, 1912

Despite a relatively low minimal interspecific p-distance (1.52%) between the barcodes of the specimens of T. (P.) varipennis and T. (P.) pseudovariipennis, the clusters for both species are resolved separately (see Figure S1: Suppl. material 2). mPTP and BIN separate these species. Conversely, ASAP and TaxCI recognize these two species as a single entity; in fact, ASAP even groups T. (P.) submarmorata Schummel, 1833, within this single species classification (see Figs 6, 7).

Expanding the tree with additional barcodes of European tipulids (26 for T. (P.) varipennis, five for T. (P.) pseudovariipennis, and eight for T. (P.) submarmorata) maintains the same topology, despite TaxCI continuing to identify T. (P.) varipennis and T. (P.) pseudovariipennis as one species. However, the clusters for all three species remain consistent (see Figure S2: Suppl. material 4).

Morphologically, distinguishing between the two species relies on a combination of several often subtle characteristics. For instance, in T. (P.) varipennis, the third antennal segment is black, the eyes are widely separated beneath, and the front femora are black in the apical third. Additionally, the male inner clasper bears only a tiny spine, and the outer clasper is not excessively slender. In contrast, in T. (P.) pseudovariipennis, the third antennal segment is yellow, the eyes are less separated beneath, and the front femora are black only at the apex. Moreover, the male inner clasper features a strong dorsal spine, and the outer clasper is very slender (Stubbs 2021, Oosterbroek 2023).

**Comments.** Differentiating between T. (P.) varipennis and T. (P.) pseudovariipennis through barcoding is consistently achievable. Additionally, these two species are morphologically distinguishable and can be separated based on a combination of characteristics.

#### Tipula (Savtshenkia) staegeri Nielsen, 1922 and Tipula (Savtshenkia) subvafra Lackschewitz, 1936

One species delimitation algorithm, mPTP, groups the single barcode of the T. (S.) staegeri with the single barcode of T. (S.) subvafra, despite their high interspecific p-distance of 6.23% (see Figs 6, 7).

When including all European tipulid barcodes from BOLD, the barcode of T. (S.) staegeri clusters alongside another T. (S.) staegeri specimen from the UK, forming a distinct cluster notably distant from its nearest neighbor. On the other hand, the barcode of *T.subvafra* is next to a cluster of three barcodes from Finnish specimens of T. (S.) limbata Zetterstedt, 1838, displaying only a very low interspecific distance (see Figure S2: Suppl. material 4).

Morphologically, distinguishing males of T. (S.) staegeri and T. (S.) subvafra or T. (S.) limbata is straightforward. T. (S.) staegeri is easily identifiable as the only European species of T. (Savtshenkia) with very long paired median lobes on the last sternite (Oosterbroek 2023). However, distinguishing between T. (S.) subvafra and T. (S.) limbata presents challenges. Both species share a deep median cleft of sternite 9. Tipula (S.) subvafra is distinguished by its plain tergite 9, whereas T. (S.) limbata features a slightly cleft last tergite with lateral projections (Oosterbroek 2023).

**Comments.** Tipula (S.) staegeri and T. (S.) subvafra are easily distinguishable by their COI barcodes and morphological characteristics. However, it is questionable whether T. (S.) subvafra and T. (S.) limbata have very similar COI barcodes or whether either the one specimen of T. (S.) subvafra or the three specimens of T. (S.) limbata are misidentified. Given the morphological similarity between both species, both possibilities are plausible.

#### Tipula (Tipula) paludosa Meigen, 1830

The barcodes of the T. (T.) paludosa specimens form two distinct clusters, suggesting the presence of potentially two different species (see Figure S1: Suppl. material 2). Notably, within the cluster with three specimens, all individuals share an identical barcode. Intriguingly, one of these specimens was collected alongside others from the alternative cluster during the same week on the small island of Langeoog, Lower Saxony, Germany. The differentiation is supported by mPTP, BIN, and TaxCI analyses, all indicating two separate groupings. However, ASAP groups both clusters along with the single barcode of T. (T.) subcunctans Alexander, 1921 (see Figs 6, 7).

Expanding the dataset to include additional European tipulid specimens brings about significant changes in the topology. The barcode of T. (T.) subcunctans now forms a cluster alongside two Finnish and one Norwegian specimen of the same species. This cluster is adjacent to all T. (T.) paludosa barcodes, with the primary cluster remaining unchanged. The previously distinct T. (T.) paludosa barcodes now unite, forming a cluster inclusive of a misidentified specimen of T. (Acutipula) luna Westhoff, 1879, from the United Kingdom, along with eight male T. (T.) paludosa specimens from Portugal (see Figure S2: Suppl. material 4).

Morphologically, T. (T.) paludosa and T. (T.) subcunctans differ notably in antennal segment count, inter-eye distance, and subtle male genitalia characteristics (Oosterbroek 2023). T. (T.) paludosa is distinctive among these European species due to its unique feature of possessing 14 antennal segments, in contrast to the typical 13 in other species of T. (Tipula). All specimens identified as T. (T.) paludosa in this study exhibit this characteristic, at least among those retaining their antennae.

**Comments.** Given the morphological differentiation of T. (T.) paludosa and T. (T.) subcunctans, ASAP shows an evident case of overlumping. For the two T. (T.) paludosa clusters, despite an extensive morphological examination by the first author, no discernible differences were identified between the specimens, all of which align with the species description of T. (T.) paludosa. Notably, within Germany, no other alike species have been identified thus far, although several morphologically similar species exist across Europe, such as T. (T.) italica Lackschewitz, 1930 (Oosterbroek 2023). This implies the possibility that one of the T. (T.) paludosa clusters might represent either another already described species not known in Germany so far or an as-yet-undescribed, “cryptic” species. An integrative taxonomic revision is necessary to resolve this issue.

#### Tipula (Savtshenkia) pagana Meigen, 1818

The barcodes of the T. (S.) pagana specimens form two clusters, with a significantly high p-distance (5.47%) between them, each containing three barcodes (see Figure S1: Suppl. material 2). All four species delimitation algorithms identify the barcode sequences of T. (S.) pagana as belonging to two distinct species (see Figs 6, 7). Specimens from both clusters of T. (S.) pagana were collected in nearby locations within Hesse, Germany. One cluster includes a female with short wings.

Additional T. (S.) pagana barcodes from Finland and Norway cluster with the group of three specimens where the brachypterous female lies (see Figure S2: Suppl. material 4).

**Comments.** Intensive study by the first author failed to find morphological differences between the specimens from both clusters. The most morphologically similar species to T. (S.) pagana is T. (S.) holoptera Edwards, 1939. While well-documented in Great Britain and also known in Czechia, Slovakia, and Spain (Oosterbroek 2023), its occurrence in Germany has not been reported yet, although it is plausible. The definitive morphological distinction lies in T. (S.) pagana having brachypterous (short-winged) females, while T. (S.) holoptera possesses holopterous (fully-winged) females. The only other documented morphological difference is a slight variation in a male diagnostic character on the tip of the last sternite. However, previous documentation by Eiroa (1989) suggested variability in this character for T. (S.) holoptera, making it unreliable. Hypotheses by Theowald (1973) and Eiroa (1989) even suggested possible hybridization due to variability in this singular male diagnostic character, compounded by both species being collected from the same areas. Given that one three-specimen cluster contains a brachypterous female, these three specimens clearly represent T. (S.) pagana. The other three-specimen cluster might also represent T. (S.) pagana, with merely a high intraspecific molecular variability, or it could potentially belong to another species, such as T. (S.) holoptera. Additional barcoded specimens, especially of T. (S.) holoptera, could help to resolve this question.

#### Nephrotomaguestfalica (Westhoff, 1879)

One specimen within the five-specimen cluster of *N.guestfalica* exhibits a p-distance of 1.67% to its nearest neighbor from the same cluster (see Figure S1: Suppl. material 2). Both the species delimitation algorithms mPTP and TaxCI identify this isolated barcode as an additional taxon, while BIN and ASAP integrate it into the *N.guestfalica* cluster (see Figs 6, 7).

When we included additional barcodes from three Portuguese specimens in the analysis and applied the TaxCI script, all *N.guestfalica* barcodes were consistently classified as belonging to a single species (see Figure S2: Suppl. material 4).

**Comments.** After morphologically re-examining the questionable specimen by the first author, no differences from the species description of *N.guestfalica* were detectable. It seems that mPTP and TaxCI might exhibit oversplitting, possibly due to the small sample size and variability in the COI barcode of the species.

#### Tipula (Lunatipula) lunata Linnaeus, 1758

One specimen within the cluster of 39 specimens of T. (L.) lunata exhibits a p-distance of 1.37% to its nearest neighbor from the same cluster (see Figure S1: Suppl. material 2). Both the species delimitation algorithms mPTP and BIN identify this isolated barcode as an additional taxon, while ASAP and TaxCI integrate it into the T. (L.) lunata cluster (see Figs 6, 7).

**Comments.** After morphologically re-examining the questionable specimen, the first author found no differences from the species description of T. (L.) lunata. Most probably, this is a case of oversplitting by mPTP and BIN, likely due to the variability in the COI barcode of the species.

#### Tipula (Pterelachisus) sp. (aff. *trifascingulata*, *irrorata*, *submarmorata*, *varipennis*, *pseudovariipennis*)

The single barcoded female specimen of T. (Pterelachisus) sp. could not be identified to the species level. We decided to include it in the dataset because it belongs to the subgenus T. (Pterelachisus), but it does not correspond to any of the other species within this subgenus found in our dataset. These species include T. (P.) trifascingulata, T. (P.) irrorata Macquart, 1826, T. (P.) submarmorata, T. (P.) varipennis, and T. (P.) pseudovariipennis. The mPTP algorithm recognizes both *Tipula* sp. and *T.trifascingulata* as belonging to the same species, while BIN, ASAP and TaxCI recognize *Tipula* sp. as a separate species (see Figs 6, 7).

In the combined tree of the barcodes from the GBOL specimens and additional barcodes from European tipulids, the barcode of *Tipula* sp. remains isolated, neighboring a cluster of four barcodes from specimens of T. (P.) cinereocincta Lundstrom, 1907 from Finland (see Figure S2: Suppl. material 4).

**Comments.** Morphologically, distinguishing between females of T. (Pterelachisus) can be challenging, as many lack diagnostic characters. Based solely on morphology, *Tipula* sp. could be identified as a female of T. (P.) trifascingulata. However, the high p-distance observed between the barcodes of *Tipula* sp. and T. (P.) trifascingulata (5.93%) suggests that *Tipula* sp. is more likely to belong to another European T. (Pterelachisus) species not yet represented in BOLD.

## Discussion

The BIN algorithm achieved the highest rate mirroring our morphologically identified species for the GBOL Tipulidae dataset, with a match ratio of 86% of the 76 studied species. By analyzing the results of all four species identification algorithms (mPTP, BIN, ASAP, and TaxCI) together, and by cross-referencing these results with additional European tipulid barcodes from BOLD, we were able to unmistakably identify (in other words, assign COI barcodes to species identified morphologically) 68 out of 76 species using DNA barcodes. Among the 68 morphological species with unequivocally molecular correlation two species exhibited more than one DNA barcode cluster, warranting further investigation with a broader sampling, and two species clusters displayed outlier barcodes, each identified by two species delimitation algorithms as different species. This behavior for the outlier barcodes is likely attributable to oversplitting by the algorithms. Only four species pairs (eight morphological species in total) were not clearly separable by COI barcodes in our GBOL Tipulidae dataset. In addition, one species of these not clearly separable species pairs also exhibited multiple species clusters.

Our success rate of 89% (68 out of 76 species) aligns with similar arthropod barcoding studies within the German Barcode of Life project (GBOL), such as Raupach et al. 2014 (Heteroptera, 1,742 specimens of 457 species, 92% success rate), Morinière et al. 2014 (Neuropterida, 237 specimens of 83 species, 90% success rate), Hendrich et al. 2014 (Coleoptera, 15,948 specimens of 3,514 species, 92% success rate), Schmid‐Egger et al. 2018 (Hymenoptera: Ampulicidae, Crabronidae, and Sphecidae, 3,695 specimens of 661 species, 99% success rate), and Geiger et al. 2021 (Odonata, 697 specimens of 103 species, 88% success rate).

Despite taking more than a decade to build, our database still lacks more than 45% of the recorded species for Germany. Nevertheless, it represents a significant improvement over previously-published data. Our 824 newly-sequenced high-quality COI barcodes, available on BOLD, complement the existing 696 published barcodes with a length of at least 550 bp for European tipulid specimens. This extends the database for high-quality barcodes of European tipulid specimens by 118% and contributes to enhancing future barcode identification requests for the database.

Additionally, through using the TaxCI script analyzing the BINs assigned to the sequences instead of the species names, we devised and tested a simple method to help identify potential misidentifications in large datasets.

## Supplementary Material

C6799371-1E2E-57D7-B1E1-F008250CB278 10.3897/BDJ.12.e127190.suppl1 Supplementary material 1 Table S1.1-S1.6 Data type tables Brief description Sex distribution, life stage distribution, collection countries, collection years, collection methods, and storage methods for the 824 specimens of the GBOL Tipulidae. File: oo_1043139.xlsx https://binary.pensoft.net/file/1043139 Moritz Fahldieck, Björn Rulik, Jana Thormann, Ximo Mengual

FB4A78A1-135A-502F-9321-E887292637E5 10.3897/BDJ.12.e127190.suppl2 Supplementary material 2 Figure S1 Data type tree Brief description TaxCI tree of the 824 specimens of the GBOL Tipulidae. File: oo_1043151.pdf https://binary.pensoft.net/file/1043151 Moritz Fahldieck, Björn Rulik, Jana Thormann, Ximo Mengual

91D31616-DD95-5FA7-AE30-E03F3CD64C3F 10.3897/BDJ.12.e127190.suppl3 Supplementary material 3 Table S2.1-S2.3 Data type tables Brief description Sex distribution, life stage distribution and collection countries of the 696 specimens of the European Tipulidae. File: oo_1043140.xlsx https://binary.pensoft.net/file/1043140 Moritz Fahldieck, Björn Rulik, Jana Thormann, Ximo Mengual

EB294194-E0DC-57D5-B0FB-92C3C7BB9F24 10.3897/BDJ.12.e127190.suppl4 Supplementary material 4 Figure S2 Data type tree Brief description Combined TaxCI tree of the 824 specimens of the GBOL Tipulidae and of the 696 specimens of the European Tipulidae. File: oo_1043154.pdf https://binary.pensoft.net/file/1043154 Moritz Fahldieck, Björn Rulik, Jana Thormann, Ximo Mengual

AD72E875-601A-52C7-B7A0-0EC1940B1ACD 10.3897/BDJ.12.e127190.suppl5 Supplementary material 5 Figure S3 Data type tree Brief description TaxCI tree of the 824 specimens of the GBOL Tipulidae scrutinized by their Barcode Index Numbers (BINs). File: oo_1043156.pdf https://binary.pensoft.net/file/1043156 Moritz Fahldieck, Björn Rulik, Jana Thormann, Ximo Mengual

3454D319-F4BA-58F8-B3BB-D7A54175CC98 10.3897/BDJ.12.e127190.suppl6 Supplementary material 6 Figure S4 Data type tree Brief description Combined TaxCI tree of the 824 specimens of the GBOL Tipulidae and of the 696 specimens of the European Tipulidae scrutinized by their Barcode Index Numbers (BINs). File: oo_1043158.pdf https://binary.pensoft.net/file/1043158 Moritz Fahldieck, Björn Rulik, Jana Thormann, Ximo Mengual

235AE5EA-16ED-5568-9802-FB81C72AE726 10.3897/BDJ.12.e127190.suppl7 Supplementary material 7 Figure S5 Data type tree Brief description Combined TaxCI tree of the 824 specimens of the GBOL Tipulidae and of 688 specimens of the European Tipulidae, with eight clearly misidentified specimens removed. File: oo_1043159.pdf https://binary.pensoft.net/file/1043159 Moritz Fahldieck, Björn Rulik, Jana Thormann, Ximo Mengual

A8EB4E56-B542-505E-9BCE-C6B444172C86 10.3897/BDJ.12.e127190.suppl8 Supplementary material 8 Table S3 Data type table Brief description Intra- and interspecific p-distances among the 76 species of the GBOL Tipulidae. File: oo_1043142.xlsx https://binary.pensoft.net/file/1043142 Moritz Fahldieck, Björn Rulik, Jana Thormann, Ximo Mengual

1AE61CC6-8EBC-5F51-854D-F41F2C840A09 10.3897/BDJ.12.e127190.suppl9 Supplementary material 9 Table S4 Data type table Brief description Pairwise p-distances among all 824 specimens of the GBOL Tipulidae. File: oo_1043144.xlsx https://binary.pensoft.net/file/1043144 Moritz Fahldieck, Björn Rulik, Jana Thormann, Ximo Mengual

8350E052-E362-5B62-90F6-5922A47B1254 10.3897/BDJ.12.e127190.suppl10 Supplementary material 10 Appendix 1 Data type remarks Brief description Species-specific remarks for the GBOL Tipulidae. File: oo_1043150.pdf https://binary.pensoft.net/file/1043150 Moritz Fahldieck, Björn Rulik, Jana Thormann, Ximo Mengual

## Figures and Tables

**Table 1.** Match ratios of the species delimitation methods mPTP, BIN, ASAP, and TaxCI applied to the sequences of the 824 specimens of the GBOL Tipulidae.

	**mPTP**	**BIN**	**ASAP**	**TaxCI**
**Nmatch**	58	65	61	63
**Nmol**	77	76	71	74
**match ratio**	76%	86%	83%	84%
Nmorph = 76				
match ratio = 2 × Nmatch/(Nmol + Nmorph)				
