# Supplementary material for: A DNA barcode reference library for the Tipulidae (Insecta, Diptera) of Germany
Source: Biodivers Data J. 2024 Sep 24;12:e127190. doi: 10.3897/BDJ.12.e127190 (PMC11445608; doi:10.3897/BDJ.12.e127190)
Supplement: Supplementary material 4 — Figure S2 [file bdj-12-e127190-s004.pdf]

Figure S2. Combined TaxCI tree of the 824 specimens of the GBOL Tipulidae
and of the 696 specimens of the European Tipulidae.

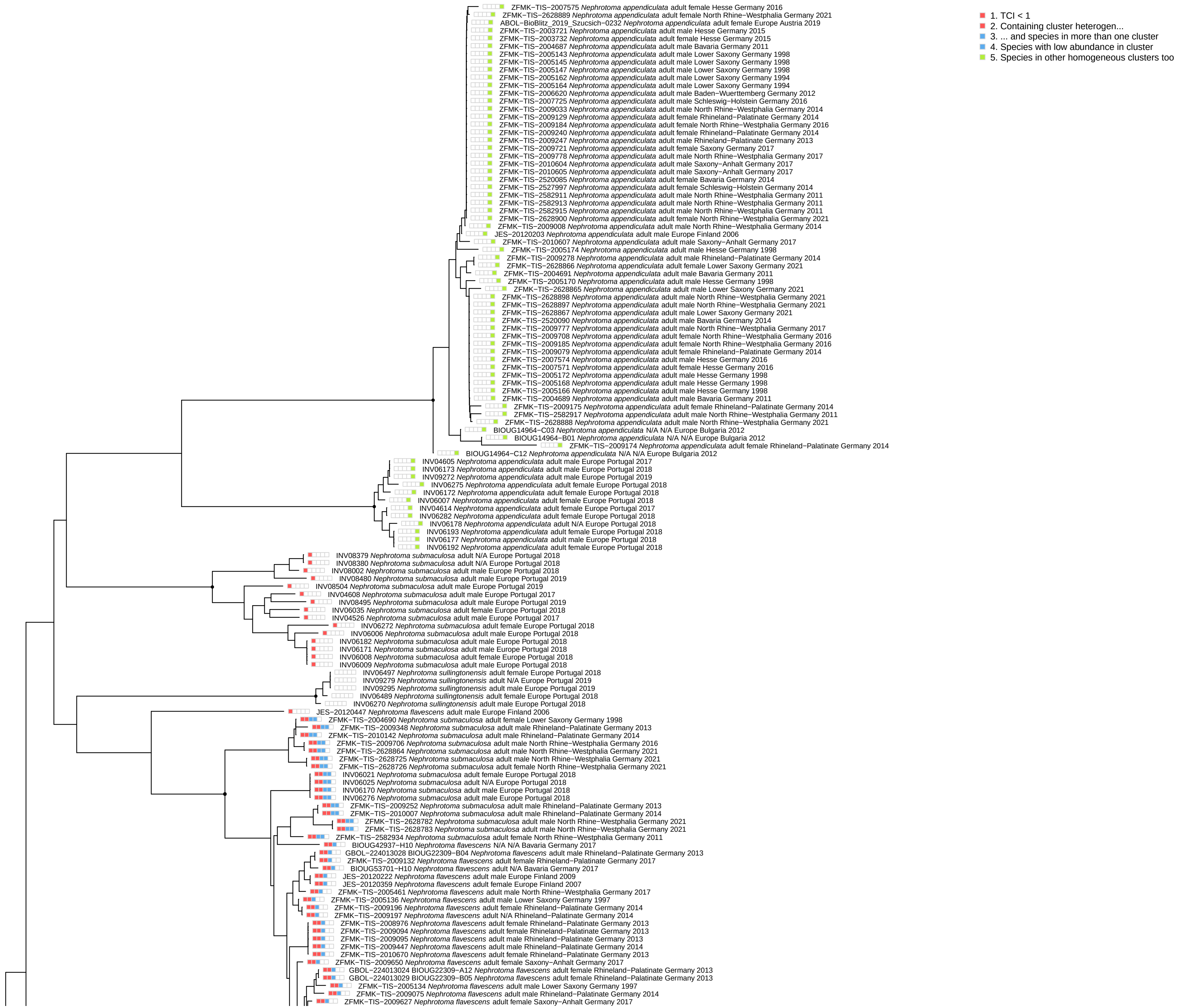

Fahldieck et al. 2024: Figure S2 (1520), A priori determined species: 154, Barcode cluster: 151, Threshold: 0.0247

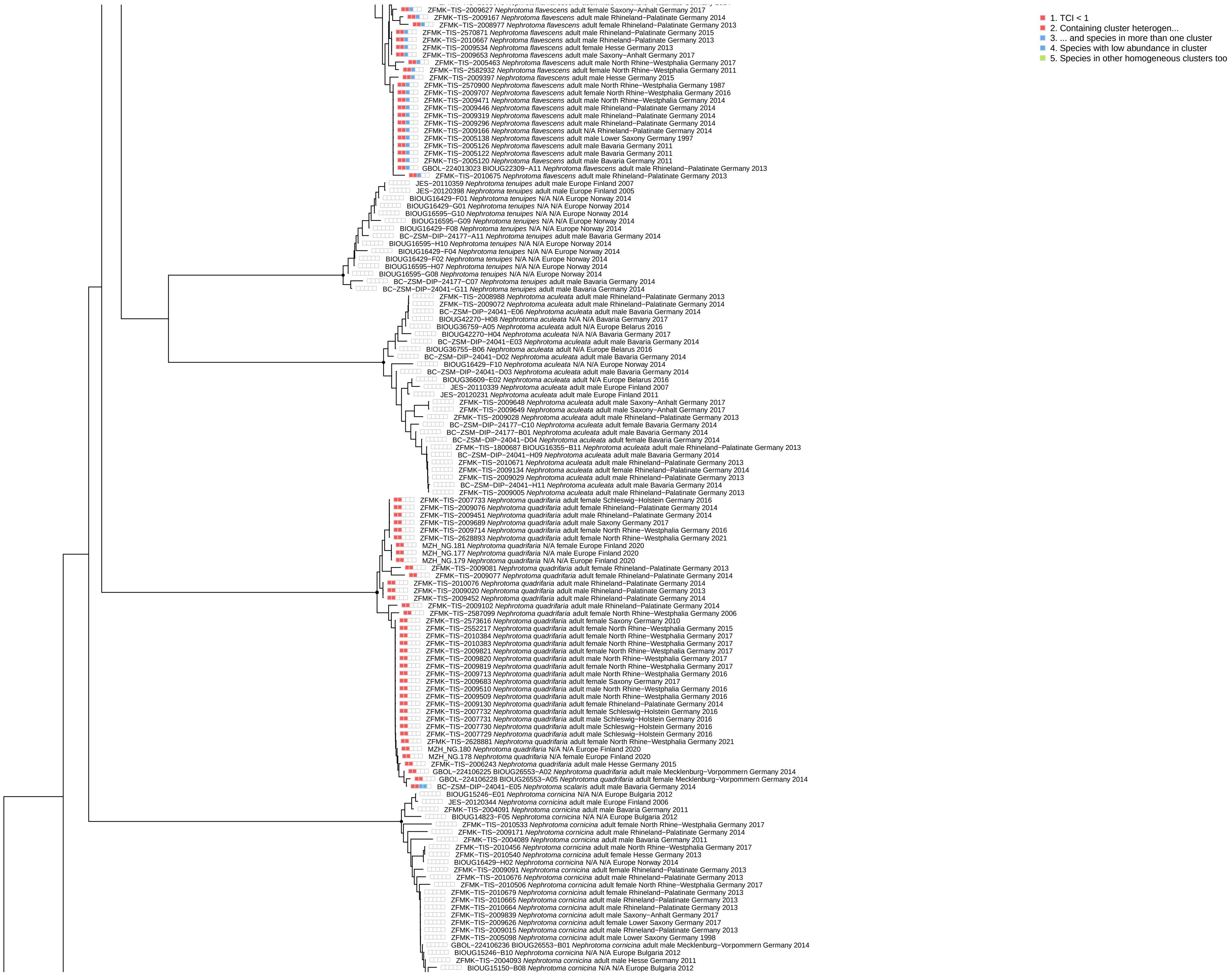

Fahldieck et al. 2024: Figure S2 (1520), A priori determined species: 154, Barcode cluster: 151, Threshold: 0.0247

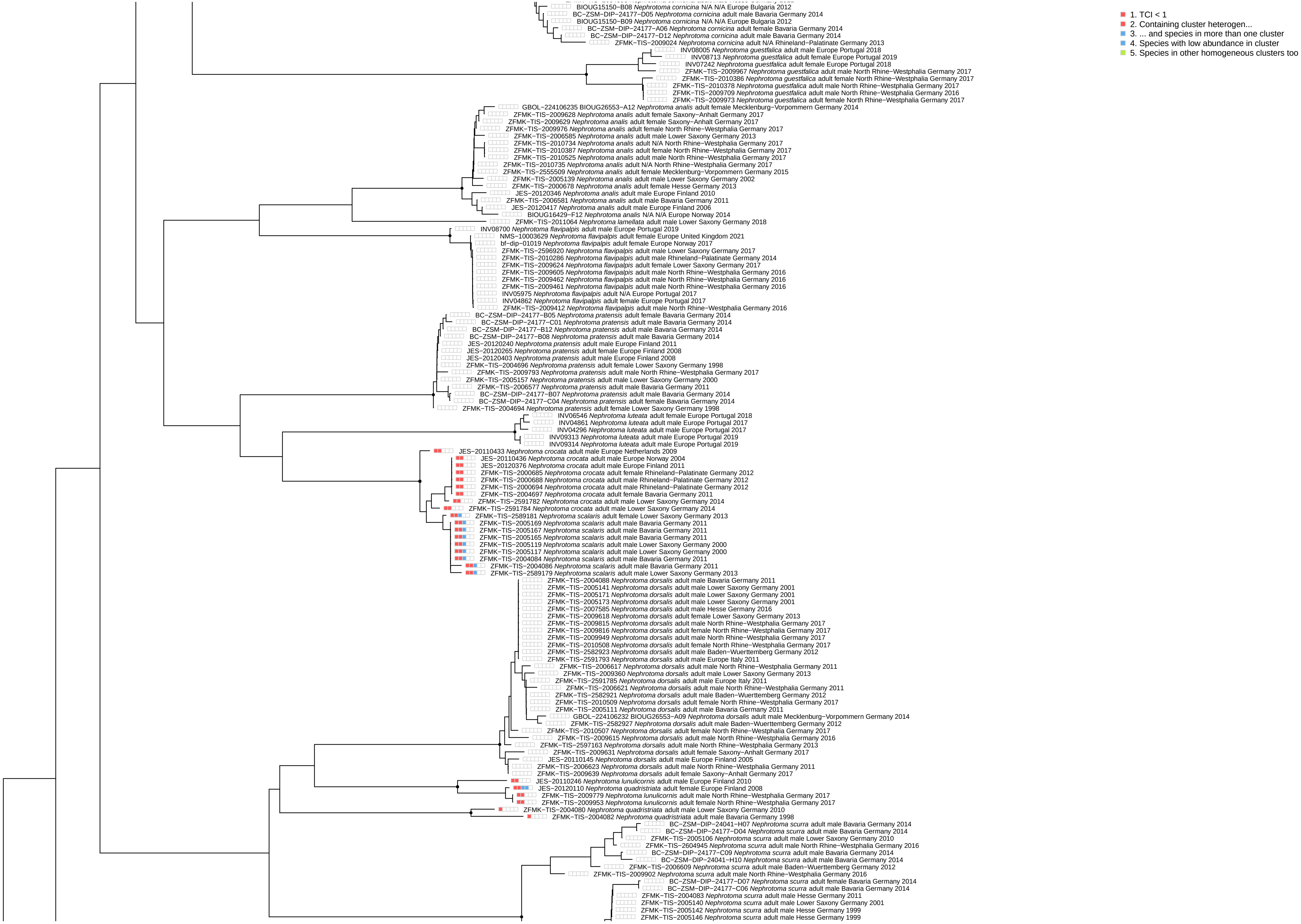

Fahldieck et al. 2024: Figure S2 (1520), A priori determined species: 154, Barcode cluster: 151, Threshold: 0.0247

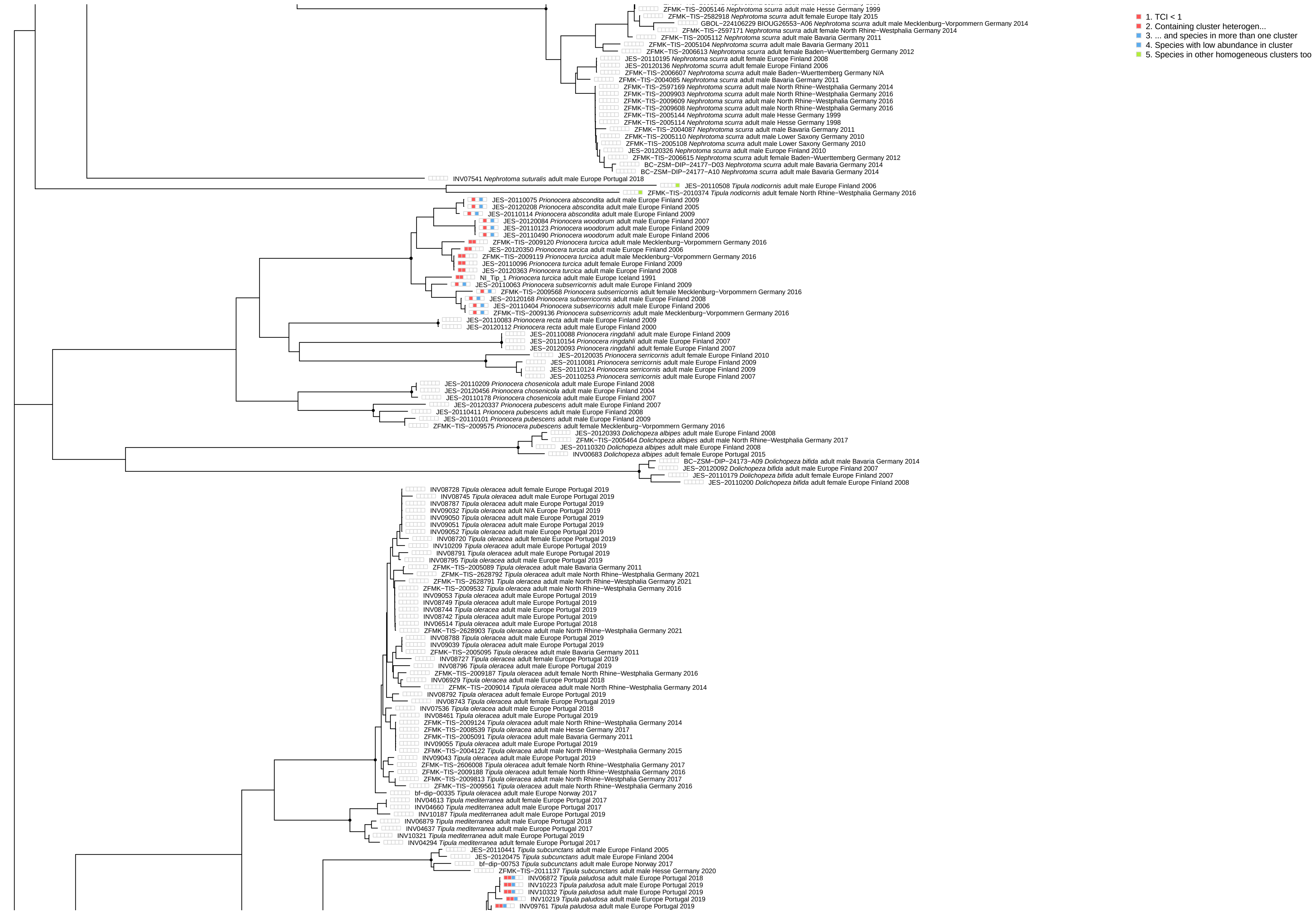

Fahldieck et al. 2024: Figure S2 (1520), A priori determined species: 154, Barcode cluster: 151, Threshold: 0.0247

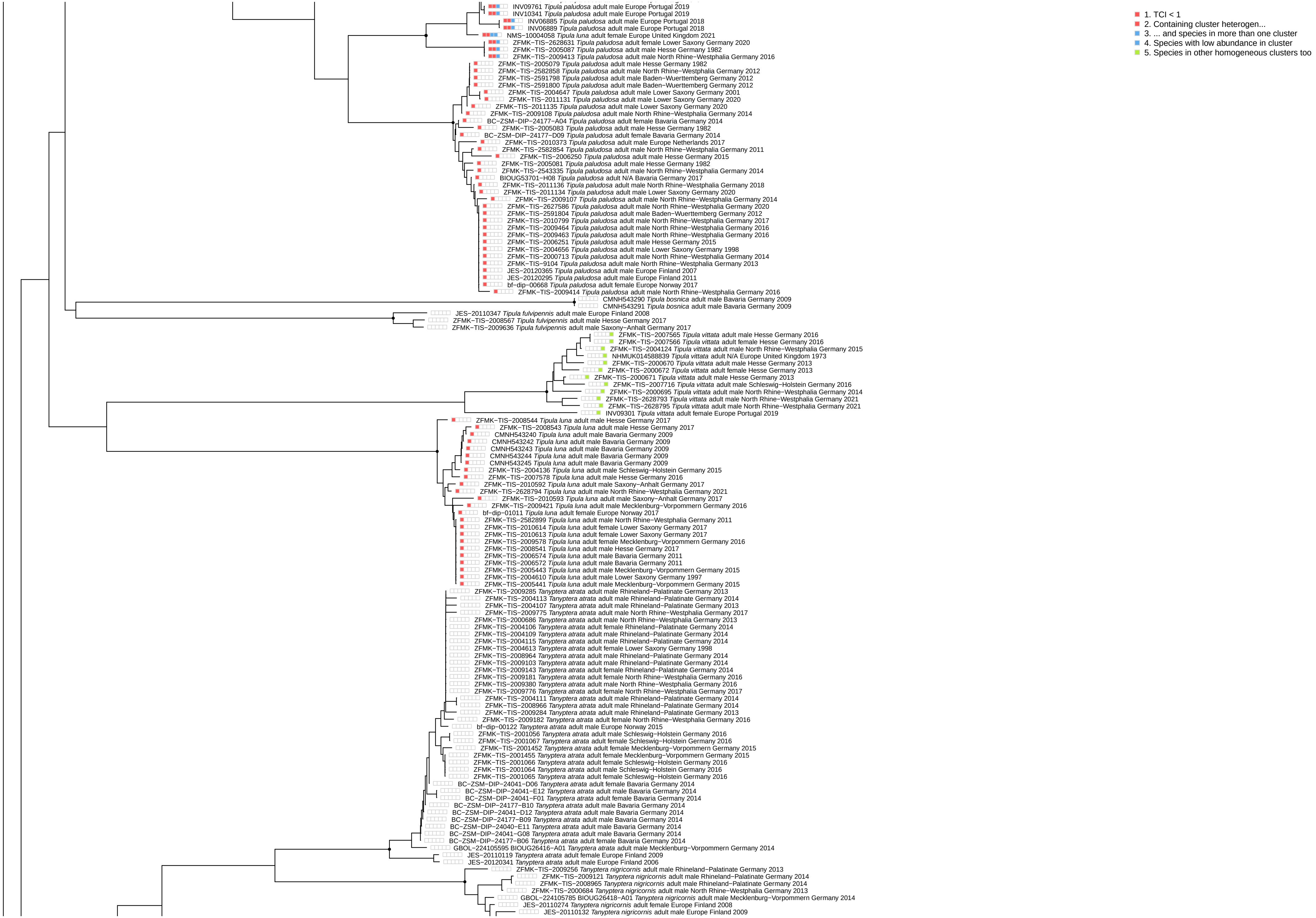

Fahldieck et al. 2024: Figure S2 (1520), A priori determined species: 154, Barcode cluster: 151, Threshold: 0.0247

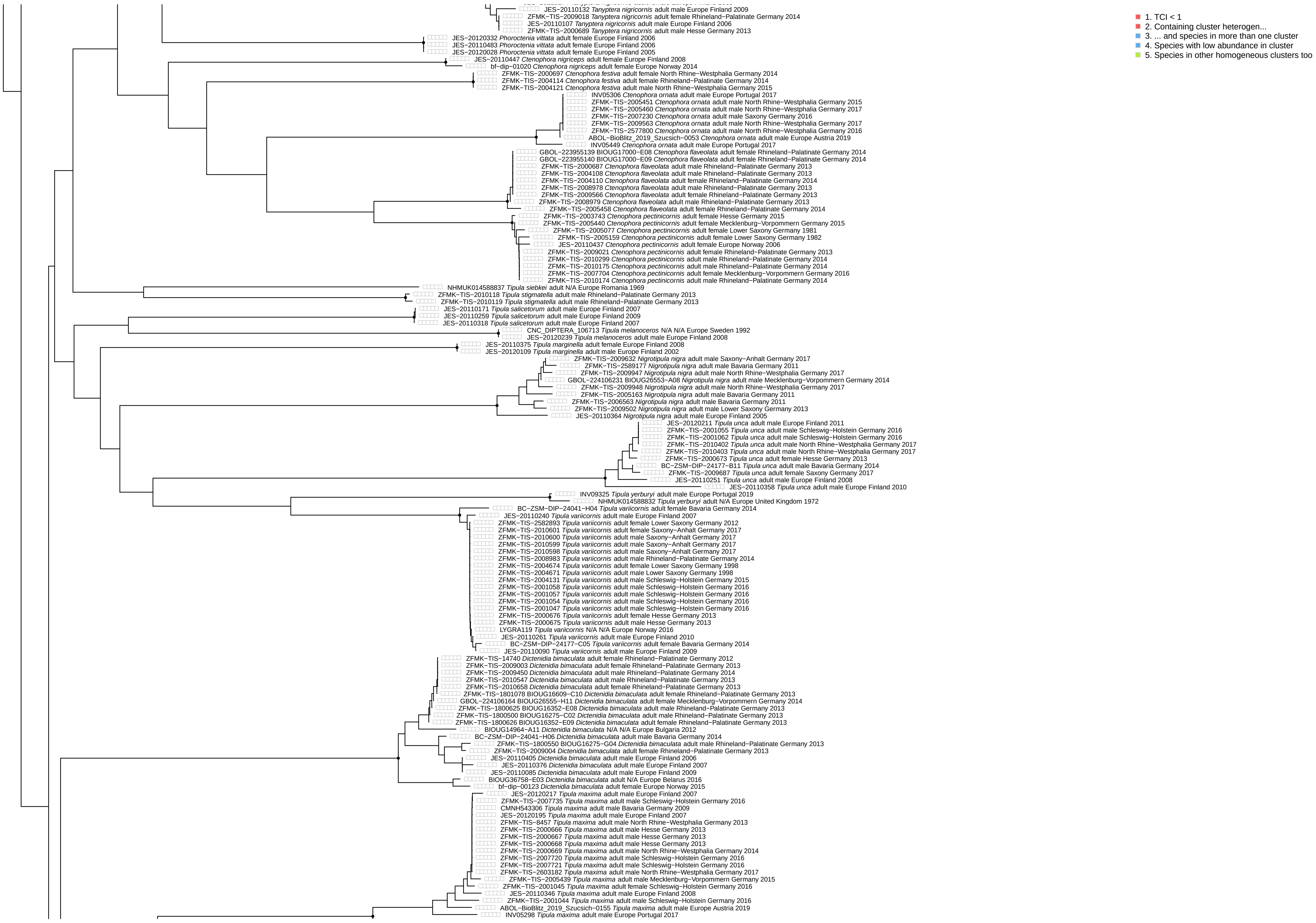

Fahldieck et al. 2024: Figure S2 (1520), A priori determined species: 154, Barcode cluster: 151, Threshold: 0.0247

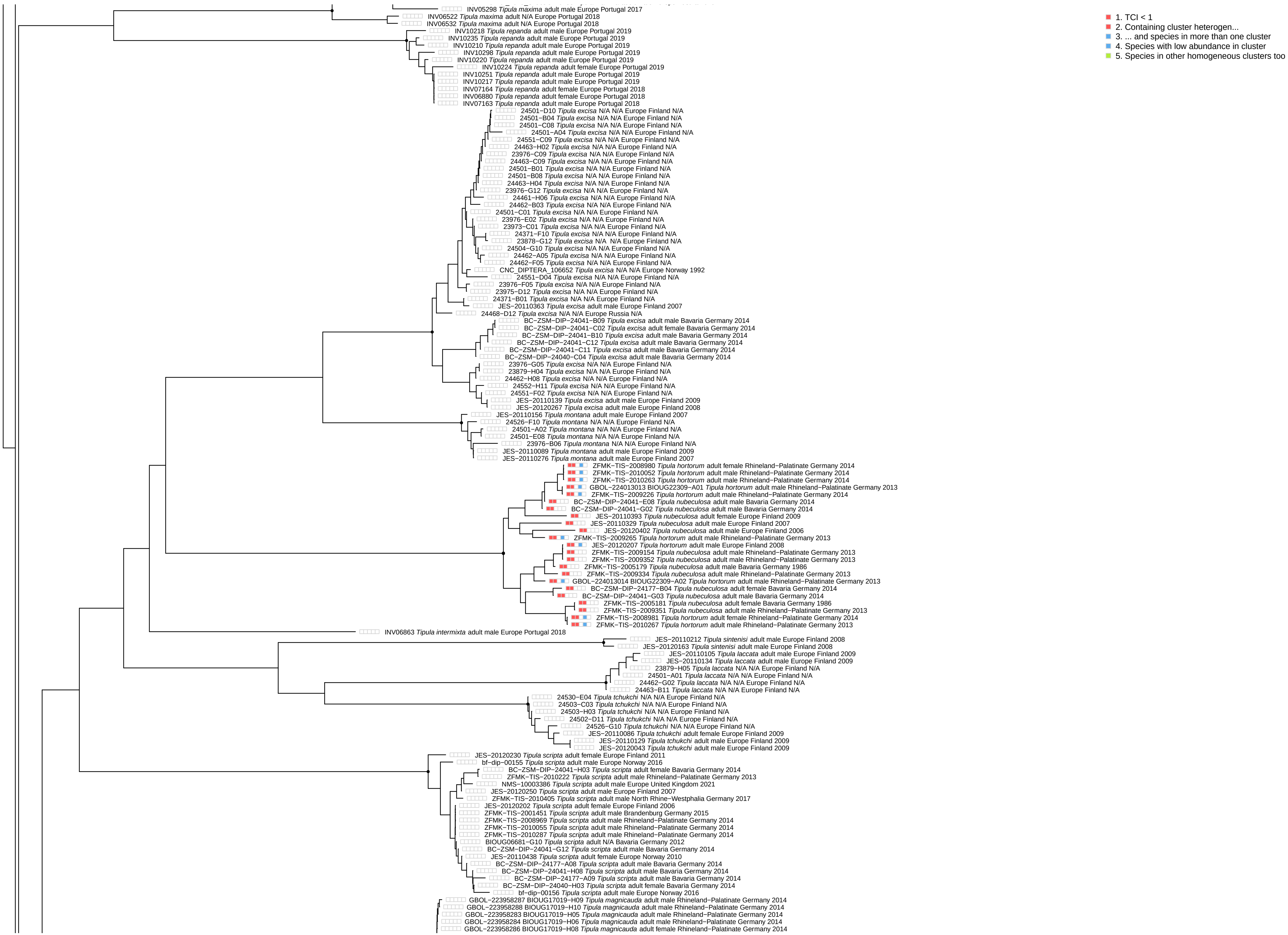

Fahldieck et al. 2024: Figure S2 (1520), A priori determined species: 154, Barcode cluster: 151, Threshold: 0.0247

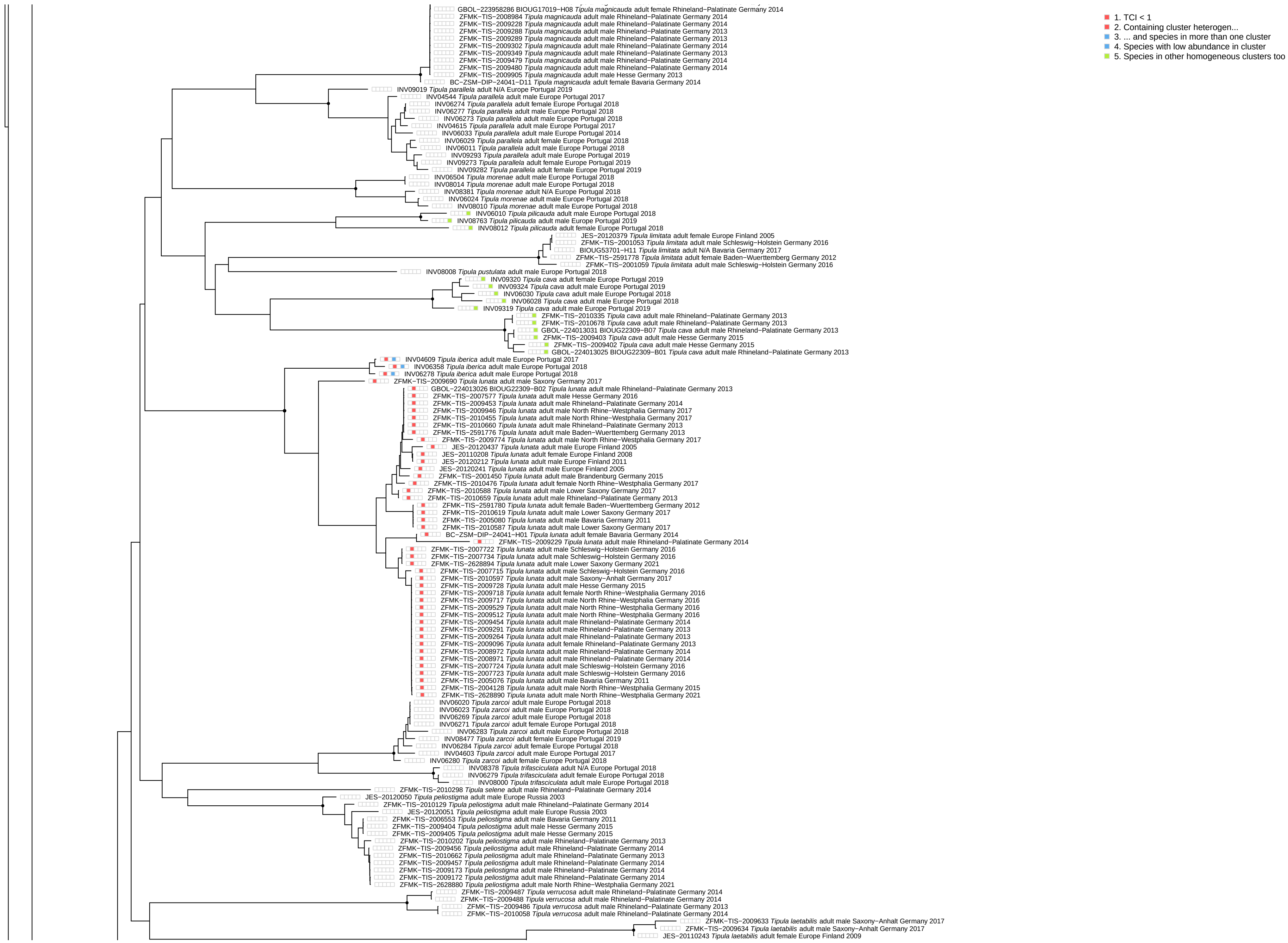

Fahldieck et al. 2024: Figure S2 (1520), A priori determined species: 154, Barcode cluster: 151, Threshold: 0.0247

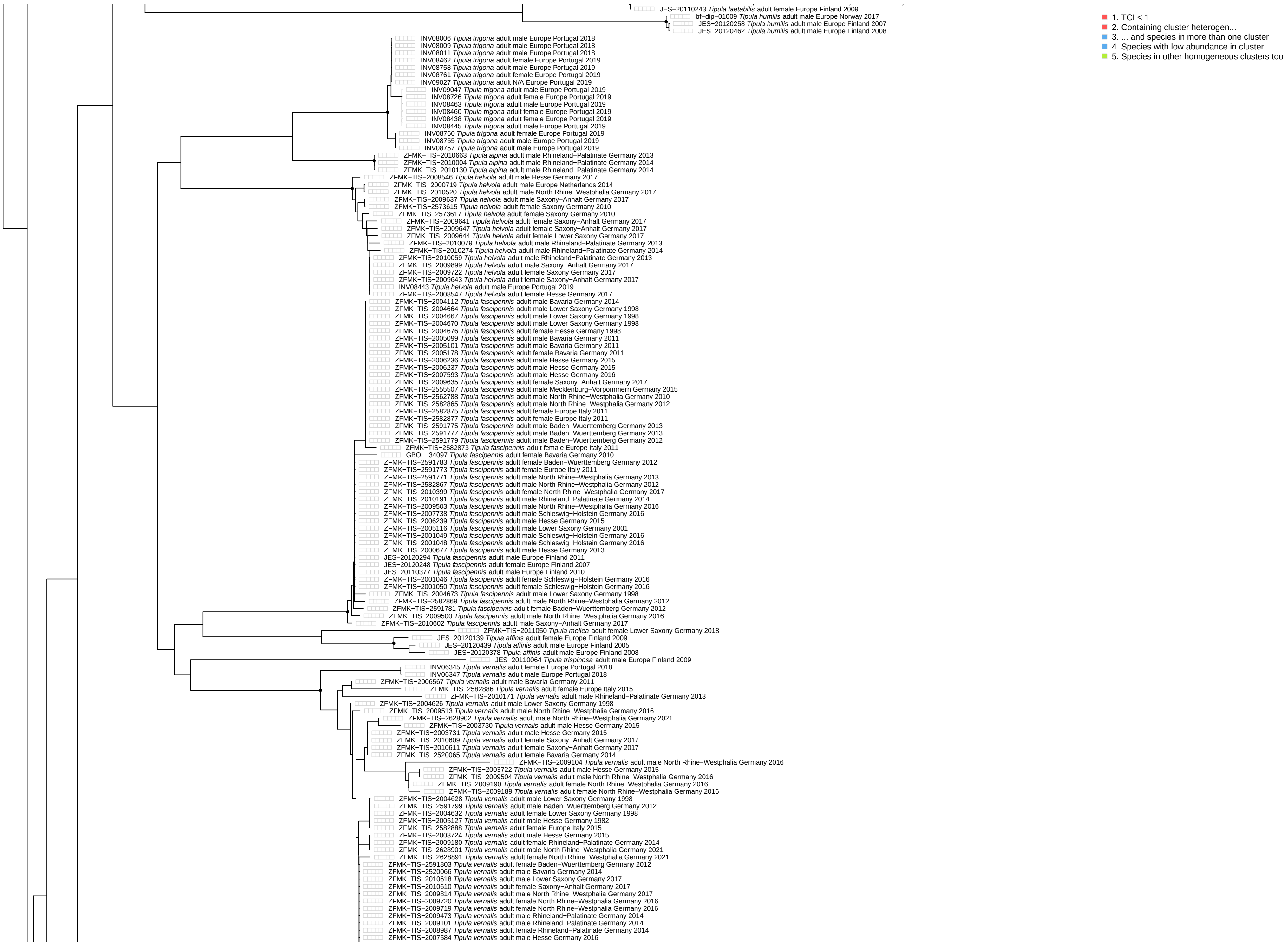

Fahldieck et al. 2024: Figure S2 (1520), A priori determined species: 154, Barcode cluster: 151, Threshold: 0.0247

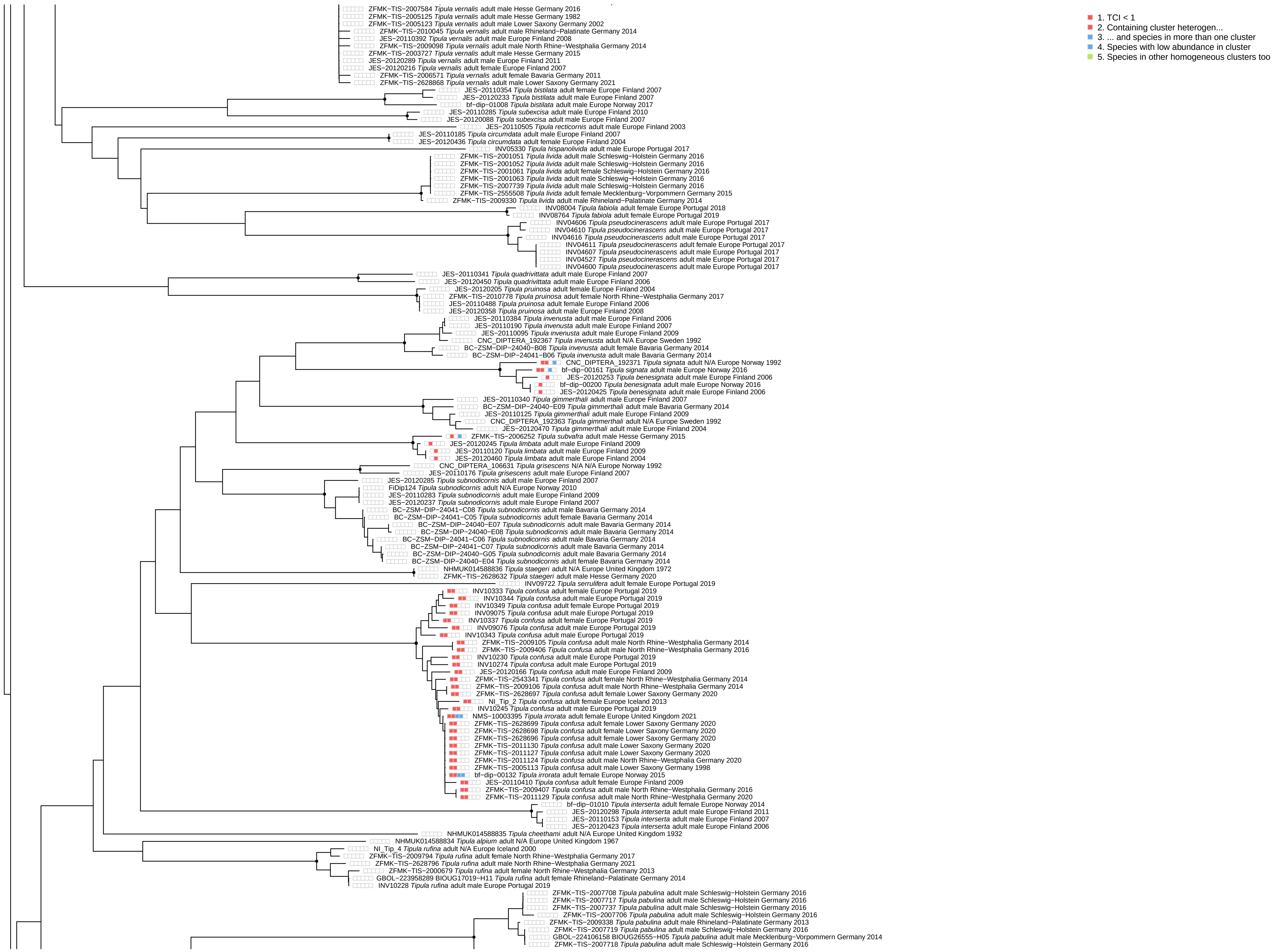

Fahldieck et al. 2024: Figure S2 (1520), A priori determined species: 154, Barcode cluster: 151, Threshold: 0.0247

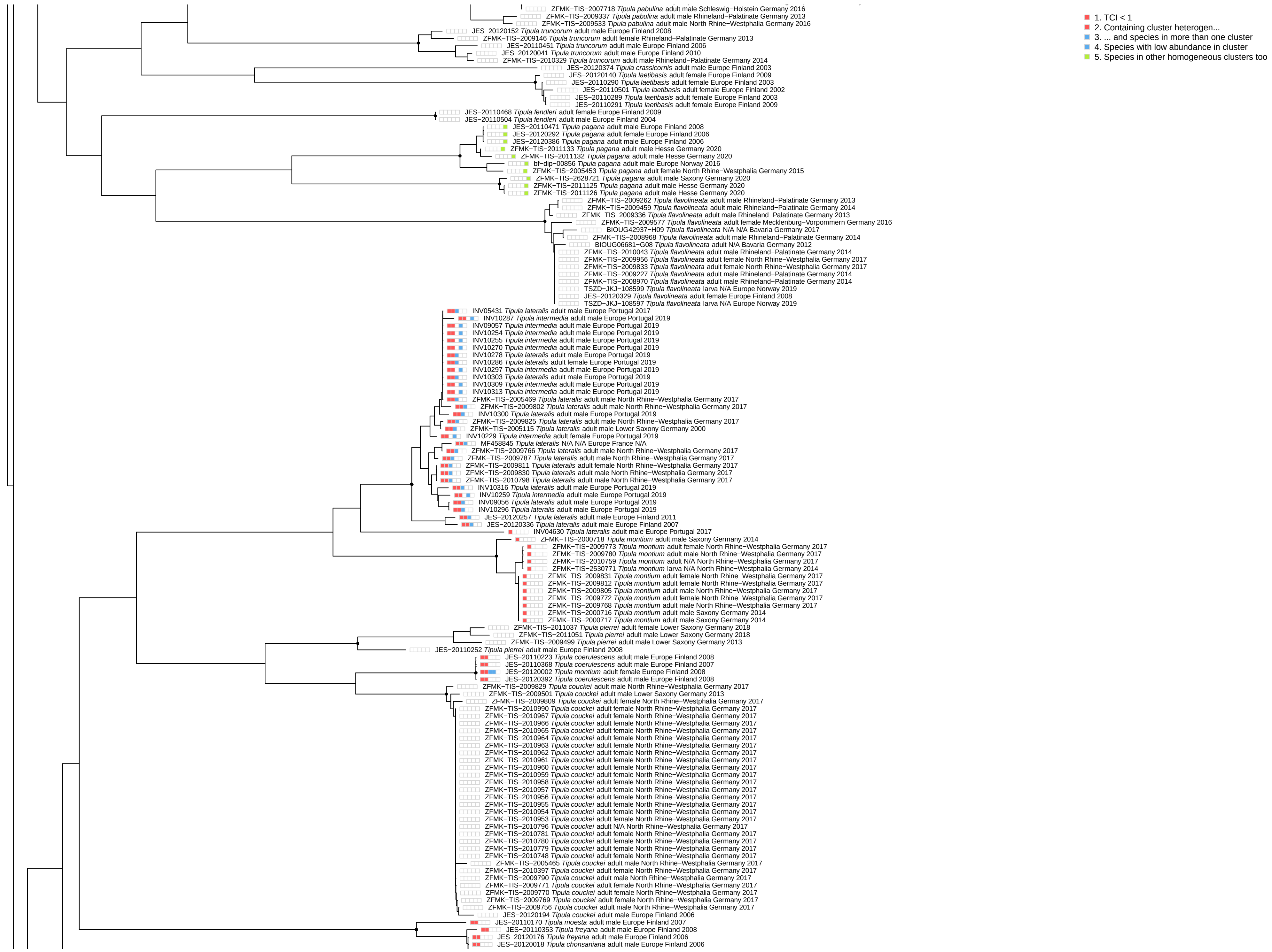

Fahldieck et al. 2024: Figure S2 (1520), A priori determined species: 154, Barcode cluster: 151, Threshold: 0.0247

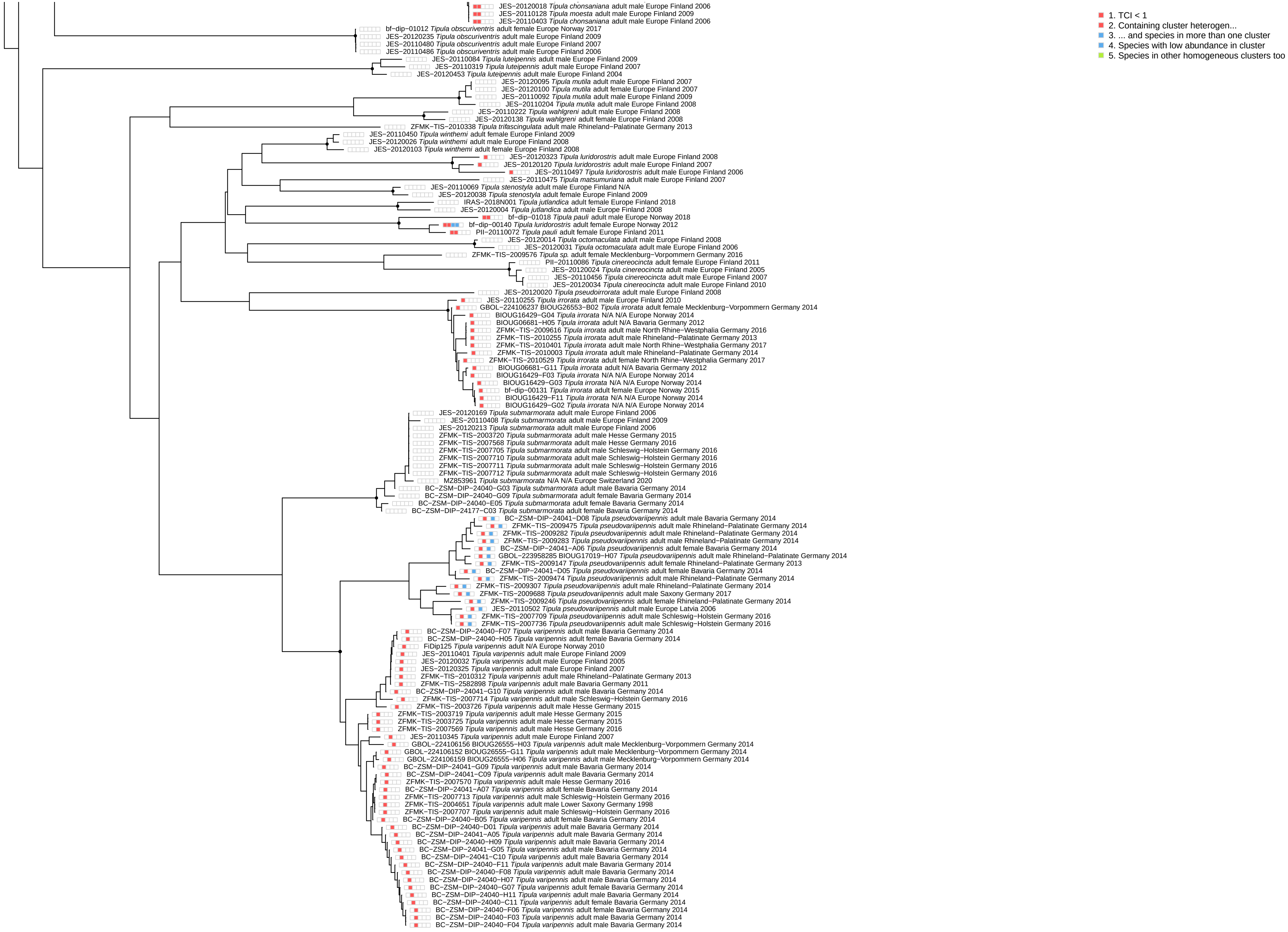

Fahldieck et al. 2024: Figure S2 (1520), A priori determined species: 154, Barcode cluster: 151, Threshold: 0.0247
