# Supplementary material for: A DNA barcode reference library for the Tipulidae (Insecta, Diptera) of Germany
Source: Biodivers Data J. 2024 Sep 24;12:e127190. doi: 10.3897/BDJ.12.e127190 (PMC11445608; doi:10.3897/BDJ.12.e127190)
Supplement: Supplementary material 5 — Figure S3 [file bdj-12-e127190-s005.pdf]

Figure S3. TaxCI tree of the 824 specimens of the GBOL Tipulidae scrutinized by their Barcode Index Numbers (BINs).

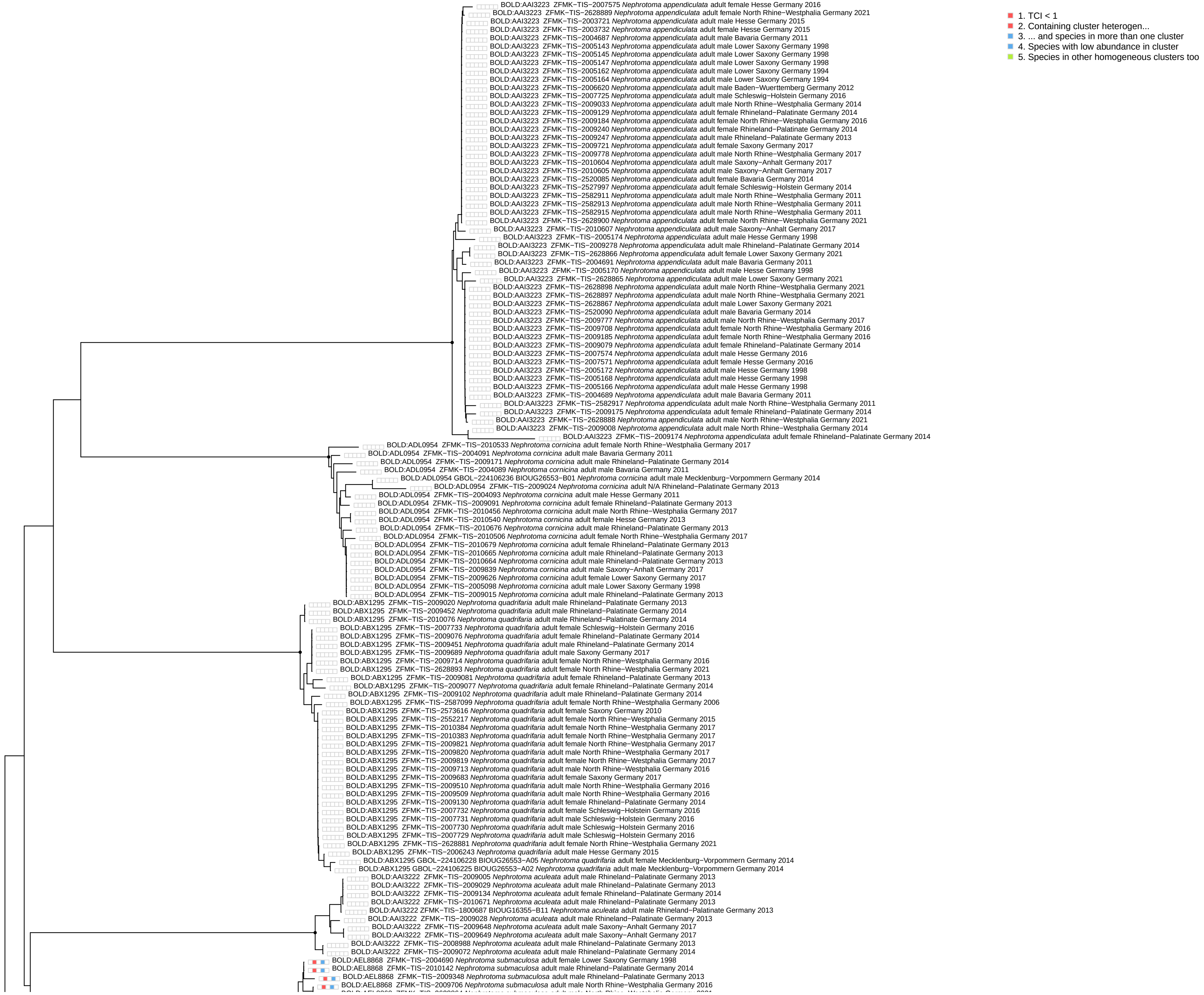

Fahldieck et al. 2024: Figure S3 (824_BINs), A priori determined species: 76, Barcode cluster: 74, Threshold: 0.0155

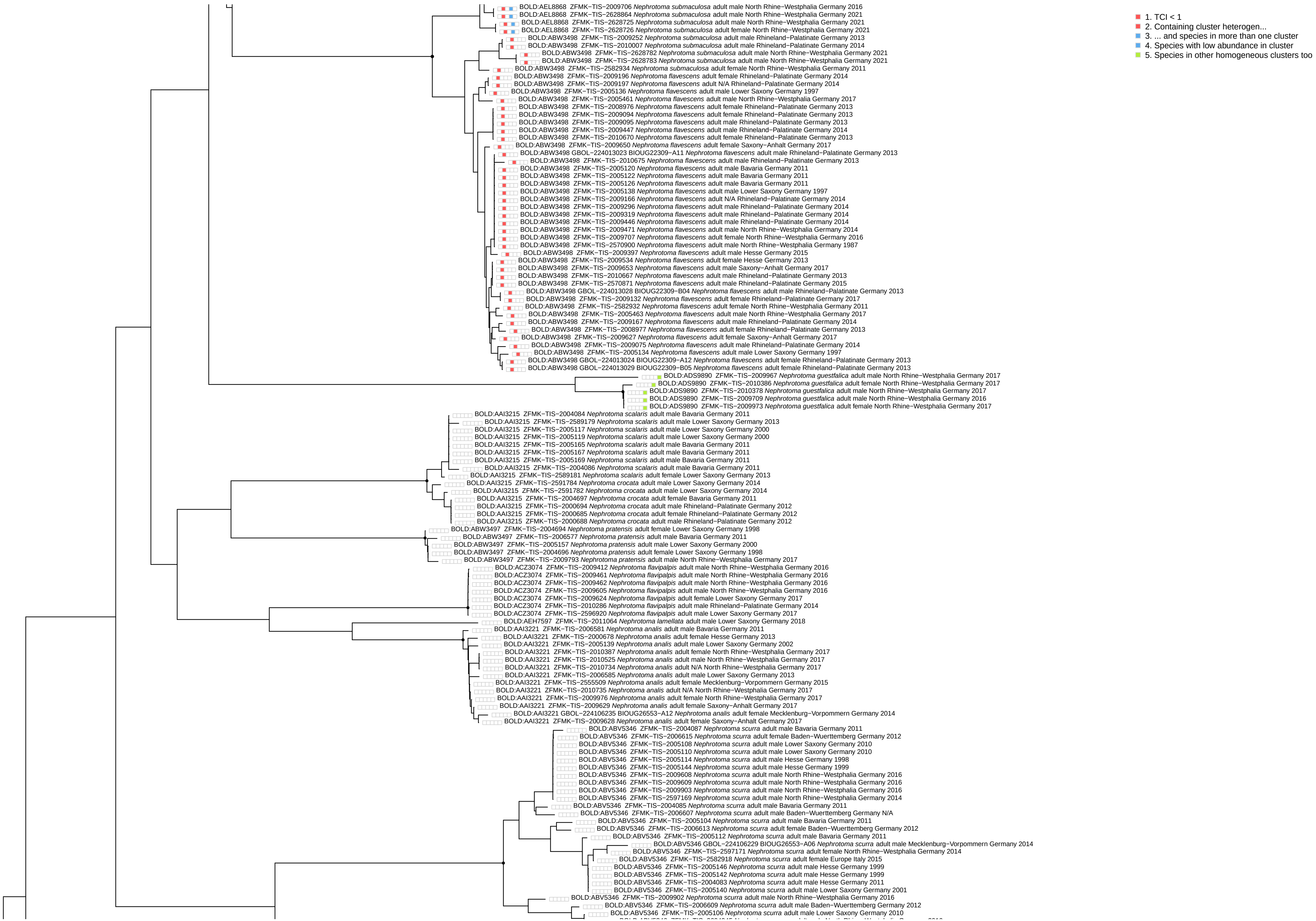

Fahldieck et al. 2024: Figure S3 (824_BINs), A priori determined species: 76, Barcode cluster: 74, Threshold: 0.0155

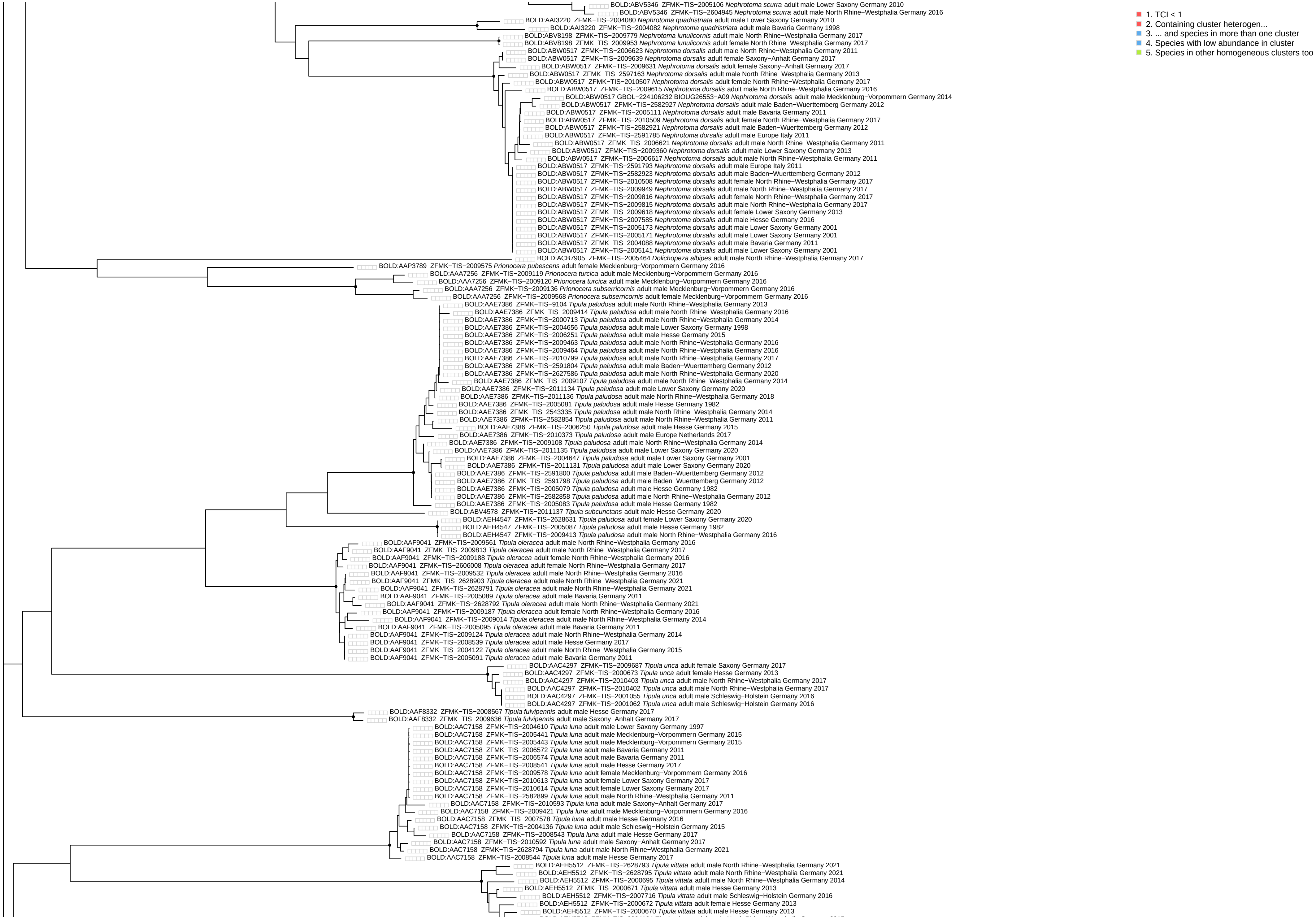

Fahldieck et al. 2024: Figure S3 (824_BINs), A priori determined species: 76, Barcode cluster: 74, Threshold: 0.0155

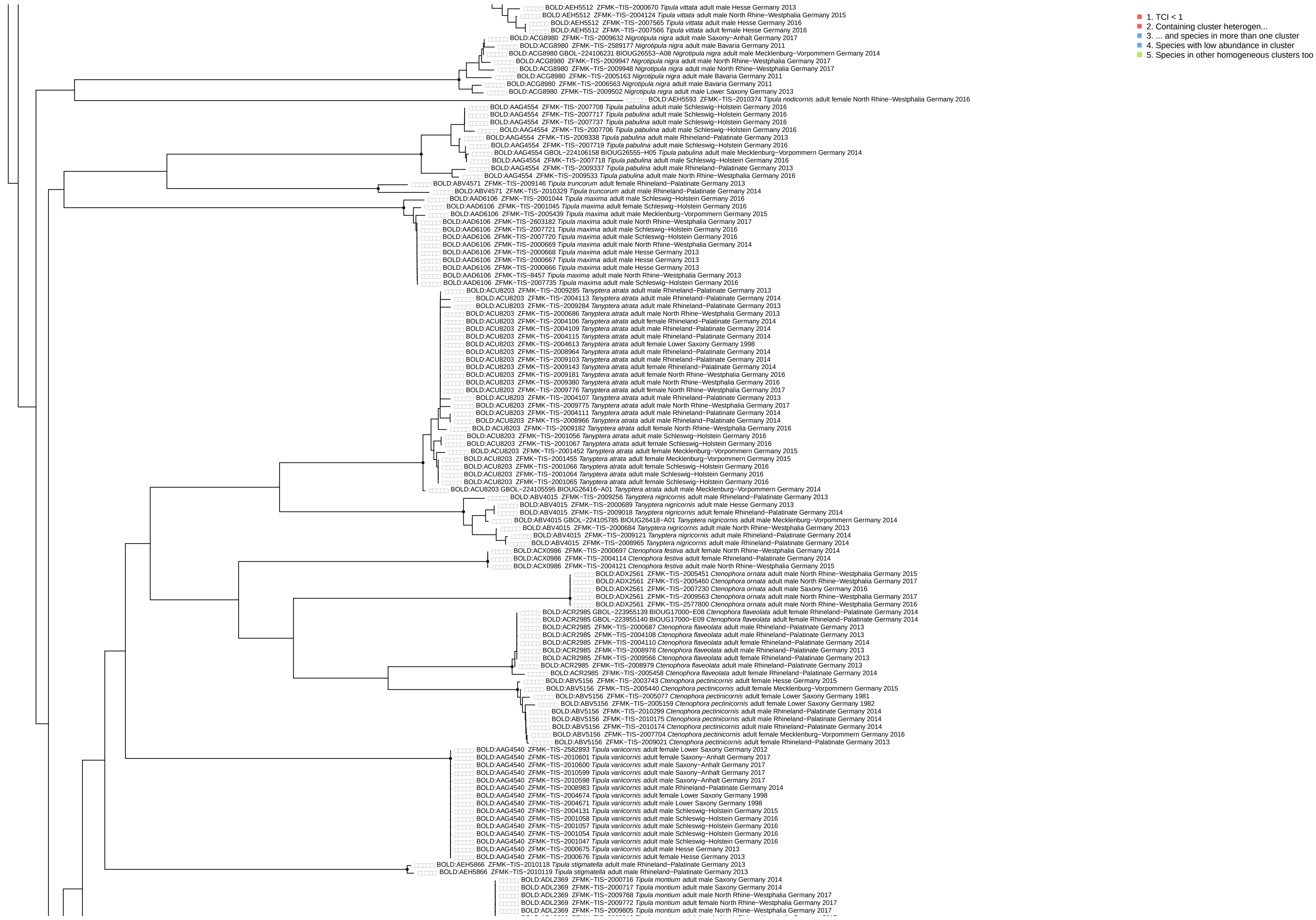

Fahldieck et al. 2024: Figure S3 (824_BINs), A priori determined species: 76, Barcode cluster: 74, Threshold: 0.0155

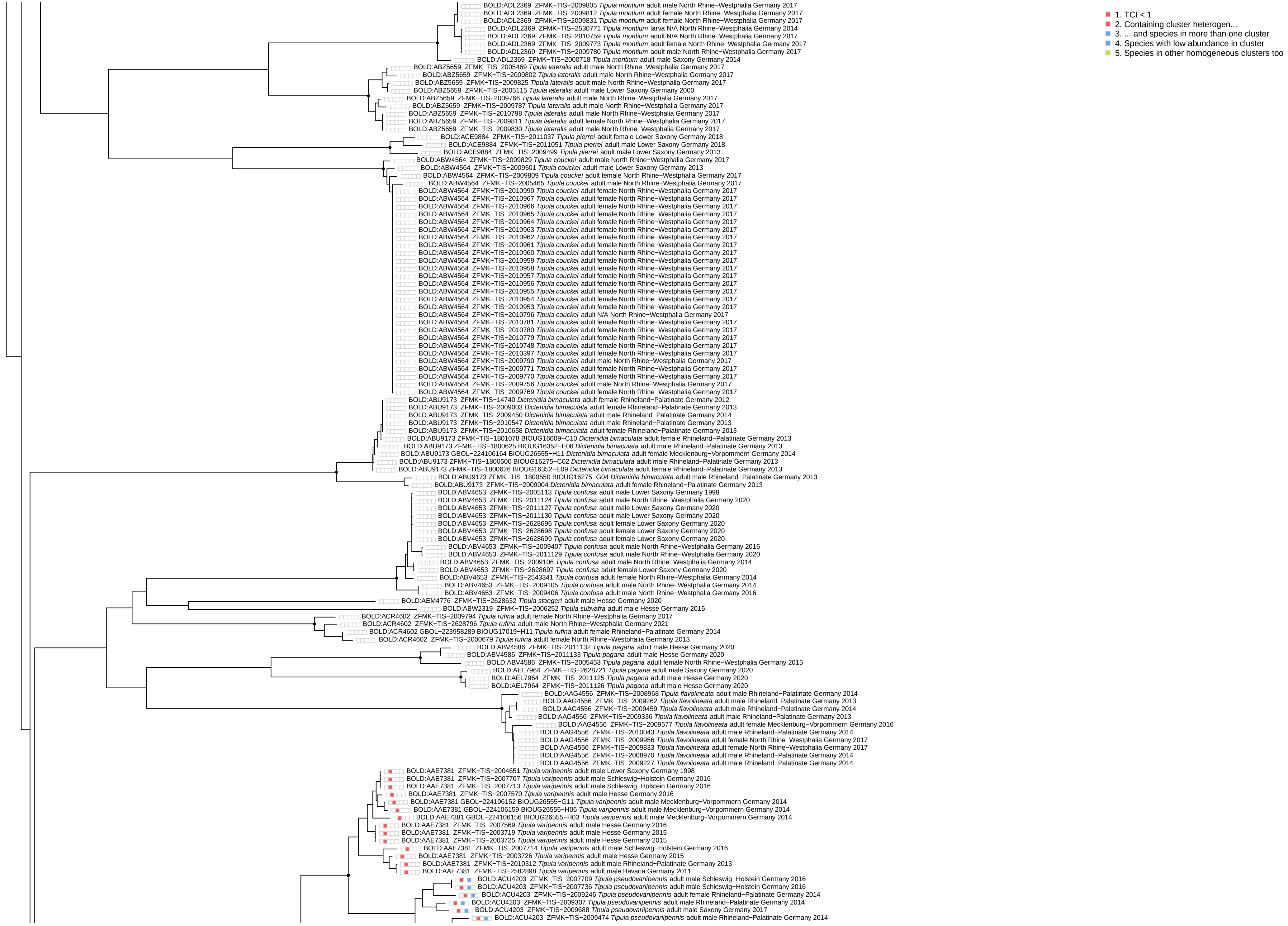

Fahldieck et al. 2024: Figure S3 (824_BINs), A priori determined species: 76, Barcode cluster: 74, Threshold: 0.0155

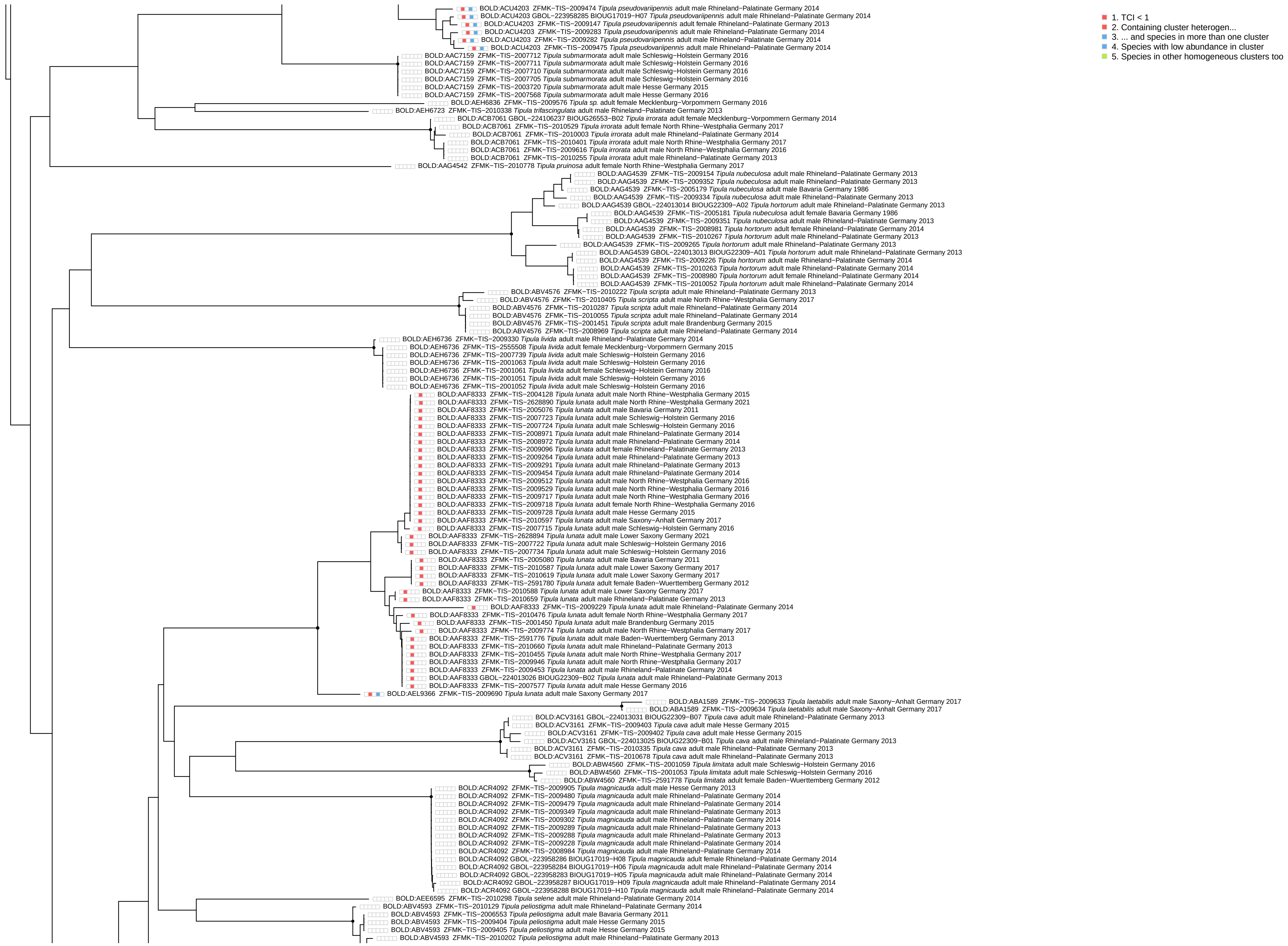

Fahldieck et al. 2024: Figure S3 (824_BINs), A priori determined species: 76, Barcode cluster: 74, Threshold: 0.0155

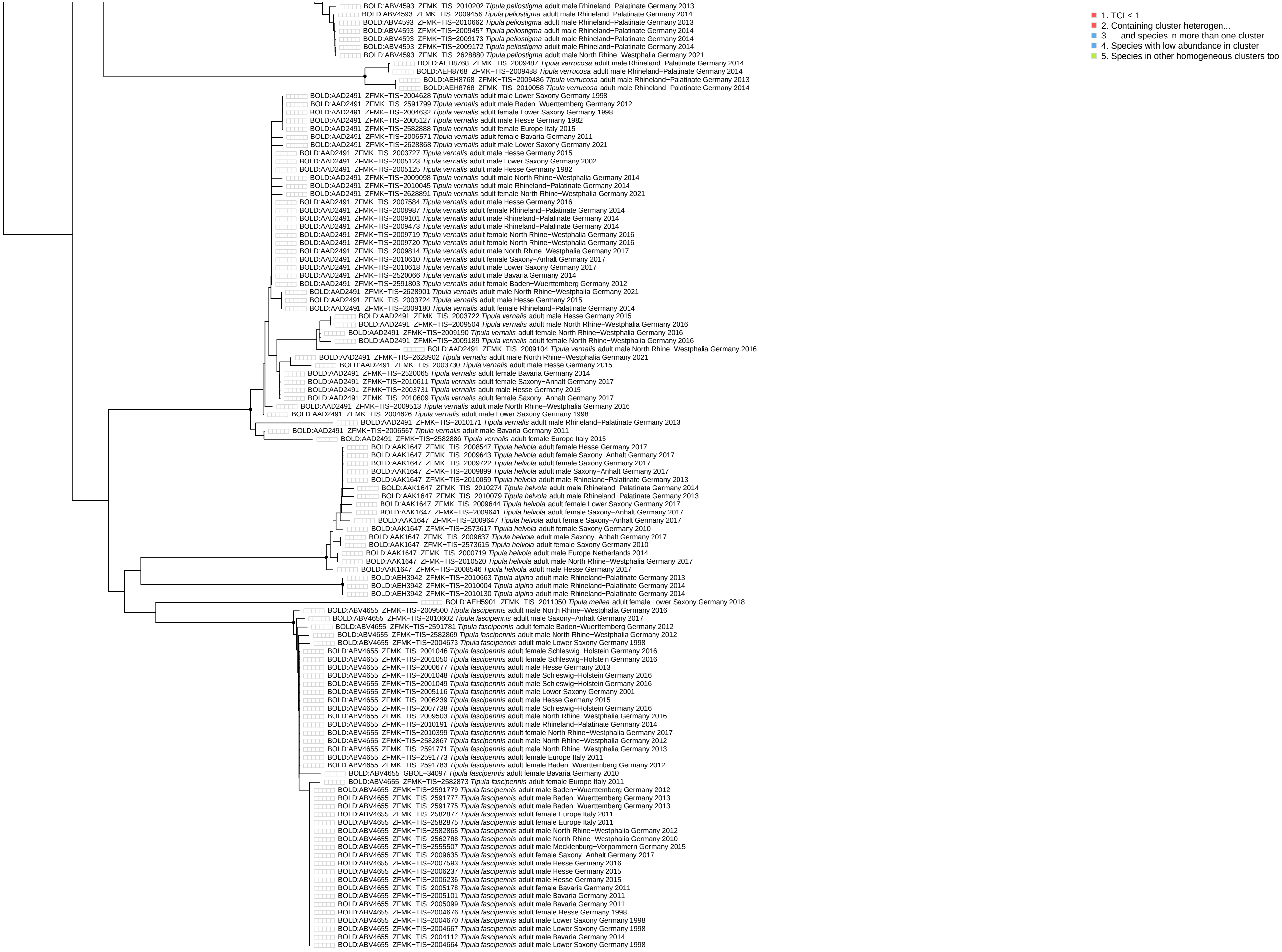

Fahldieck et al. 2024: Figure S3 (824_BINs), A priori determined species: 76, Barcode cluster: 74, Threshold: 0.0155
