# Supplementary material for: A DNA barcode reference library for the Tipulidae (Insecta, Diptera) of Germany
Source: Biodivers Data J. 2024 Sep 24;12:e127190. doi: 10.3897/BDJ.12.e127190 (PMC11445608; doi:10.3897/BDJ.12.e127190)
Supplement: Supplementary material 6 — Figure S4 [file bdj-12-e127190-s006.pdf]

Figure S4. Combined TaxCI tree of the 824 specimens of the GBOL Tipulidae and of the 696 specimens of the European Tipulidae scrutinized by their Barcode Index Numbers (BINs).

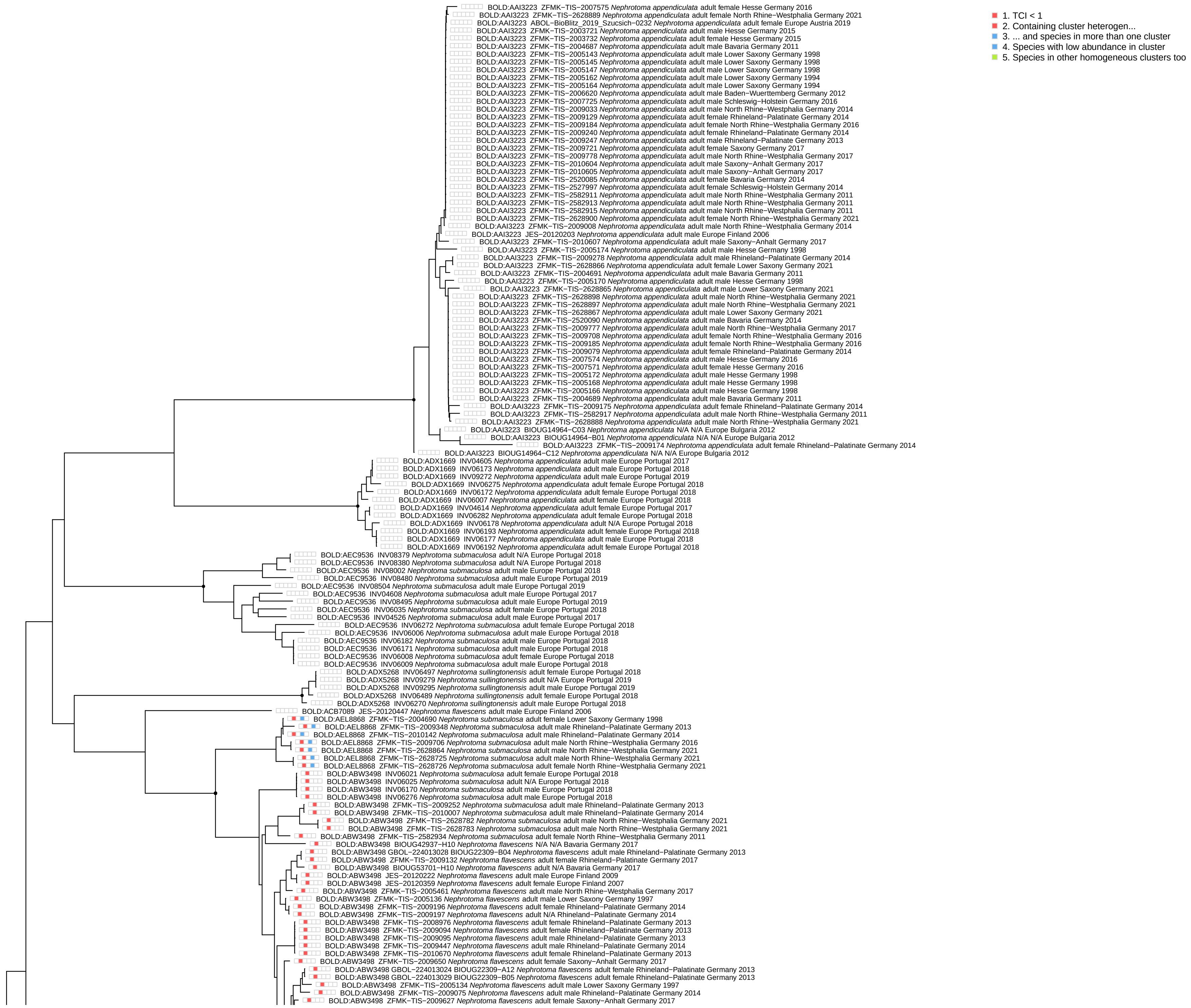

Fahldieck et al. 2024: Figure S4 (1520_BINs), A priori determined species: 160, Barcode cluster: 151, Threshold: 0.0247

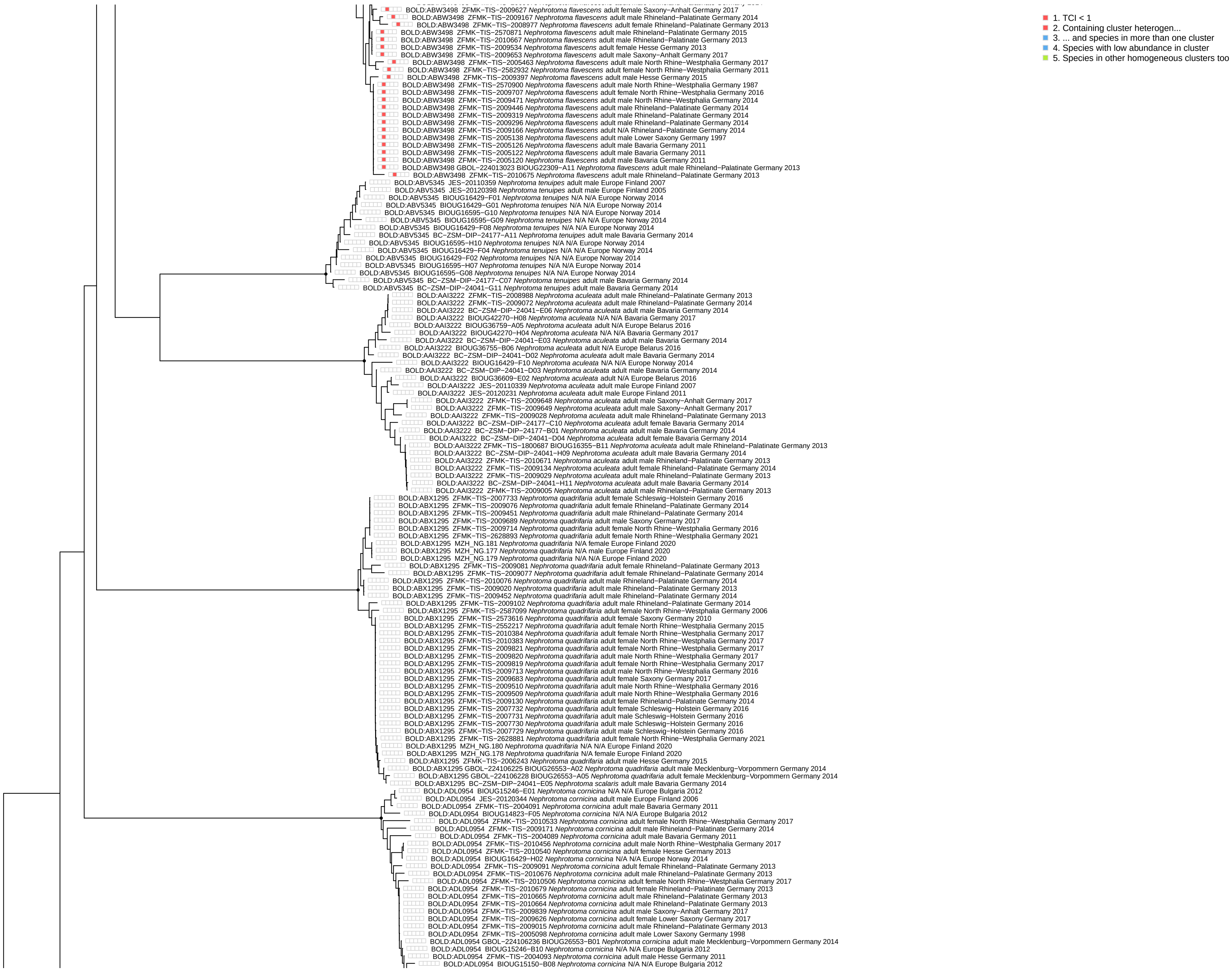

Fahldieck et al. 2024: Figure S4 (1520_BINs), A priori determined species: 160, Barcode cluster: 151, Threshold: 0.0247

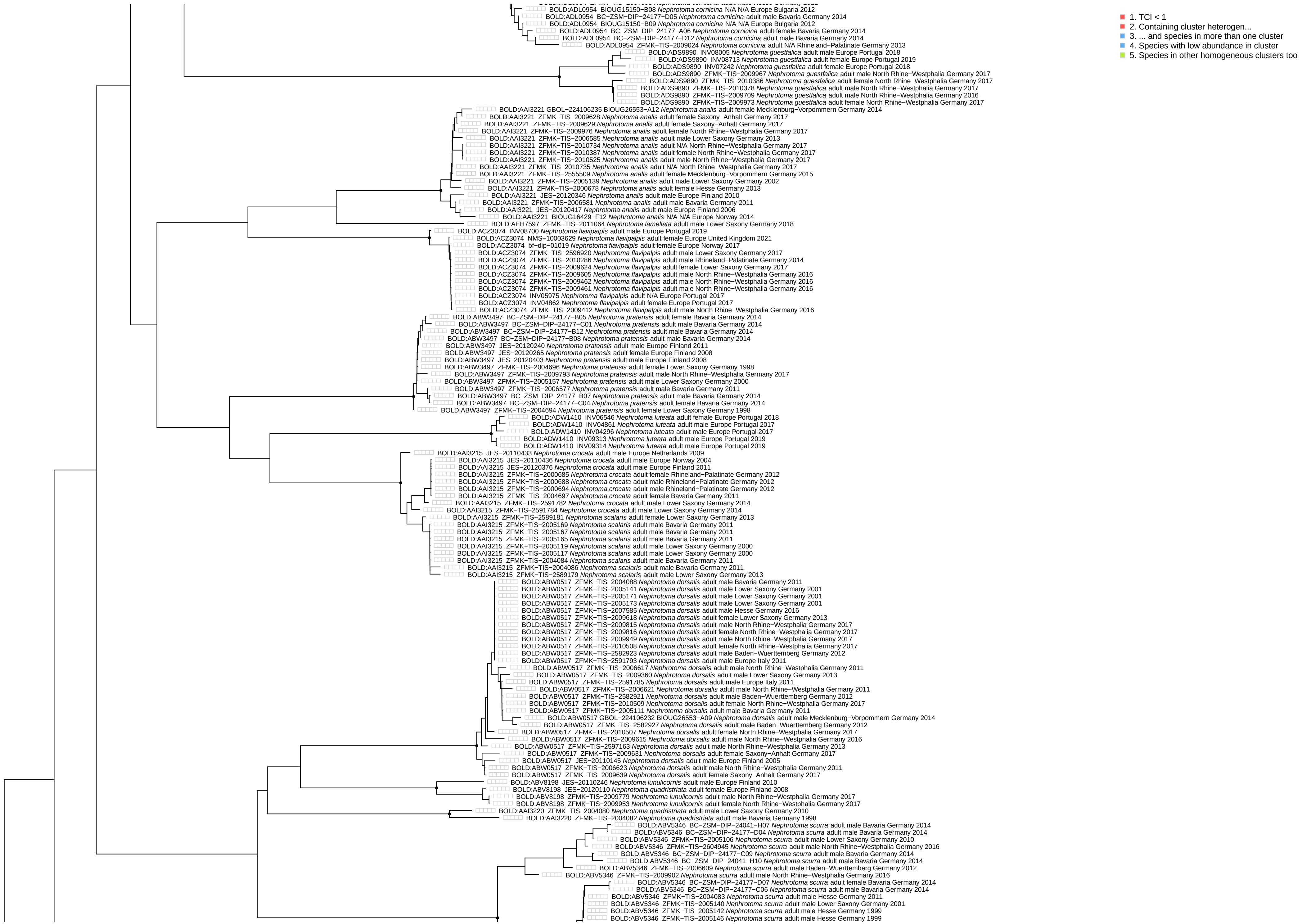

Fahldieck et al. 2024: Figure S4 (1520_BINs), A priori determined species: 160, Barcode cluster: 151, Threshold: 0.0247

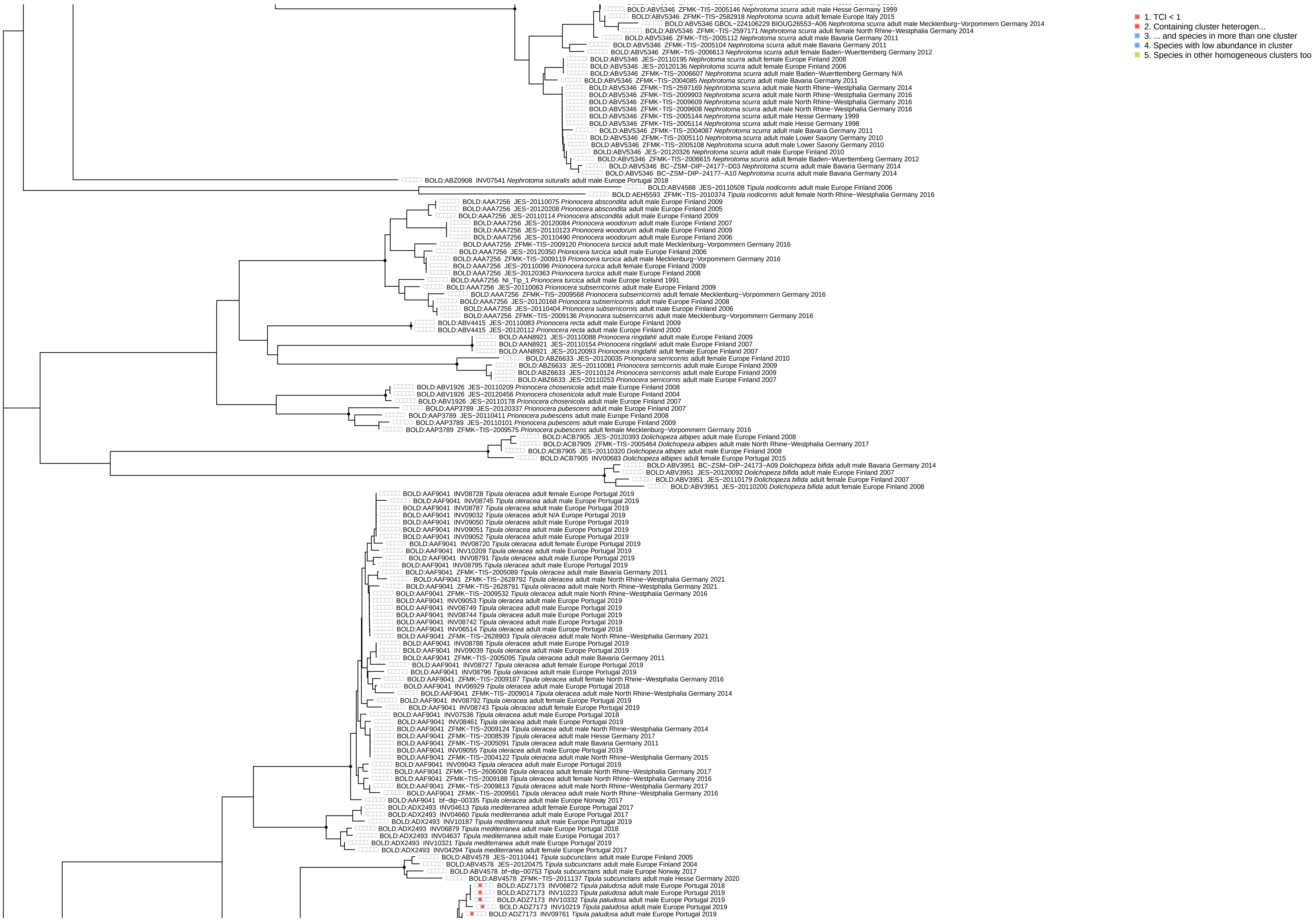

Fahldieck et al. 2024: Figure S4 (1520_BINs), A priori determined species: 160, Barcode cluster: 151, Threshold: 0.0247

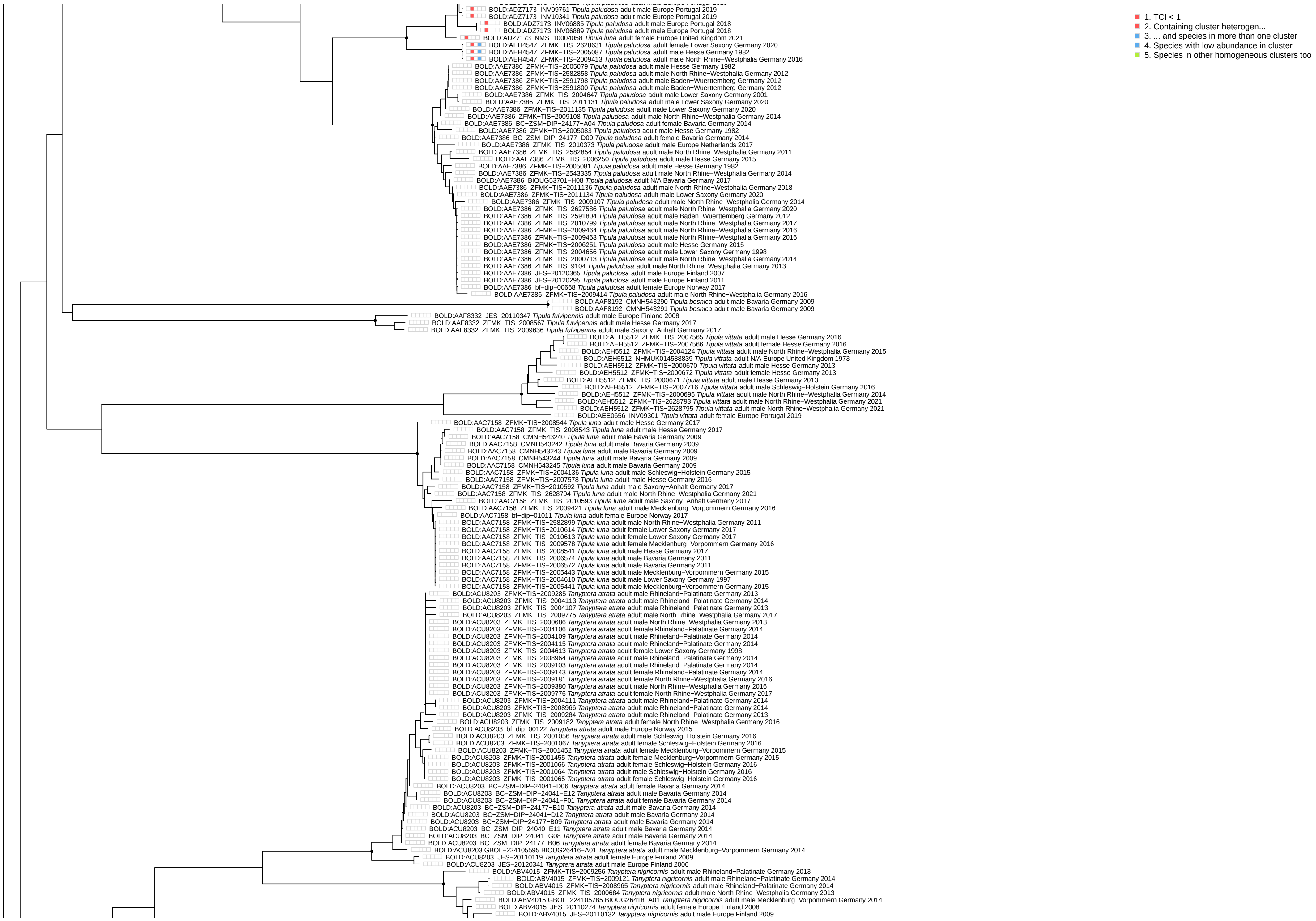

Fahldieck et al. 2024: Figure S4 (1520_BINs), A priori determined species: 160, Barcode cluster: 151, Threshold: 0.0247

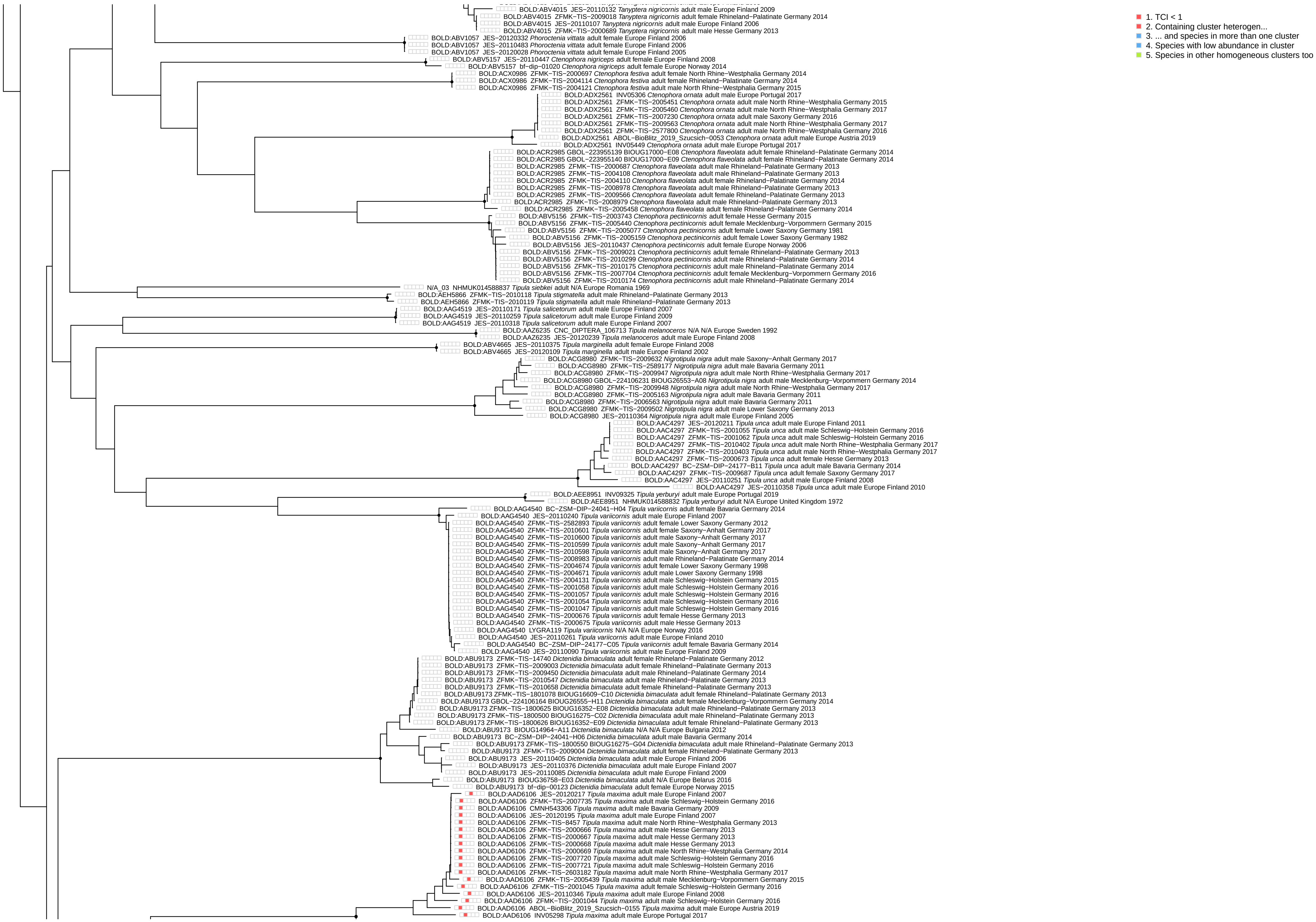

Fahldieck et al. 2024: Figure S4 (1520_BINS), A priori determined species: 160, Barcode cluster: 151, Threshold: 0.0247

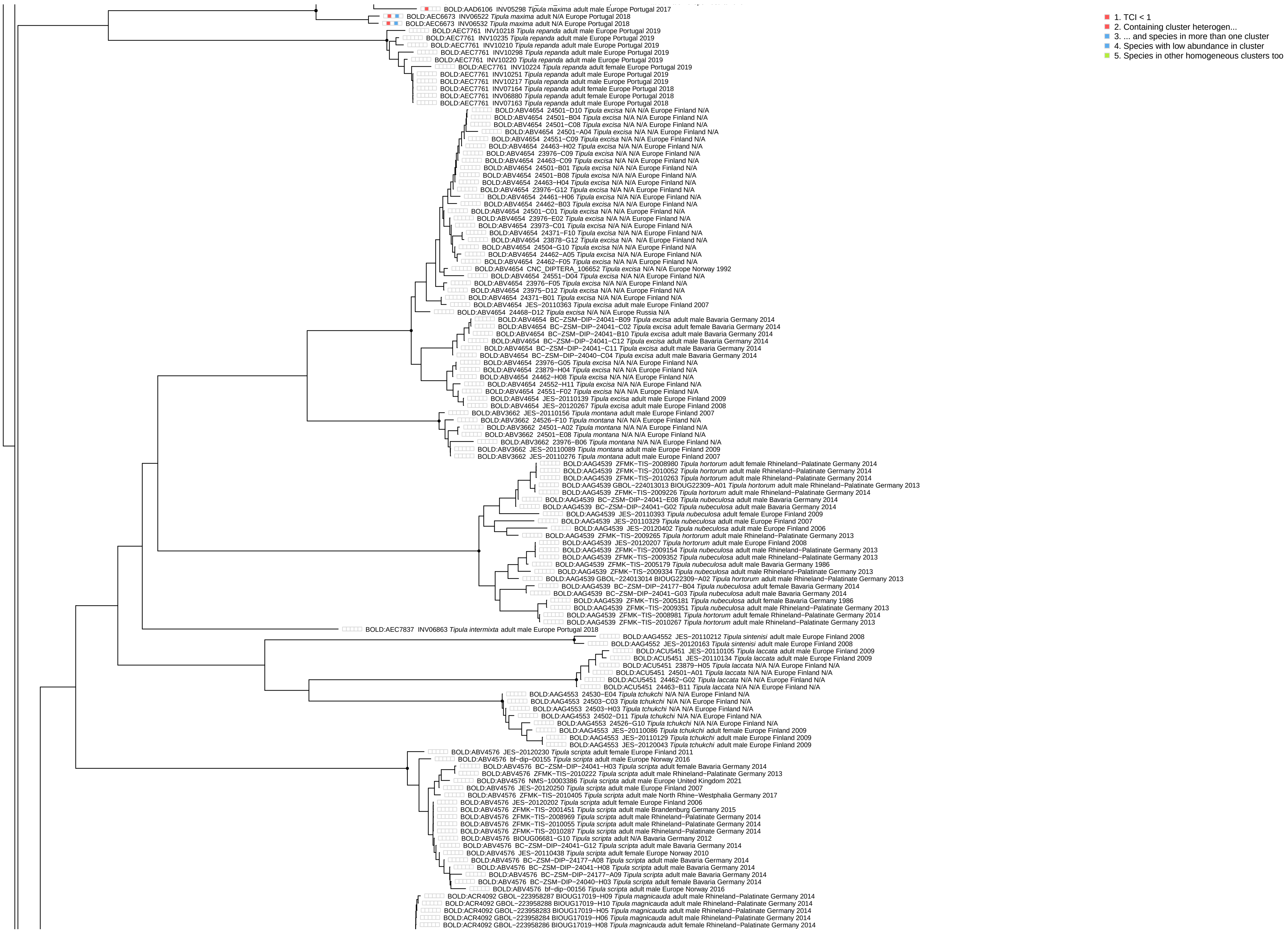

Fahldieck et al. 2024: Figure S4 (1520_BINs), A priori determined species: 160, Barcode cluster: 151, Threshold: 0.0247

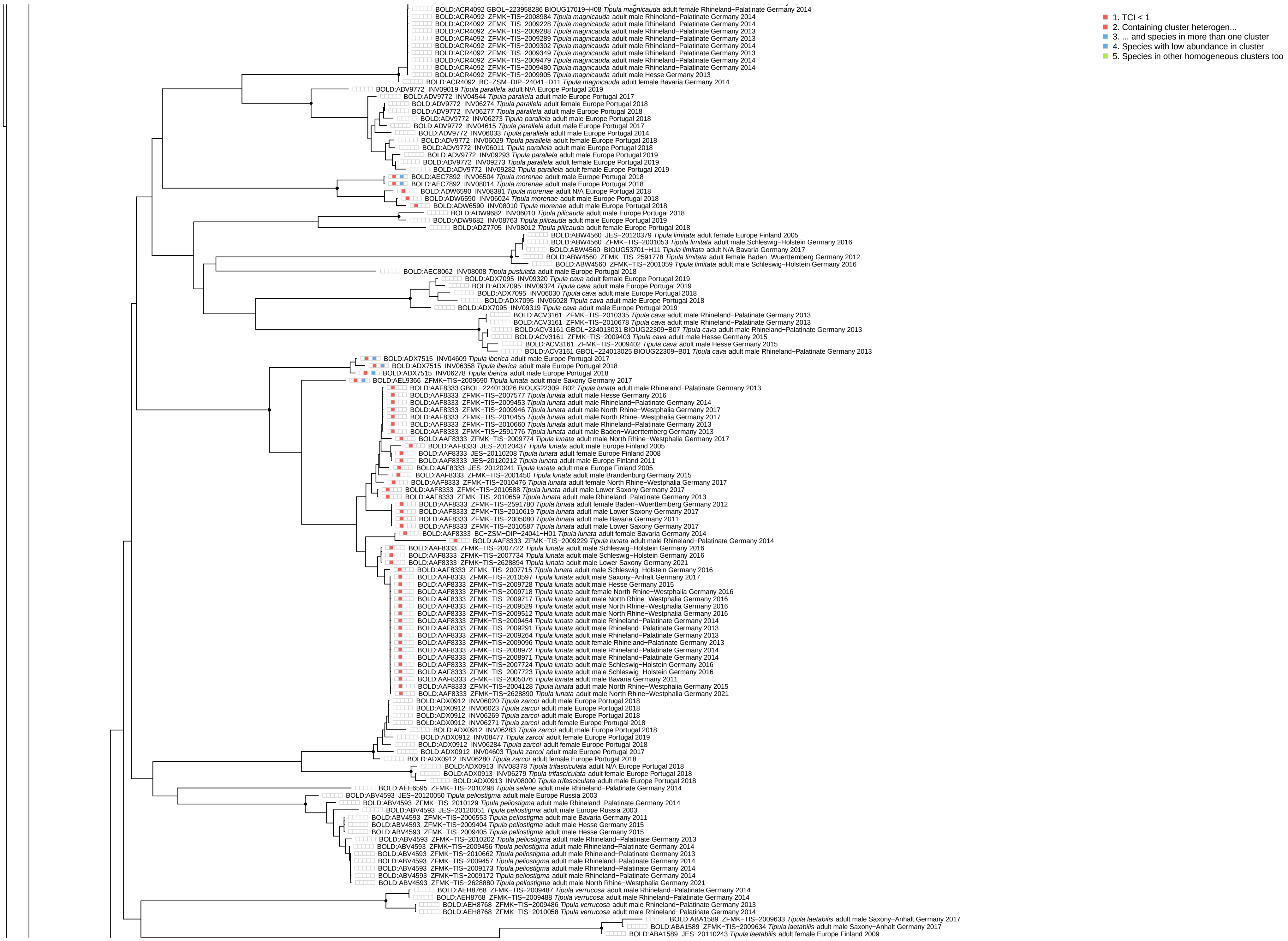

Fahldieck et al. 2024: Figure S4 (1520_BINs), A priori determined species: 160, Barcode cluster: 151, Threshold: 0.0247

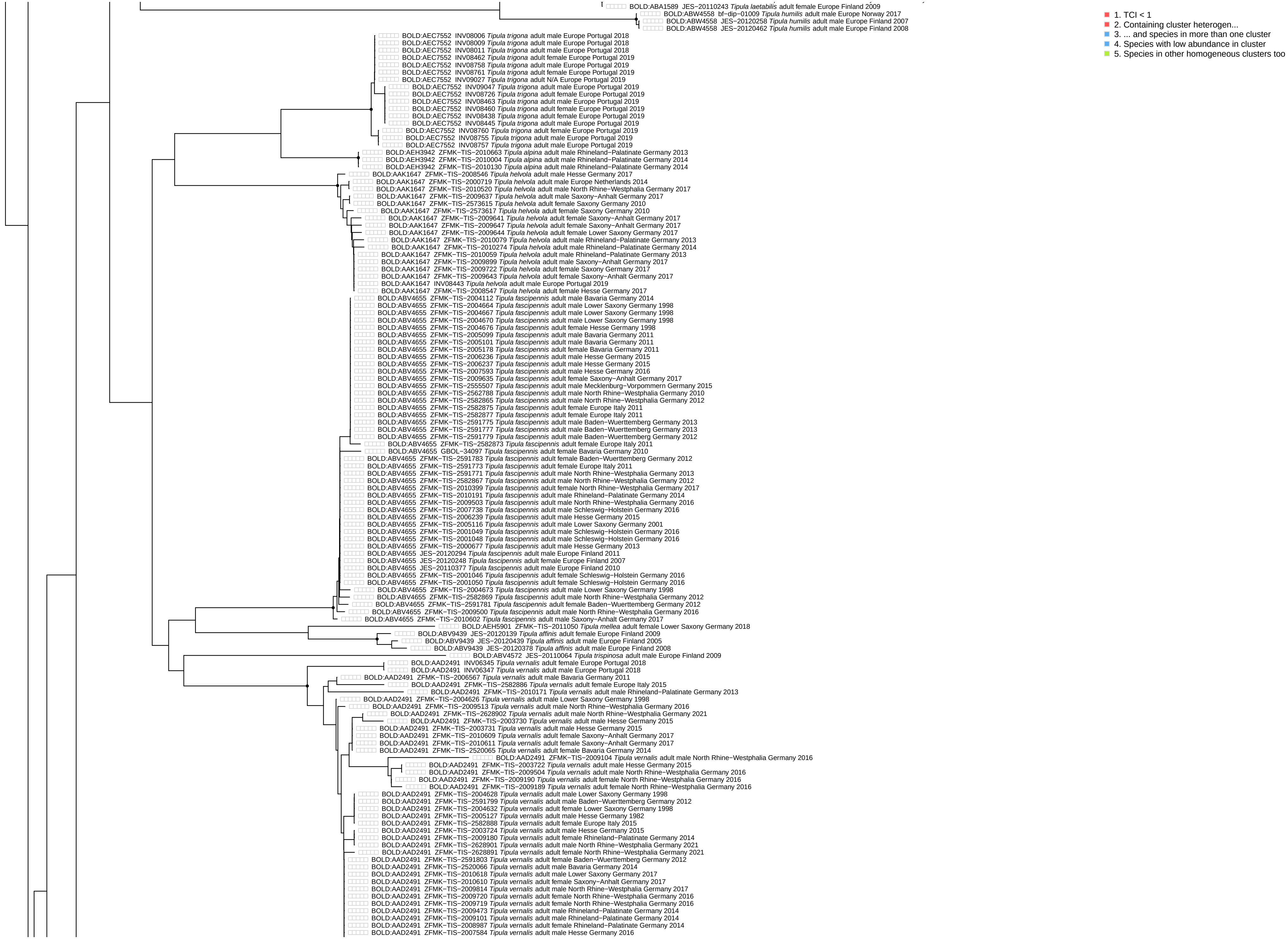

Fahldieck et al. 2024: Figure S4 (1520_BINs), A priori determined species: 160, Barcode cluster: 151, Threshold: 0.0247

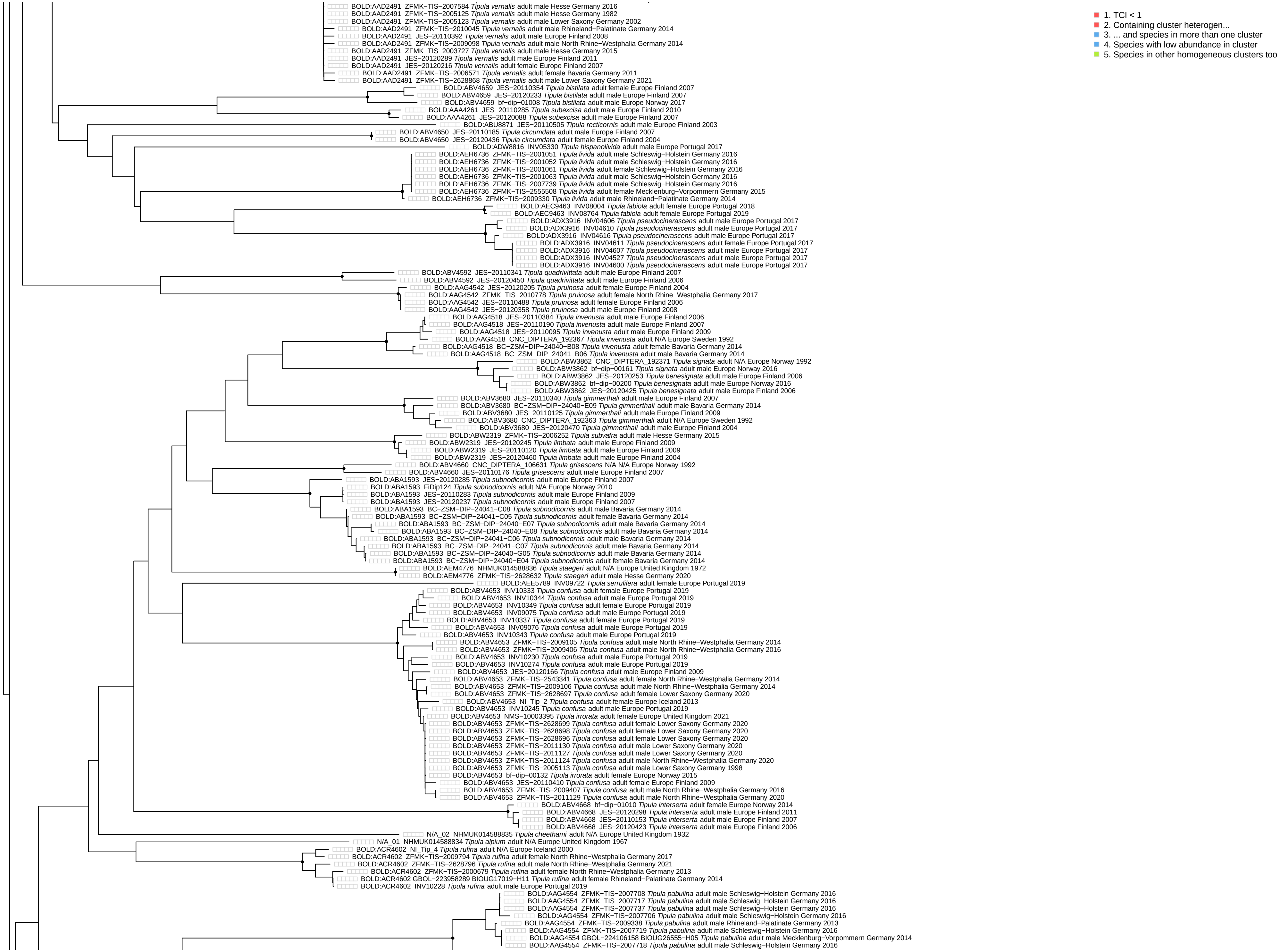

Fahldieck et al. 2024: Figure S4 (1520_BINs), A priori determined species: 160, Barcode cluster: 151, Threshold: 0.0247

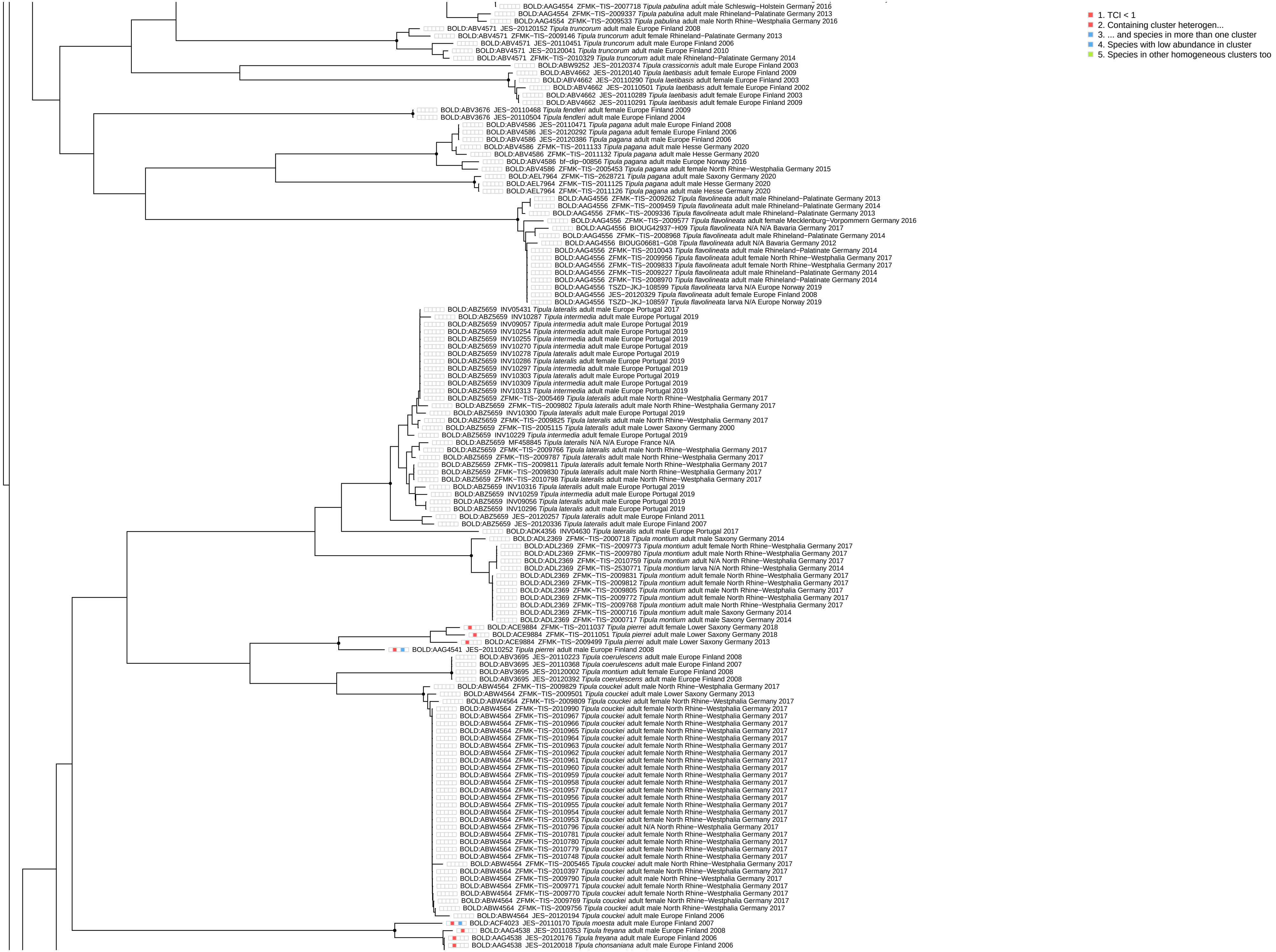

Fahldieck et al. 2024: Figure S4 (1520_BINs), A priori determined species: 160, Barcode cluster: 151, Threshold: 0.0247

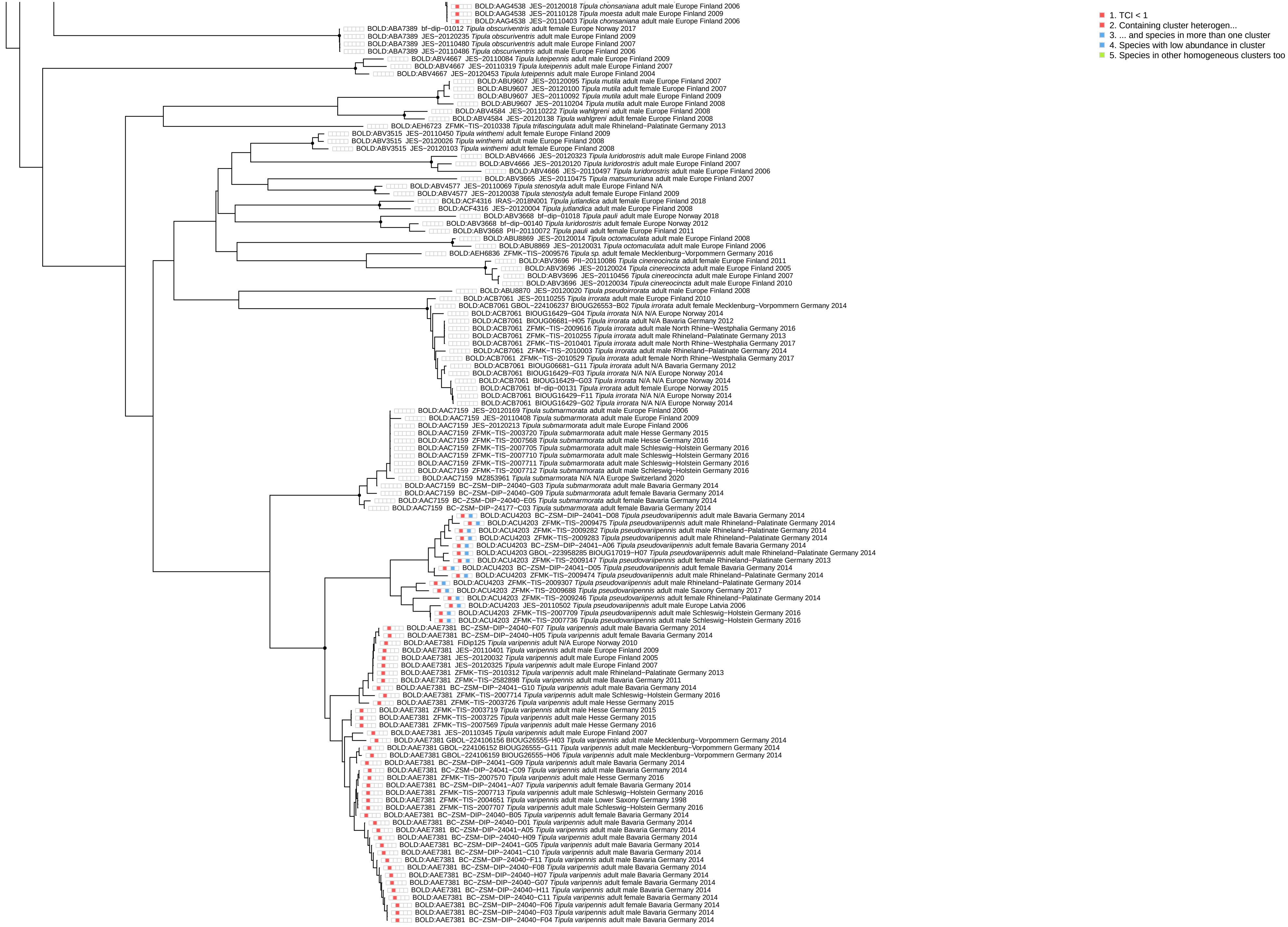

Fahldieck et al. 2024: Figure S4 (1520_BINs), A priori determined species: 160, Barcode cluster: 151, Threshold: 0.0247
