# Supplementary material for: A DNA barcode reference library for the Tipulidae (Insecta, Diptera) of Germany
Source: Biodivers Data J. 2024 Sep 24;12:e127190. doi: 10.3897/BDJ.12.e127190 (PMC11445608; doi:10.3897/BDJ.12.e127190)
Supplement: Supplementary material 7 — Figure S5 [file bdj-12-e127190-s007.pdf]

Figure S5. Combined TaxCI tree of the 824 specimens of the GBOL Tipulidae and of 688 specimens of the European Tipulidae, with eight clearly misidentified specimens removed.

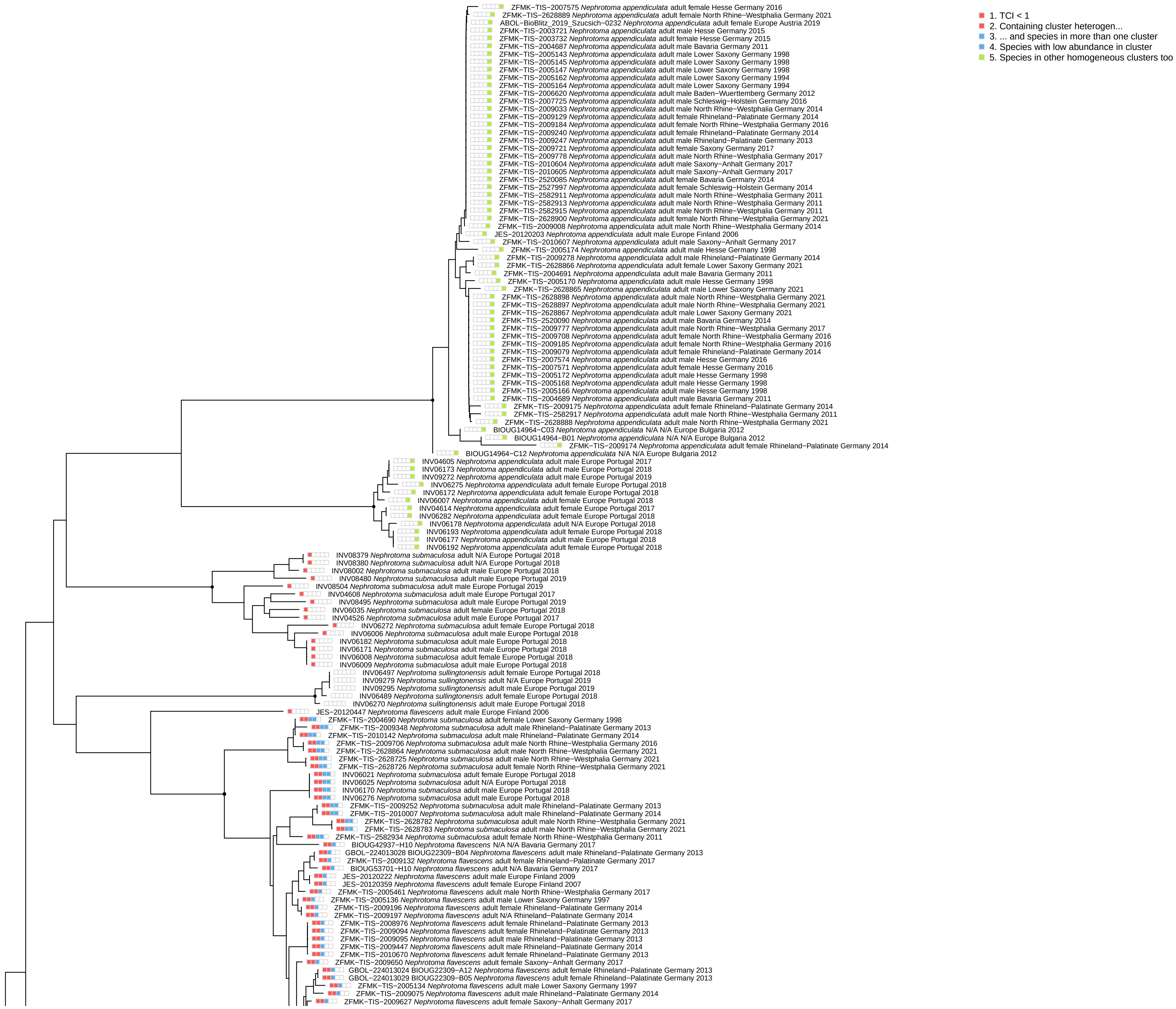

Fahldieck et al. 2024: Figure S5 (1512_wrong_deleted), A priori determined species: 154, Barcode cluster: 151, Threshold: 0.0247

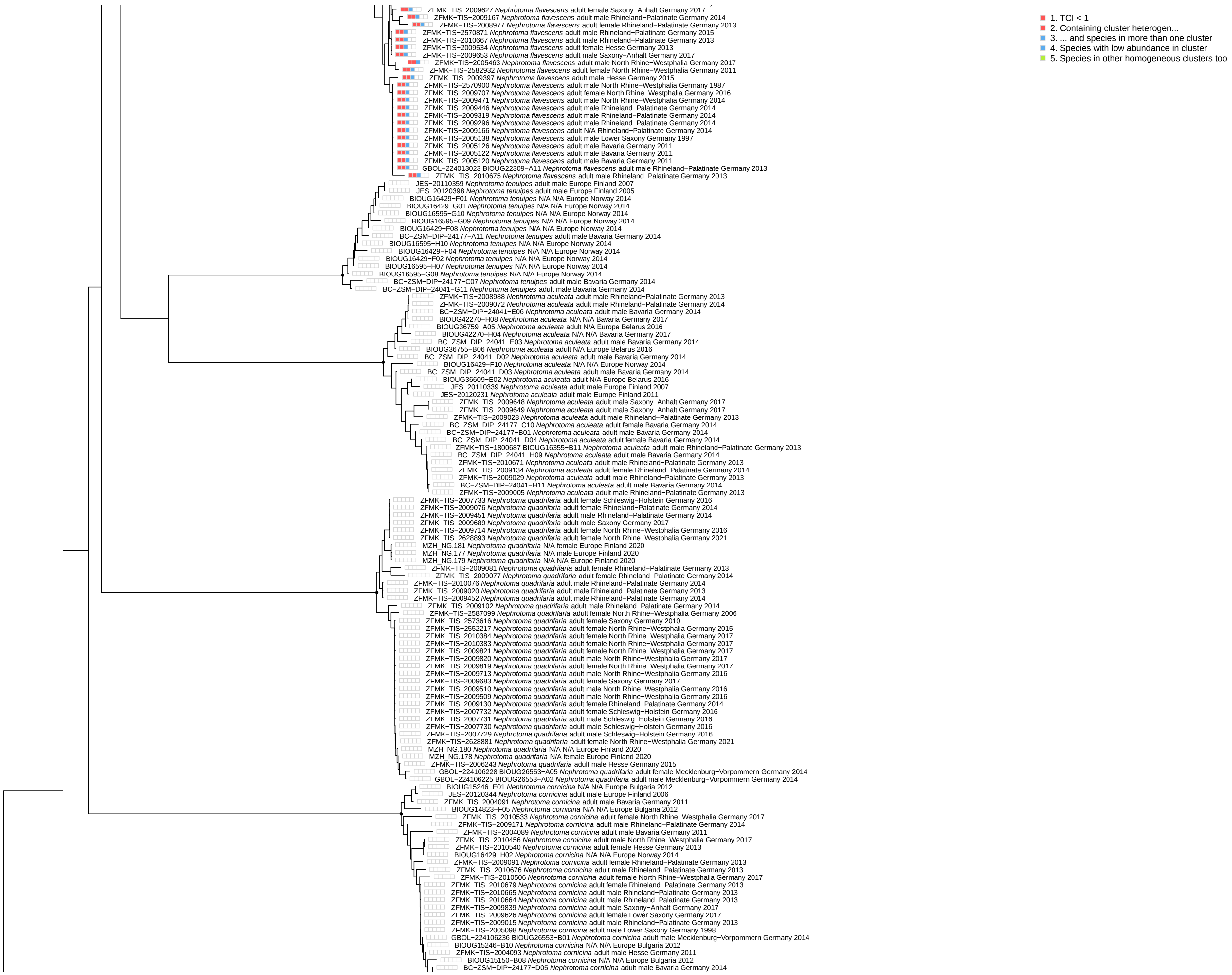

Fahldieck et al. 2024: Figure S5 (1512_wrong_deleted), A priori determined species: 154, Barcode cluster: 151, Threshold: 0.0247

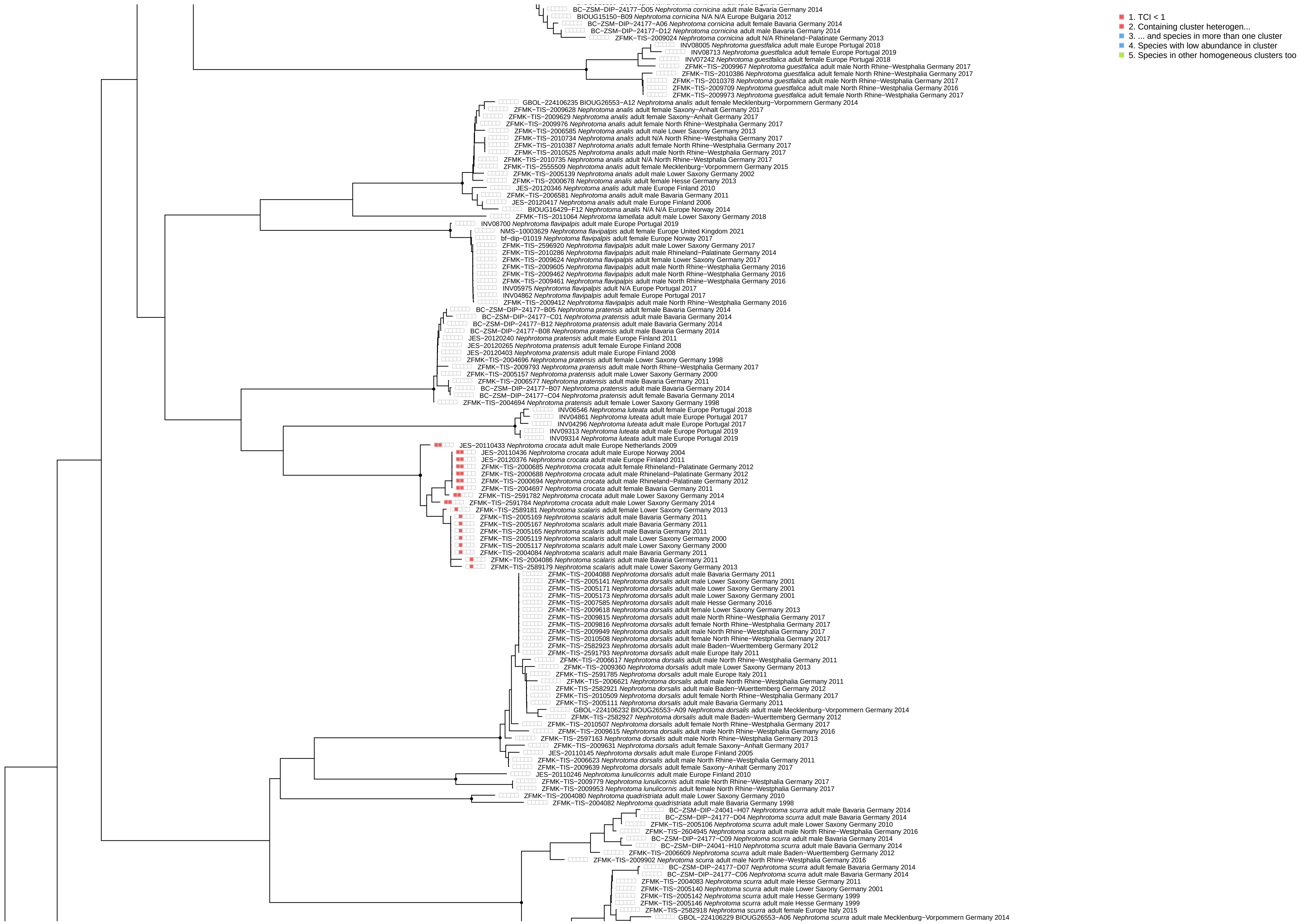

Fahldieck et al. 2024: Figure S5 (1512_wrong_deleted), A priori determined species: 154, Barcode cluster: 151, Threshold: 0.0247

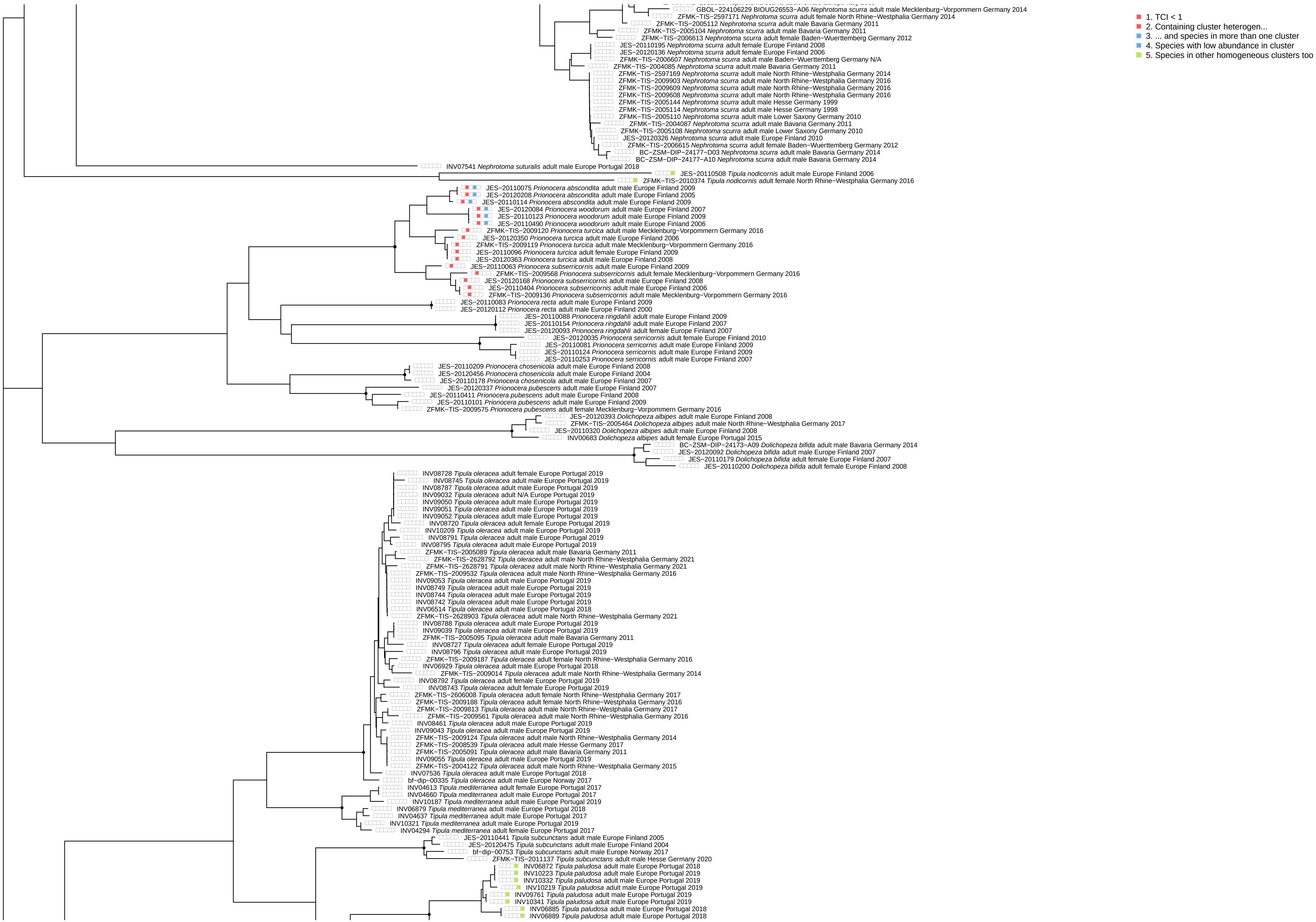

Fahldieck et al. 2024: Figure S5 (1512_wrong_deleted), A priori determined species: 154, Barcode cluster: 151, Threshold: 0.0247

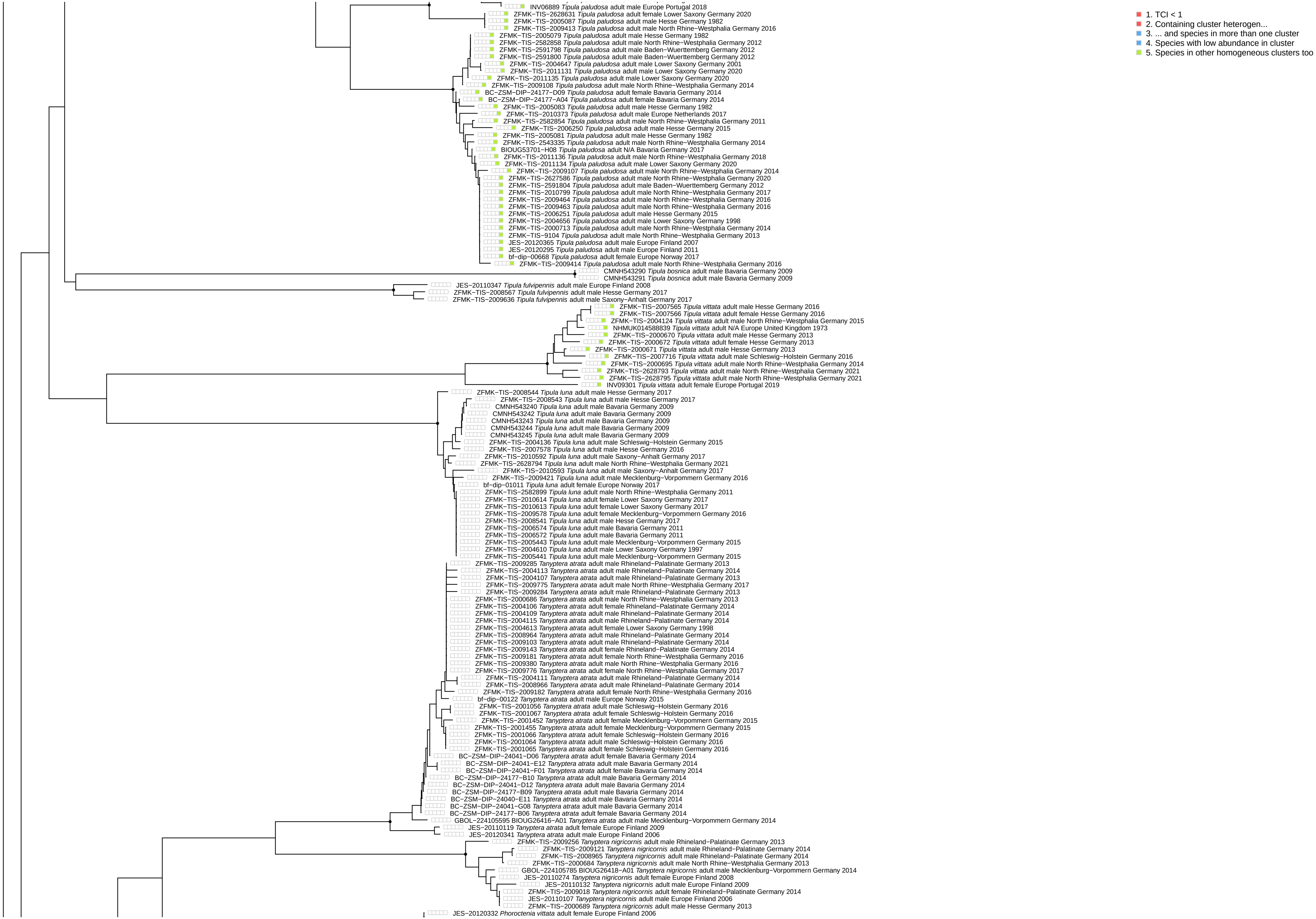

Fahldieck et al. 2024: Figure S5 (1512_wrong_deleted), A priori determined species: 154, Barcode cluster: 151, Threshold: 0.0247

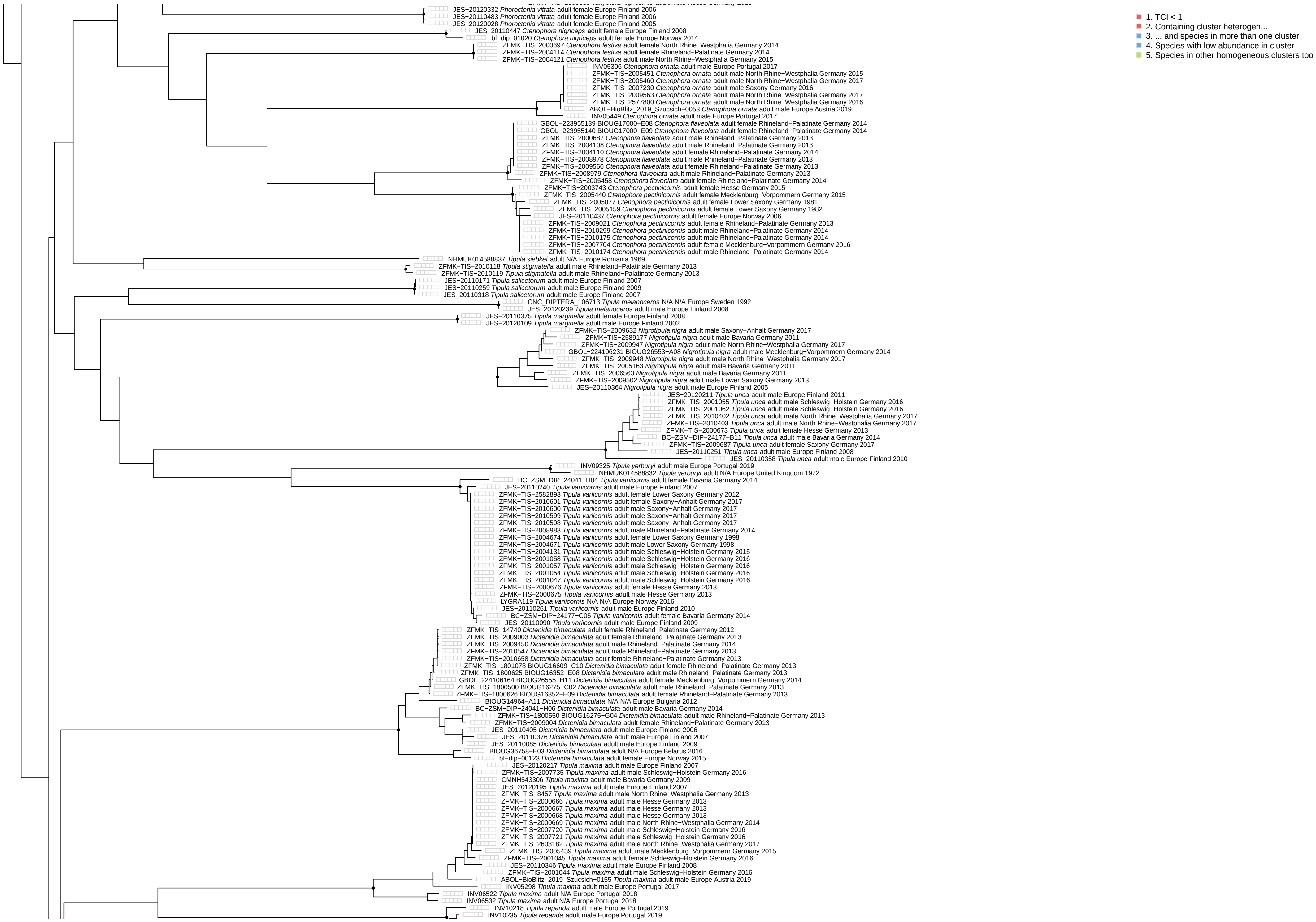

Fahldieck et al. 2024: Figure S5 (1512_wrong_deleted), A priori determined species: 154, Barcode cluster: 151, Threshold: 0.0247

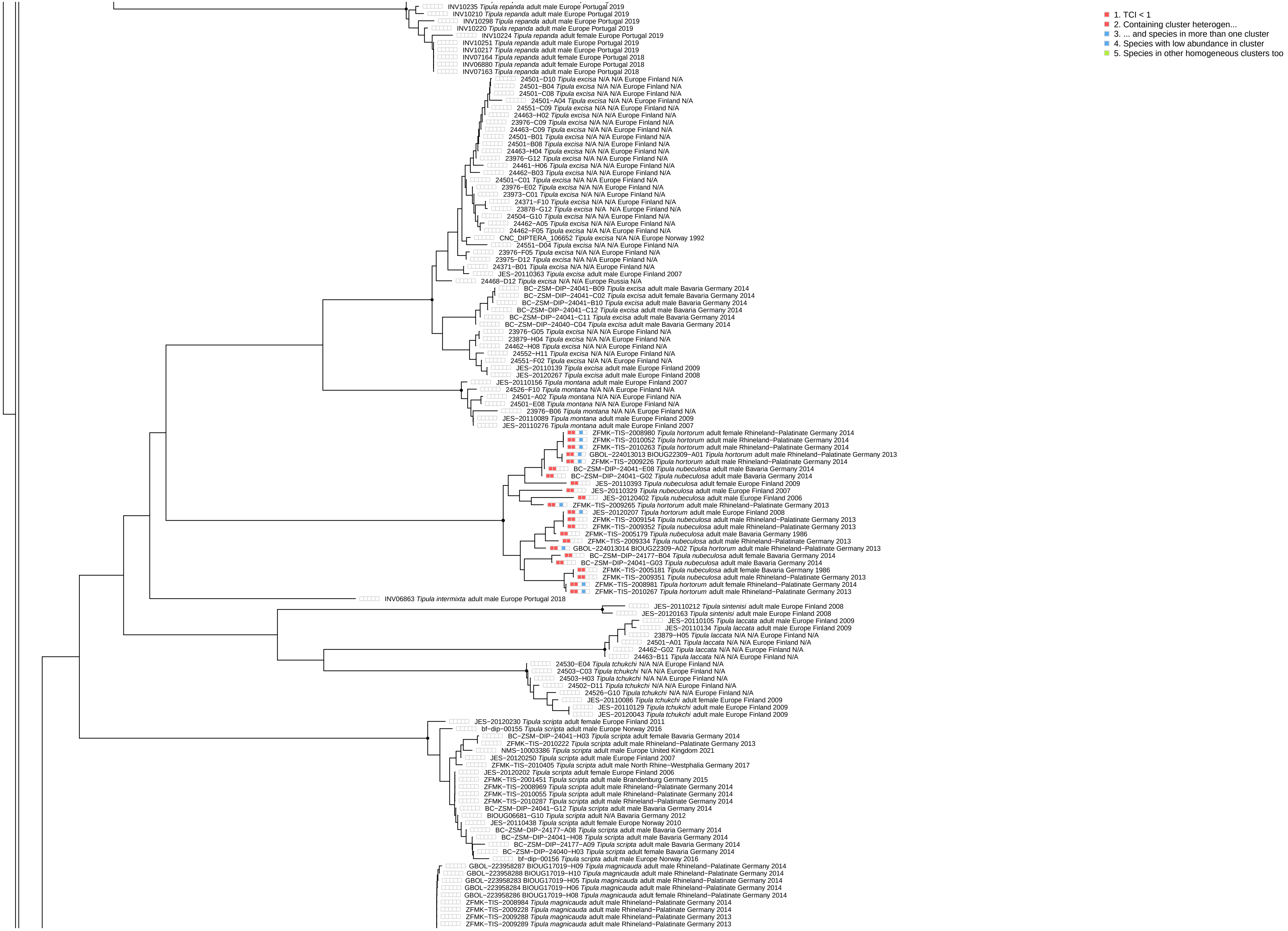

Fahldieck et al. 2024: Figure S5 (1512_wrong_deleted), A priori determined species: 154, Barcode cluster: 151, Threshold: 0.0247

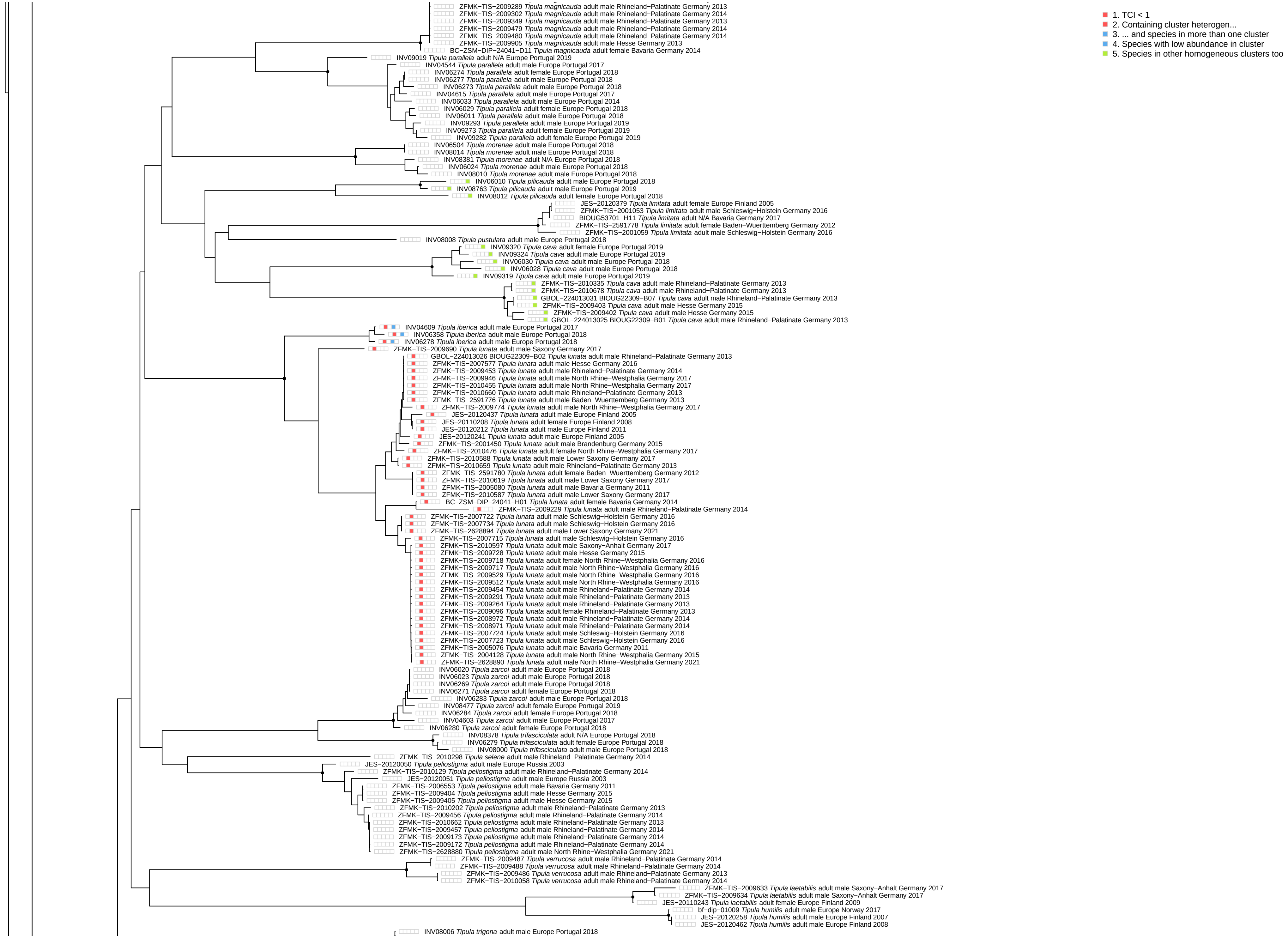

Fahldieck et al. 2024: Figure S5 (1512_wrong_deleted), A priori determined species: 154, Barcode cluster: 151, Threshold: 0.0247

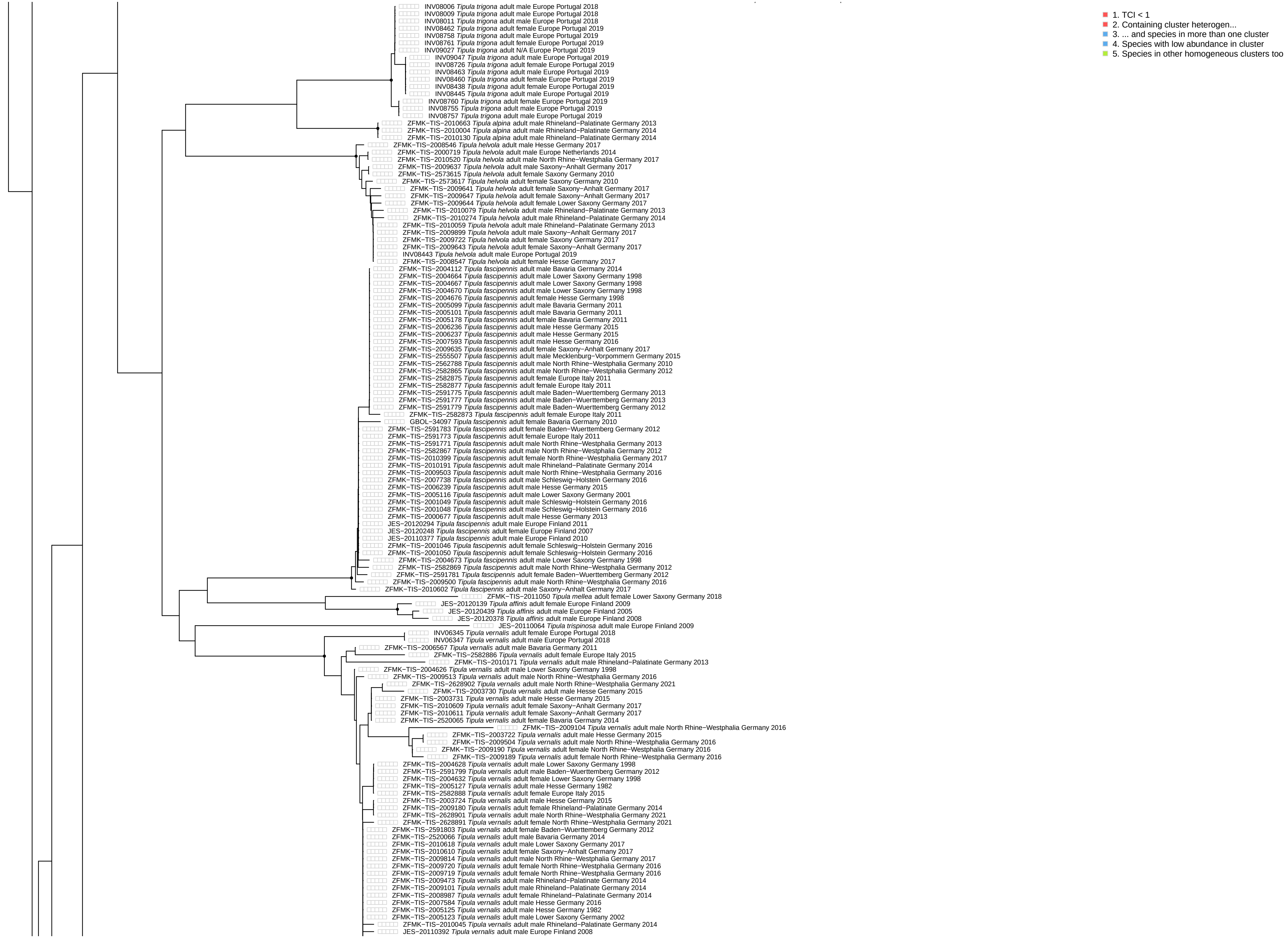

Fahldieck et al. 2024: Figure S5 (1512_wrong_deleted), A priori determined species: 154, Barcode cluster: 151, Threshold: 0.0247

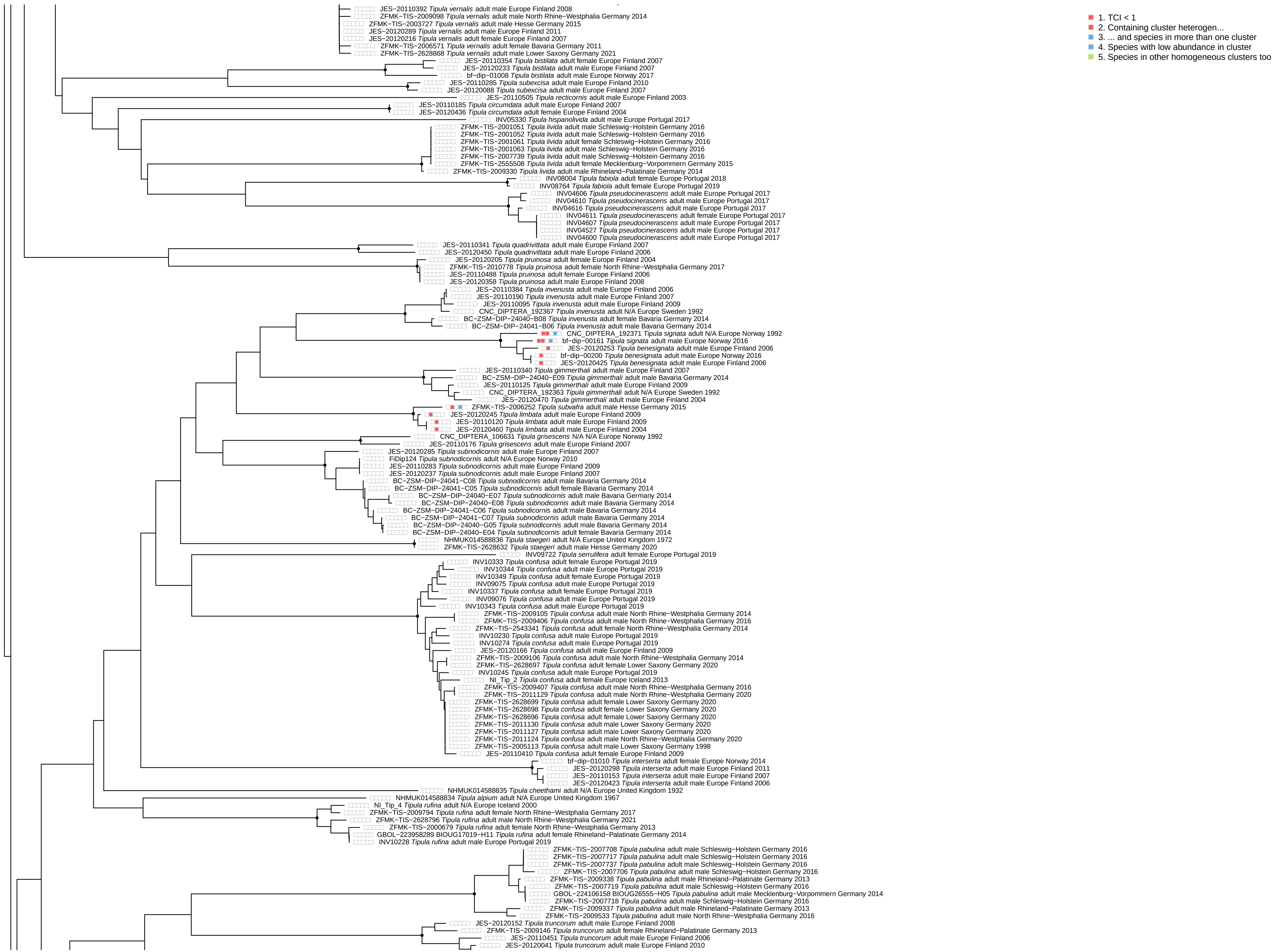

Fahldieck et al. 2024: Figure S5 (1512_wrong_deleted), A priori determined species: 154, Barcode cluster: 151, Threshold: 0.0247

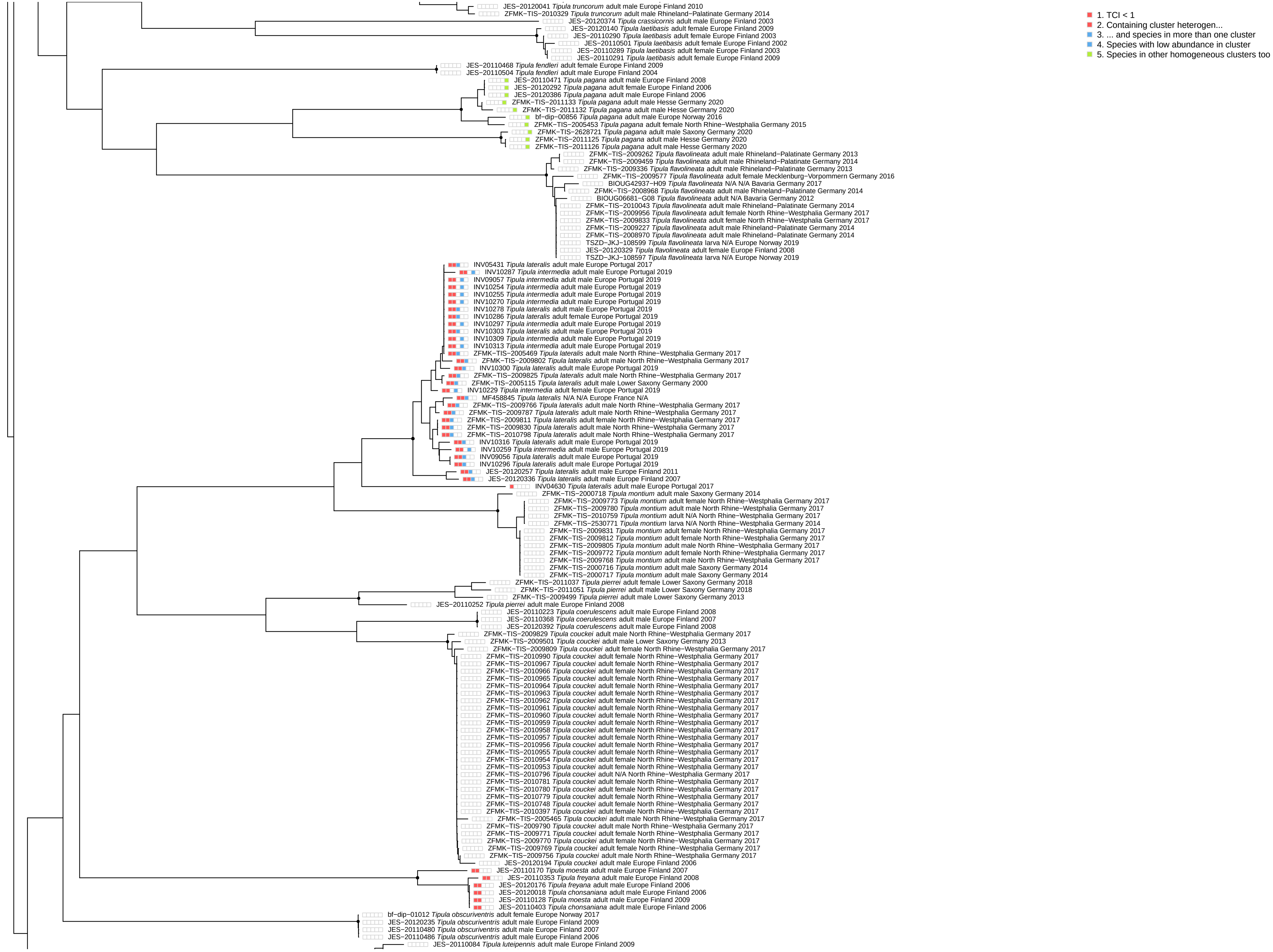

Fahldieck et al. 2024: Figure S5 (1512_wrong_deleted), A priori determined species: 154, Barcode cluster: 151, Threshold: 0.0247

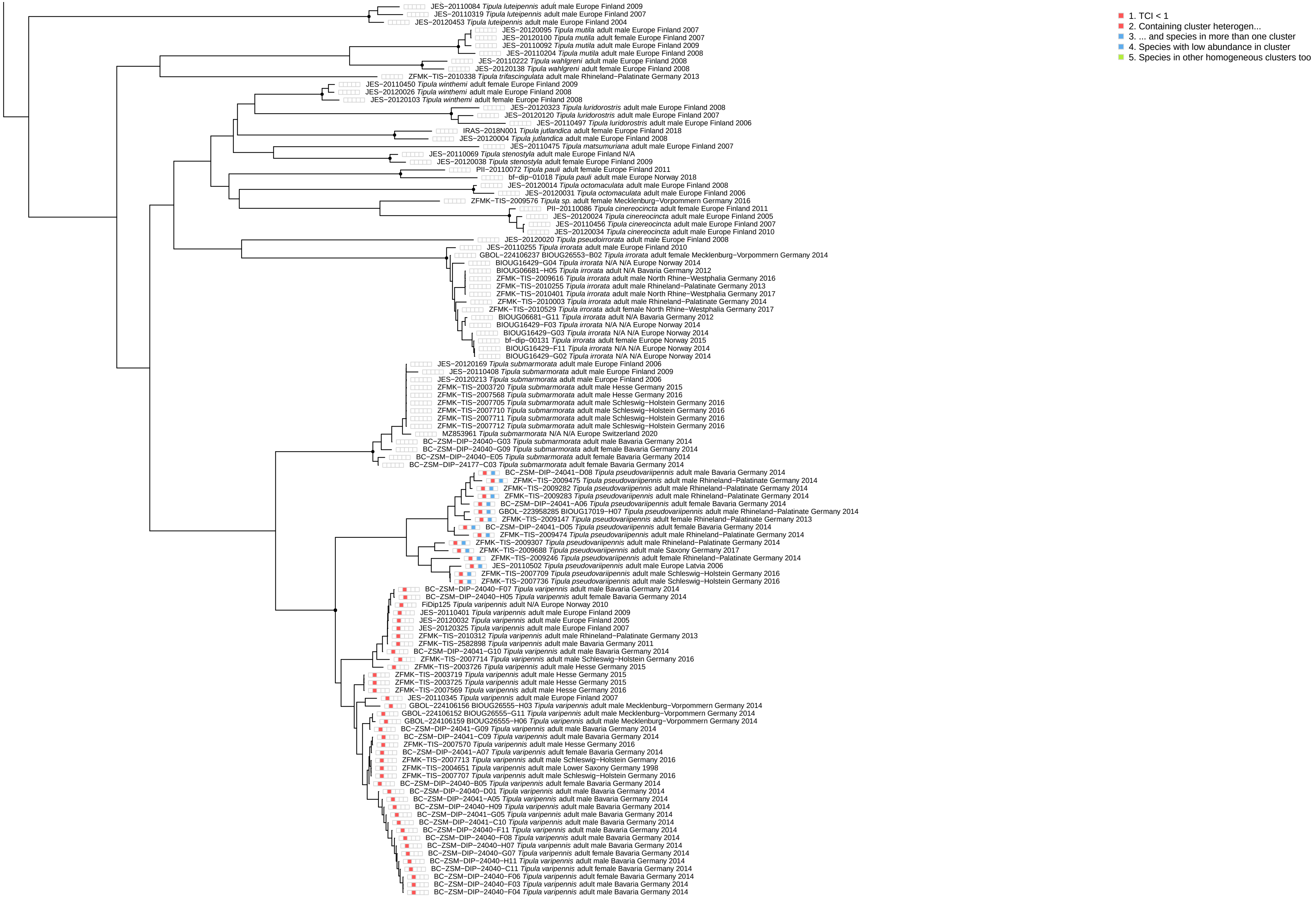

Fahldieck et al. 2024: Figure S5 (1512_wrong_deleted), A priori determined species: 154, Barcode cluster: 151, Threshold: 0.0247
