# Supplementary material for: A DNA barcode reference library for the Tipulidae (Insecta, Diptera) of Germany
Source: Biodivers Data J. 2024 Sep 24;12:e127190. doi: 10.3897/BDJ.12.e127190 (PMC11445608; doi:10.3897/BDJ.12.e127190)
Supplement: Supplementary material 10 — Appendix 1 [file bdj-12-e127190-s010.pdf]

Species-specific remarks for the GBOL Tipulidae

Ctenophora (*Cnemoncosis*) *festiva* Meigen, 1804

Three adult specimens from Germany of *Ctenophora* (*Cnemoncosis*) *festiva* Meigen, 1804 have been barcoded (1 male and 2 females). The barcodes form one unique cluster. All barcodes in this cluster are identical (no distance). The nearest neighboring cluster to the cluster of the barcodes of *C. festiva* is the one of the barcodes of *Ctenophora* (*Ctenophora*) *pectinicornis* (Linnaeus, 1758). The minimal p-distance between a barcode from the *C. festiva* and the *C. pectinicornis* cluster is 7.45%.

Ctenophora (*Ctenophora*) *flaveolata* (Fabricius, 1794)

Nine adult specimens from Germany of *Ctenophora* (*Ctenophora*) *flaveolata* (Fabricius, 1794) have been barcoded (4 males and 5 females). The minimal p-distance between barcodes in this cluster is 0.00% and the maximal p-distance is 0.31% (mean = 0.06%). The nearest neighboring cluster to the cluster of the barcodes of *C. flaveolata* is the one of the barcodes of *Ctenophora* (*Ctenophora*) *pectinicornis* (Linnaeus, 1758). The minimal p-distance between a barcode from the *C. flaveolata* and the *C. pectinicornis* cluster is 3.80%.

Ctenophora (*Cnemoncosis*) *ornata* Meigen & Wiedemann, 1818

Five male, adult specimens from Germany of *Ctenophora* (*Cnemoncosis*) *ornata* Meigen & Wiedemann, 1818 have been barcoded. The barcodes form one unique cluster. All barcodes in this cluster are identical (no distance). The nearest neighboring cluster to the cluster of the barcodes of *C. ornata* is the one of the barcodes of *Ctenophora* (*Ctenophora*) *pectinicornis* (Linnaeus, 1758). The minimal p-distance between a barcode from the *C. ornata* and the *C. pectinicornis* cluster is 6.99%.

Ctenophora (*Ctenophora*) *pectinicornis* (Linnaeus, 1758)

Nine adult specimens from Germany of *Ctenophora* (*Ctenophora*) *pectinicornis* (Linnaeus, 1758) have been barcoded (3 males and 6 females). The barcodes form one unique cluster. The minimal p-distance between barcodes in this cluster is 0.00% and the maximal p-distance is 0.30% (mean = 0.10%). The nearest neighboring cluster to the cluster of the barcodes of *C. pectinicornis* is the one of the barcodes of *Ctenophora* (*Ctenophora*) *flaveolata* (Fabricius, 1794). The minimal p-distance between a barcode from the *C. pectinicornis* and the *C. flaveolata* cluster is 3.80%.

Dictenidia bimaculata (Linnaeus, 1760)

12 adult specimens from Germany of *Dictenidia bimaculata* (Linnaeus, 1760) have been barcoded (5 males and 7 females).The barcodes form one unique cluster. The minimal p-distance between two barcodes in this cluster is 0.00% and the maximal p-distance is 1.77% (mean = 0.48%). The nearest neighboring cluster to the cluster of the barcodes of *D. bimaculata* is the one of the barcodes of *Tanyptera* (*Tanyptera*) *atrata* (Linnaeus, 1758). The minimal p-distance between a barcode from the *D. bimaculata* and the *T. atrata* cluster is 7.60%. mPTP and ASAP split the cluster of barcodes of *D. bimaculata* into two MOTUs (10 barcodes and 2 barcode).

Dolichopeza (*Dolichopeza*) *albipes* (Strom, 1768)

One male, adult specimen from Germany of *Dolichopeza* (*Dolichopeza*) *albipes* (Strom, 1768) has been barcoded (singleton). The nearest neighboring cluster to the barcode of *D. albipes* is the one of the barcodes of *Prionocera subserricornis* (Zetterstedt, 1851). The minimal p-distance between the barcode of *D. bimaculata* and a barcode from the *P. subserricornis* cluster is 10.03%.

Nephrotoma aculeata (Loew, 1871)

10 adult specimens from Germany of *Nephrotoma aculeata* (Loew, 1871) have been barcoded (9 males and 1 female). The barcodes form one unique cluster. The minimal p-distance between two barcodes in this cluster is 0.00% and the maximal p-distance is 0.52% (mean = 0.28%). The nearest neighboring cluster to the cluster of the barcodes of *N. aculeata* is the one of the barcodes of *Nephrotoma quadrifaria* (Meigen, 1804). The minimal p-distance between a barcode from the *N. aculeata* and the *N. quadrifaria* cluster is 7.02%.

Nephrotoma analis (Schummel, 1833)

13 adult specimens from Germany of *Nephrotoma analis* (Schummel, 1833) have been barcoded (4 males, 7 females and 2 n/a). The barcodes form one unique cluster. The minimal p-distance between two barcodes in this cluster is 0.00% and the maximal p-distance is 0.30% (mean = 0.15%). The nearest neighboring cluster to the cluster of the barcodes of *N. analis* is the one of the barcodes of *Nephrotoma lamellata* (Riedel, 1910). The minimal p-distance between a barcode from the *N. analis* and the *N. lamellata* cluster is 3.55%.

Nephrotoma appendiculata (Pierre, 1919)

53 adult specimens from Germany of *Nephrotoma appendiculata* (Pierre, 1919) have been barcoded (36 males and 17 females). The barcodes form one unique cluster. The minimal p-distance between two barcodes in this cluster is 0.00% and the maximal p-distance is 1.67% (mean = 0.18%). The nearest neighboring cluster to the cluster of the barcodes of *N. appendiculata* is the one of the barcodes of *Nephrotoma quadrifaria* (Meigen, 1804). The minimal p-distance between a barcode from the *N. appendiculata* and the *N. quadrifaria* cluster is 7.95%. mPTP splits the cluster of barcodes of *N. appendiculata* into two MOTUs (51 barcodes and 2 barcodes).

Nephrotoma cornicina (Linnaeus, 1758)

19 adult specimens from Germany of *Nephrotoma cornicina* (Linnaeus, 1758) have been barcoded (12 males, 6 females and 1 n/a). The barcodes form one unique cluster. The minimal p-distance between two barcodes in this cluster is 0.00% and the maximal p-distance is 1.67% (mean = 0.34%). The nearest neighboring cluster to the cluster of the barcodes of *N. cornicina* is the one of the barcodes of *Nephrotoma quadrifaria* (Meigen, 1804). The minimal p-distance between a barcode from the *N. aculeata* and the *N. quadrifaria* cluster is 7.27%.

Nephrotoma crocata (Linnaeus, 1758)

Six adult specimens from Germany of *Nephrotoma crocata* (Linnaeus, 1758) have been barcoded (4 males and 2 females). The barcodes form one unique cluster. The minimal p-distance between two barcodes in this cluster is 0.00% and the maximal p-distance is 0.46% (mean = 0.15%). The nearest neighboring cluster to the cluster of the barcodes of *N. crocata* is the one of the barcodes of *Nephrotoma scalaris* (Meigen, 1818). The minimal p-distance between a barcode from the *N. crocata* and the *N. scalaris* cluster is 0.46%. mPTP, BIN, ASAP and TaxCI lump the cluster of barcodes of *N. crocata* together with the cluster of barcodes of *N. scalaris* into one MOTU.

Nephrotoma dorsalis (Fabricius, 1781)

27 adult specimens of *Nephrotoma dorsalis* (Fabricius, 1781) have been barcoded (20 males and 7 females). 24 specimens are from Germany and 2 specimens are from Italy. The barcodes form one unique cluster. The minimal p-distance between two barcodes in this cluster is 0.00% and the maximal p-distance is 0.91% (mean = 0.22%). The nearest neighboring cluster to the cluster of the

barcodes of *N. dorsalis* is the one of the barcodes of *Nephrotoma lunulicornis* (Schummel, 1833). The minimal p-distance between a barcode from the *N. dorsalis* and the *N. lunulicornis* cluster is 5.56%.

Nephrotoma flavescens (Linnaeus, 1758)

39 adult specimens from Germany of *Nephrotoma flavescens* (Linnaeus, 1758) have been barcoded (24 males, 13 females and 2 n/a). The barcodes form one unique cluster. The minimal p-distance between two barcodes in this cluster is 0.00% and the maximal p-distance is 0.76% (mean = 0.31%). The nearest neighboring cluster to the cluster of the barcodes of *N. flavescens* is one of the clusters of barcodes of *Nephrotoma submaculosa* Edwards, 1928. The minimal p-distance between a barcode from the *N. flavescens* and one of the *N. submaculosa* clusters is 0.61%. mPTP and BIN lump the cluster of barcodes of *N. flavescens* with a cluster of 5 barcodes of *N. submaculosa* into one MOTU. ASAP and TaxCI lump the barcodes of *N. flavescens* together with the barcodes of *N. submaculosa* into one MOTU.

Nephrotoma flavipalpis (Meigen, 1830)

Seven adult specimens from Germany of *Nephrotoma flavipalpis* (Meigen, 1830) have been barcoded (6 males and 1 female). The barcodes form one unique cluster. All barcodes in this cluster are identical (no distance). The nearest neighboring cluster to the cluster of the barcodes of *N. flavipalpis* is the one of the barcodes of *Nephrotoma analis* (Schummel, 1833). The minimal p-distance between a barcode from the *N. flavipalpis* and the *N. analis* cluster is 5.48%.

Nephrotoma guestfalica (Westhoff, 1879)

Five adult specimens from Germany of *Nephrotoma guestfalica* (Westhoff, 1879) have been barcoded (3 males and 2 females). The barcodes form one unique cluster. The minimal p-distance between two barcodes in this cluster is 0.00% and the maximal p-distance is 1.82% (mean = 0.73%). The nearest neighboring cluster to the cluster of the barcodes of *N. guestfalica* is the one of the barcodes of *Nephrotoma appendiculata* (Pierre, 1919). The minimal p-distance between a barcode from the *N. guestfalica* and the *N .appendiculata* cluster is 10.26%. mPTP and TaxCI split the cluster of barcodes of *N. guestfalica* into two MOTUs (4 barcodes and 1 barcode).

Nephrotoma lamellata (Riedel, 1910)

One male, adult specimen from Germany of *Nephrotoma lamellata* (Riedel, 1910) has been barcoded (singleton). The nearest neighboring cluster to the barcode of *N. lamellata* is the one of the barcodes of *Nephrotoma analis* (Schummel, 1833). The minimal p-distance between the barcode of *N. lamellata* and a barcode from the *N. analis* cluster is 3.55%.

Nephrotoma lunulicornis (Schummel, 1833)

Two adult specimens from Germany of *Nephrotoma lunulicornis* (Schummel, 1833) have been barcoded (1 male and 1 female). The barcodes form one unique cluster. The barcodes are identical (no distance). The nearest neighboring cluster to the cluster of the barcodes of *N. lunulicornis* is the one of the barcodes of *Nephrotoma dorsalis* (Fabricius, 1781). The minimal p-distance between a barcode from the *N. lunulicornis* and the *N. dorsalis* cluster is 5.56%.

Nephrotoma pratensis (Linnaeus, 1758)

Five adult specimens from Germany of *Nephrotoma pratensis* (Linnaeus, 1758) have been barcoded (3 males and 2 females). The barcodes form one unique cluster. The minimal p-distance between two barcodes in this cluster is 0.00% and the maximal p-distance is 0.30% (mean = 0.12%). The nearest

neighboring clusters to the cluster of the barcodes of *N. pratensis* are the ones of the barcodes of *Nephrotoma crocata* (Linnaeus, 1758) and *Nephrotoma scalaris* (Meigen, 1818). The minimal p-distance between a barcode from the *N. pratensis* and the *N. crocata* or the *N. scalaris* cluster is 6.08%.

Nephrotoma quadrifaria (Meigen, 1804)

33 adult specimens from Germany of *Nephrotoma quadrifaria* (Meigen, 1804) have been barcoded (15 males and 18 females). The barcodes form one unique cluster. The minimal p-distance between two barcodes in this cluster is 0.00% and the maximal p-distance is 0.76% (mean = 0.16%). The nearest neighboring clusters to the cluster of the barcodes of *N. quadrifaria* are the ones of the barcodes of *Nephrotoma aculeata* (Loew, 1871) and *Nephrotoma submaculosa* Edwards, 1928. The minimal p-distance between a barcode from the *N. quadrifaria* and the *N. aculeata* or the *N. submaculosa* cluster is 7.02%.

Nephrotoma quadristriata (Schummel, 1833)

Two male, adult specimens from Germany of *Nephrotoma quadristriata* (Schummel, 1833) have been barcoded. The barcodes form one unique cluster. The p-distance between the barcodes is 1.06%. The nearest neighboring clusters to the cluster of the barcodes of *N. quadristriata* are the ones of the barcodes of *Nephrotoma dorsalis* (Fabricius, 1781) and *Nephrotoma lunulicornis* (Schummel, 1833). The minimal p-distance between a barcode from the *N. quadristriata* and the *N. dorsalis* or the *N. lunulicornis* cluster is 6.08%.

Nephrotoma scalaris (Meigen, 1818)

Nine adult specimens from Germany of *Nephrotoma scalaris* (Meigen, 1818) have been barcoded (8 males and 1 female). The barcodes form one unique cluster. The minimal p-distance between two barcodes in this cluster is 0.00% and the maximal p-distance is 0.30% (mean = 0.10%). The nearest neighboring cluster to the cluster of the barcodes of *N. scalaris* is the one of the barcodes of *Nephrotoma crocata* (Linnaeus, 1758). The minimal p-distance between a barcode from the *N. scalaris* and the *N. crocata* cluster is 0.46%. mPTP, BIN, ASAP and TaxCI lump the cluster of barcodes of *N. scalaris* together with the cluster of barcodes of *N. crocata* into one MOTU.

Nephrotoma scurra (Meigen, 1818)

26 adult specimens of *Nephrotoma scurra* (Meigen, 1818) have been barcoded (22 males and 4 females). 24 specimens are from Germany and 1 specimen is from Italy. The barcodes form one unique cluster. The minimal p-distance between two barcodes in this cluster is 0.00% and the maximal p-distance is 2.89% (mean = 1.16%). The nearest neighboring cluster to the cluster of the barcodes of *N. scurra* is the one of the barcodes of *Nephrotoma dorsalis* (Fabricius, 1781). The minimal p-distance between a barcode from the *N. scurra* and the *N. dorsalis* cluster is 6.81%.

Nephrotoma submaculosa Edwards, 1928

12 adult specimens from Germany of *Nephrotoma submaculosa* Edwards, 1928 have been barcoded (9 males and 3 females). The barcodes form two clusters. One cluster consisting of the barcodes of 5 specimens (4 males and 1 female) neighbors the cluster of barcodes of the specimens of *Nephrotoma flavescens* (Linnaeus, 1758). The other 7 specimens' barcodes (5 males and 2 females) form a cluster neighboring the cluster consisting of the cluster of the 5 specimens' barcodes of *N. submaculosa* and the cluster of the specimens' barcodes of *N. flavescens*. The minimal p-distance between two barcodes of *N. submaculosa* is 0.00% and the maximal p-distance is 1.98% (mean = 1.12%). The minimal p-distance between a barcode of *N. submaculosa* and a barcode of *N. flavescens* is 0.61%.

mPTP and BIN split the barcodes of *N. submaculosa* into two MOTUs (5 and 7 barcodes) and lumps the cluster of 5 barcodes of *N. submaculosa* with the cluster of barcodes of *N. flavescens* into one MOTU. ASAP and TaxCI lump the barcodes of *N. submaculosa* together with the cluster of barcodes of *N. flavescens* into one MOTU.

Nigrotipula nigra (Linnaeus, 1758)

Eight male, adult specimens from Germany of *Nigrotipula nigra* (Linnaeus, 1758) have been barcoded. The barcodes form one unique cluster. The minimal p-distance between two barcodes in this cluster is 0.15% and the maximal p-distance is 1.22% (mean = 0.47%). The nearest neighboring cluster to the cluster of the barcodes of *N. nigra* is the one of the barcodes of *Tipula* (*Pterelachisus*) *varipennis* Meigen, 1818. The minimal p-distance between a barcode from the *N. nigra* and the *T. varipennis* cluster is 10.05%.

Prionocera pubescens Loew, 1844

One female, adult specimen from Germany of *Prionocera pubescens* Loew, 1844 has been barcoded (singleton). The nearest neighboring cluster to the barcode of *P. pubescens* is the one of the barcodes of *Prionocera subserricornis* (Zetterstedt, 1851). The minimal p-distance between the barcode of *P. pubescens* and a barcode from the *P. subserricornis* cluster is 5.02%.

Prionocera subserricornis (Zetterstedt, 1851)

Two adult specimens from Germany of *Prionocera subserricornis* (Zetterstedt, 1851) have been barcoded (1 male and 1 female). The barcodes form one unique cluster. The p-distance between the barcodes is 0.30%. The nearest neighboring cluster to the cluster of the barcodes of *P. subserricornis* is the one of the barcodes of *Prionocera turcica* (Fabricius, 1787). The minimal p-distance between a barcode from the *P. subserricornis* and the *P. turcica* cluster is 1.52%. mPTP, BIN, ASAP and TaxCI lump the cluster of barcodes of *P. subserricornis* together with the cluster of barcodes of *P. turcica* into one MOTU.

Prionocera turcica (Fabricius, 1787)

Two male, adult specimens from Germany of *Prionocera turcica* (Fabricius, 1787) have been barcoded. The barcodes form one unique cluster. The p-distance between the barcodes is 0.46%. The nearest neighboring cluster to the cluster of the barcodes of *P. turcica* is the one of the barcodes of *Prionocera subserricornis* (Zetterstedt, 1851). The minimal p-distance between a barcode from the *P. turcica* and the *P. subserricornis* cluster is 1.52%. mPTP, BIN, ASAP and TaxCI lump the cluster of barcodes of *P. turcica* together with the cluster of barcodes of *P. subserricornis* into one MOTU.

Tanyptera (*Tanyptera*) *atrata* (Linnaeus, 1758)

27 adult specimens from Germany of *Tanyptera* (*Tanyptera*) *atrata* (Linnaeus, 1758) have been barcoded (16 males and 11 females). The barcodes form one unique cluster. The minimal p-distance between two barcodes in this cluster is 0.00% and the maximal p-distance is 0.51% (mean = 0.19%). The nearest neighboring cluster to the cluster of the barcodes of *T. atrata* is the one of the barcodes of *Tanyptera* (*Tanyptera*) *nigricornis* (Meigen, 1818). The minimal p-distance between a barcode from the *T. atrata* and the *T. nigricornis* cluster is 5.17%.

Tanyptera (*Tanyptera*) *nigricornis* (Meigen, 1818)

Seven adult specimens from Germany of *Tanyptera* (*Tanyptera*) *nigricornis* (Meigen, 1818) have been barcoded (6 males and 1 female). The barcodes form one unique cluster. The minimal p-distance

between two barcodes in this cluster is 0.00% and the maximal p-distance is 1.22% (mean = 0.55%). The nearest neighboring cluster to the cluster of the barcodes of *T. nigricornis* is the one of the barcodes of *Tanyptera* (*Tanyptera*) *atrata* (Linnaeus, 1758). The minimal p-distance between a barcode from the *T. nigricornis* and the *T. atrata* cluster is 5.17%.

Tipula (*Lunatipula*) *alpina* Loew, 1873

Three male, adult specimens from Germany of *Tipula* (*Lunatipula*) *alpina* Loew, 1873 have been barcoded. The barcodes form one unique cluster. All barcodes are identical (no distance). The nearest neighboring cluster to the cluster of the barcodes of *T. alpina* is the one of the barcodes of *Tipula* (*Lunatipula*) *fascipennis* Meigen, 1818. The minimal p-distance between a barcode from the *T. alpina* and the *T. fascipennis* cluster is 5.32%.

Tipula (*Lunatipula*) *cava* Riedel, 1913

Six male, adult specimens from Germany of *Tipula* (*Lunatipula*) *cava* Riedel, 1913 have been barcoded. The barcodes form one unique cluster. The minimal p-distance between two barcodes in this cluster is 0.00% and the maximal p-distance is 0.30% (mean = 0.26%). The nearest neighboring cluster to the cluster of the barcodes of *T. cava* is the one of the barcodes of *Tipula* (*Lunatipula*) *alpina* Loew, 1873. The minimal p-distance between a barcode from the *T. cava* and the *T. alpina* cluster is 7.75%.

Tipula (*Savtshenkia*) *confusa* van der Wulp, 1883

14 adult specimens from Germany of *Tipula* (*Savtshenkia*) *confusa* van der Wulp, 1883 have been barcoded (9 males and 5 females).The barcodes form one unique cluster. The minimal p-distance between two barcodes in this cluster is 0.00% and the maximal p-distance is 0.61% (mean = 0.22%). The nearest neighboring cluster to the cluster of the barcodes of *T. confusa* is the one of the barcodes of *Tipula* (*Savtshenkia*) *rufina* Meigen, 1818. The minimal p-distance between a barcode from the *T. confusa* and the *T. rufina* cluster is 6.84%.

Tipula (*Yamatotipula*) *couckei* Tonnoir, 1921

31 adult specimens from Germany of *Tipula* (*Yamatotipula*) *couckei* Tonnoir, 1921 have been barcoded (5 males, 25 females and 1 n/a). The barcodes form one unique cluster. The minimal p-distance between two barcodes in this cluster is 0.00% and the maximal p-distance is 0.30% (mean = 0.04%). The nearest neighboring cluster to the cluster of the barcodes of *T. couckei* is the one of the barcodes of *Tipula* (*Yamatotipula*) *pierrei* Tonnoir, 1921. The minimal p-distance between a barcode from the *T. couckei* and the *T. pierrei* cluster is 5.02%.

Tipula (*Lunatipula*) *fascipennis* Meigen, 1818

42 adult specimens of *Tipula* (*Lunatipula*) *fascipennis* Meigen, 1818 have been barcoded (29 males and 13 females). 38 specimens are from Germany and 4 specimens are from Italy. The barcodes form one unique cluster. The minimal p-distance between two barcodes in this cluster is 0.00% and the maximal p-distance is 0.61% (mean = 0.14%). The nearest neighboring cluster to the cluster of the barcodes of *T. fascipennis* is the one of the barcodes of *Tipula* (*Lunatipula*) *vernalis* Meigen, 1804. The minimal p-distance between a barcode from the *T. fascipennis* and the *T. vernalis* cluster is 4.72%.

Tipula (*Dendrotipula*) *flavolineata* Meigen, 1804

10 adult specimens from Germany of *Tipula* (*Dendrotipula*) *flavolineata* Meigen, 1804 have been barcoded (7 males and 3 females).The barcodes form one unique cluster. The minimal p-distance between two barcodes in this cluster is 0.00% and the maximal p-distance is 0.61% (mean = 0.25%). The nearest neighboring cluster to the cluster of the barcodes of *T. flavolineata* is the one of the barcodes of *Tipula* (*Savtshenkia*) *pagana* Meigen, 1818. The minimal p-distance between a barcode from the *T. flavolineata* and the *T. pagana* cluster is 9.57%.

Tipula (*Acutipula*) *fulvipennis* De Geer, 1776

Two male, adult specimens from Germany of *Tipula* (*Acutipula*) *fulvipennis* De Geer, 1776 have been barcoded. The barcodes form one unique cluster. The p-distance between the barcodes is 0.30%. The nearest neighboring cluster to the cluster of the barcodes of *T. fulvipennis* is the one of the barcodes of *Tipula* (*Tipula*) *oleracea* Linnaeus, 1758. The minimal p-distance between a barcode from the *T. fulvipennis* and the *T. oleracea* cluster is 9.12%.

Tipula (*Lunatipula*) *helvola* Loew, 1873

16 adult specimens of *Tipula* (*Lunatipula*) *helvola* Loew, 1873 have been barcoded (8 males and 8 females). 15 specimens are from Germany and specimen is from the Netherlands. The barcodes form one unique cluster. The minimal p-distance between two barcodes in this cluster is 0.00% and the maximal p-distance is 0.30% (mean = 0.20%). The nearest neighboring cluster to the cluster of the barcodes of *T. helvola* is the one of the barcodes of *Tipula* (*Lunatipula*) *vernalis* Meigen, 1804. The minimal p-distance between a barcode from the *T. helvola* and the *T. vernalis* cluster is 5.49%.

Tipula (*Vestiplex*) *hortorum* Linnaeus, 1758

Nine adult specimens from Germany of *Tipula* (*Vestiplex*) *hortorum* Linnaeus, 1758 have been barcoded (7 males and 2 females). The barcodes form one cluster together with the barcodes of *Tipula* (*Vestiplex*) *nubeculosa* Meigen, 1804. The minimal p-distance between two barcodes of *T. hortorum* in this cluster is 0.00% and the maximal p-distance is 1.98% (mean = 1.16%). The minimal p-distance between a barcode from *T. hortorum* and *T. nubeculosa* in this cluster is 0.15%. mPTP, BIN, ASAP and TaxCI lump the barcodes of *T. hortorum* together with the barcodes of *T. nubeculosa* into one MOTU.

Tipula (*Pterelachisus*) *irrorata* Macquart, 1826

Six adult specimens from Germany of *Tipula* (*Pterelachisus*) *irrorata* Macquart, 1826 have been barcoded (4 males and 2 females). The barcodes form one unique cluster. The minimal p-distance between two barcodes in this cluster is 0.00% and the maximal p-distance is 0.30% (mean = 0.15%). The nearest neighboring cluster to the cluster of the barcodes of *T. irrorata* is the one of the barcodes of *Tipula* (*Pterelachisus*) *varipennis* Meigen, 1818. The minimal p-distance between a barcode from the *T. irrorata* and the *T. varipennis* cluster is 6.93%.

Tipula (*Lunatipula*) *laetabilis* Zetterstedt, 1838

Two male, adult specimens from Germany of *Tipula* (*Lunatipula*) *laetabilis* Zetterstedt, 1838 have been barcoded. The barcodes form one unique cluster. The p-distance between the two barcodes is 0.30%. The nearest neighboring cluster to the cluster of the barcodes of *T. laetabilis* is the one of the barcodes of *Tipula* (*Lunatipula*) *magnicauda* Strobl, 1895. The minimal p-distance between a barcode from the *T. laetabilis* and the *T. magnicauda* cluster is 9.28%.

Tipula (*Yamatotipula*) *lateralis* Meigen, 1804

Nine adult specimens from Germany of *Tipula* (*Yamatotipula*) *lateralis* Meigen, 1804 have been barcoded (8 males and 1 female). The barcodes form one unique cluster. The minimal p-distance between two barcodes in this cluster is 0.00% and the maximal p-distance is 0.76% (mean = 0.35%). The nearest neighboring cluster to the cluster of the barcodes of *T. lateralis* is the one of the barcodes of *Tipula* (*Yamatotipula*) *montium* Egger, 1863. The minimal p-distance between a barcode from the *T. lateralis* and the *T. montium* cluster is 4.41%.

Tipula (*Lunatipula*) *limitata* Schummel, 1833

Three adult specimens from Germany of *Tipula* (*Lunatipula*) *limitata* Schummel, 1833 have been barcoded (2 males and 1 female).The barcodes form one unique cluster. The minimal p-distance between two barcodes in this cluster is 0.15% and the maximal p-distance is 0.46% (mean = 0.30%). The nearest neighboring cluster to the cluster of the barcodes of *T. limitata* is the one of the barcodes of *Tipula* (*Lunatipula*) *alpina* Loew, 1873. The minimal p-distance between a barcode from the *T. limitata* and the *T. alpina* cluster is 6.85%.

Tipula (*Lunatipula*) *livida* van der Wulp, 1859

Seven adult specimens from Germany of *Tipula* (*Lunatipula*) *livida* van der Wulp, 1859 have been barcoded (5 males and 2 females).The barcodes form one unique cluster. The minimal p-distance between two barcodes in this cluster is 0.00% and the maximal p-distance is 0.15% (mean = 0.04%). The nearest neighboring cluster to the cluster of the barcodes of *T. livida* is the one of the barcodes of *Tipula* (*Lunatipula*) *selene* Meigen, 1830. The minimal p-distance between a barcode from the *T. livida* and the *T. selene* cluster is 7.29%.

Tipula (*Acutipula*) *luna* Westhoff, 1879

18 adult specimens from Germany of *Tipula* (*Acutipula*) *luna* Westhoff, 1879 have been barcoded (15 males and 3 females). The barcodes form one unique cluster. The minimal p-distance between two barcodes in this cluster is 0.00% and the maximal p-distance is 0.76% (mean = 0.22%). The nearest neighboring cluster to the cluster of the barcodes of *T. luna* is the one of the barcodes of *Tipula* (*Acutipula*) *maxima* Poda, 1761. The minimal p-distance between a barcode from the *T. luna* and the *T. maxima* cluster is 10.18%.

Tipula (*Lunatipula*) *lunata* Linnaeus, 1758

39 adult specimens from Germany of *Tipula* (*Lunatipula*) *lunata* Linnaeus, 1758 have been barcoded (35 males and 4 females). The barcodes form one unique cluster. The minimal p-distance between two barcodes in this cluster is 0.00% and the maximal p-distance is 2.58% (mean = 0.60%). The nearest neighboring cluster to the cluster of the barcodes of *T. lunata* is the one of the barcodes of *Tipula* (*Lunatipula*) *selene* Meigen, 1830. The minimal p-distance between a barcode from the *T. lunata* and the *T. selene* cluster is 5.62%. mPTP and BIN split the cluster of barcodes of *T. lunata* into two MOTUs (38 barcodes and 1 barcode).

Tipula (*Lunatipula*) *magnicauda* Strobl, 1895

14 adult specimens from Germany of *Tipula* (*Lunatipula*) *magnicauda* Strobl, 1895 have been barcoded (13 males and 1 female). The barcodes form one unique cluster. All barcodes are identical (no distance). The nearest neighboring cluster to the cluster of the barcodes of *T. magnicauda* is the one of the barcodes of *Tipula* (*Lunatipula*) *selene* Meigen, 1830. The minimal p-distance between a barcode from the *T. magnicauda* and the *T. selene* cluster is 6.38%.

Tipula (*Acutipula*) *maxima* Poda, 1761

12 adult specimens from Germany of *Tipula* (*Acutipula*) *maxima* Poda, 1761 have been barcoded (11 males and 1 female). The barcodes form one unique cluster. The minimal p-distance between two barcodes in this cluster is 0.00% and the maximal p-distance is 0.61% (mean = 0.13%). The nearest neighboring cluster to the cluster of the barcodes of *T. maxima* is the one of the barcodes of *Tipula* (*Acutipula*) *luna* Westhoff, 1879. The minimal p-distance between a barcode from the *T. maxima* and the *T. luna* cluster is 10.18%.

Tipula (*Lunatipula*) *mellea* Schummel, 1833

One female, adult specimen from Germany of *Tipula* (*Lunatipula*) *mellea* Schummel, 1833 has been barcoded (singleton). The nearest neighboring cluster to the barcode of *T. mellea* is the one of the barcodes of *Tipula* (*Lunatipula*) *fascipennis* Meigen, 1818. The minimal p-distance between the barcode of *T. mellea* and a barcode from the *T. fascipennis* cluster is 5.62%.

Tipula (*Yamatotipula*) *montium* Egger, 1863

12 specimens from Germany of *Tipula* (*Yamatotipula*) *montium* Egger, 1863 have been barcoded (6 males, 4 females and 2 n/a). 11 specimens are adults and 1 specimen is a larva. The barcodes form one unique cluster. The minimal p-distance between two barcodes in this cluster is 0.00% and the maximal p-distance is 0.61% (mean = 0.15%). The nearest neighboring cluster to the cluster of the barcodes of *T. montium* is the one of the barcodes of *Tipula* (*Yamatotipula*) *lateralis* Meigen, 1804. The minimal p-distance between a barcode from the *T. montium* and the *T. lateralis* cluster is 4.41%.

Tipula (*Odonatisca*) *nodicornis* Meigen, 1818

One female, adult specimen from Germany of *Tipula* (*Odonatisca*) *nodicornis* Meigen, 1818 has been barcoded (singleton). The nearest neighboring barcode to the barcode of *T. nodicornis* is the one of *Tipula* (*Pterelachisus*) *trifascingulata* Theowald, 1980. The p-distance between the barcode of *T. nodicornis* and the barcode of *T. trifascingulata* is 12.92%.

Tipula (*Vestiplex*) *nubeculosa* Meigen, 1804

Six adult specimens from Germany of *Tipula* (*Vestiplex*) *nubeculosa* Meigen, 1804 have been barcoded (5 males and 1 female). The barcodes form a cluster together with 3 barcodes of specimens (2 males and 1 female) of *Tipula* (*Vestiplex*) *hortorum* Linnaeus, 1758. The minimal p-distance between two barcodes of *T. nubeculosa* is 0.00% and the maximal p-distance is 1.06% (mean = 0.56%). The minimal p-distance between a barcode from *T. nubeculosa* and *T. hortorum* in this cluster is 0.15%. mPTP, BIN, ASAP and TaxCI lump the barcodes of *T. nubeculosa* together with the barcodes of *T. hortorum* into one MOTU.

Tipula (*Tipula*) *oleracea* Linnaeus, 1758

16 adult specimens from Germany of *Tipula* (*Tipula*) *oleracea* Linnaeus, 1758 have been barcoded (13 males and 3 females). The barcodes form one unique cluster. The minimal p-distance between two barcodes in this cluster is 0.00% and the maximal p-distance is 0.91% (mean = 0.36%). The nearest neighboring barcode to the cluster of the barcodes of *T. oleracea* is the one of *Tipula* (*Tipula*) *subcunctans* Alexander, 1921. The minimal p-distance between a barcode from the *T. oleracea* cluster and the *T. subcunctans* barcode is 4.41%.

Tipula (*Pterelachisus*) *pabulina* Meigen, 1818

10 male, adult specimens from Germany of *Tipula* (*Pterelachisus*) *pabulina* Meigen, 1818 have been barcoded. The barcodes form one unique cluster. The minimal p-distance between two barcodes in this cluster is 0.00% and the maximal p-distance is 1.52% (mean = 0.61%). The nearest neighboring cluster to the cluster of the barcodes of *T. pabulina* is the one of the barcodes of *Tipula* (*Pterelachisus*) *truncorum* Meigen, 1830. The minimal p-distance between a barcode from the *T. pabulina* and the *T. truncorum* cluster is 8.16%.

Tipula (*Savtshenkia*) *pagana* Meigen, 1818

Six adult specimens from Germany of *Tipula* (*Savtshenkia*) *pagana* Meigen, 1818 have been barcoded (5 males and 1 female). The barcodes form one unique cluster. The minimal p-distance between two barcodes in this cluster is 0.00% and the maximal p-distance is 5.78% (mean = 3.54%). The nearest neighboring cluster to the cluster of the barcodes of *T. pabulina* is the one of the barcodes of *Tipula* (*Savtshenkia*) *confusa* van der Wulp, 1883. The minimal p-distance between a barcode from the *T. pagana* and the *T. confusa* cluster is 7.90%. mPTP, BIN, ASAP and TaxCI split the cluster of barcodes of *T. pagana* into two MOTUs (3 and 3 barcodes).

Tipula (*Tipula*) *paludosa* Meigen, 1830

30 adult specimens of *Tipula* (*Tipula*) *paludosa* Meigen, 1830 have been barcoded (29 males and 1 female). 29 specimens are from Germany and 1 specimen is from the Netherlands. The barcodes form two clusters. One cluster consisting of the barcodes of 27 specimens (all males; 28 from Germany, 1 from the Netherlands) neighbors the barcode of the specimen of *Tipula* (*Tipula*) *subcunctans* Alexander, 1921. The other 3 specimens' barcodes (2 males, 1 female; all from Germany) form a cluster neighboring the cluster consisting of the cluster of the 27 specimens' barcodes of *T. paludosa* and the specimens' barcode of *T. subcunctans*. The minimal p-distance between two barcodes of *T. paludosa* is 0.00% and the maximal p-distance is 3.96% (mean = 0.87%). The minimal p-distance between a barcode of *T. paludosa* and a barcode of *T. subcunctans* is 2.65%. mPTP, BIN and TaxCI split the barcodes of *T. paludosa* into two MOTUs (27 and 3 barcodes). ASAP lumps the barcodes of *T. paludosa* together with the barcode of *T. subcunctans* into one MOTU.

Tipula (*Lunatipula*) *peliostigma* Schummel, 1833

11 male, adult specimens from Germany of *Tipula* (*Lunatipula*) *peliostigma* Schummel, 1833 have been barcoded. The barcodes form one unique cluster. The minimal p-distance between two barcodes in this cluster is 0.00% and the maximal p-distance is 0.46% (mean = 0.13%). The nearest neighboring cluster to the cluster of the barcodes of *T. peliostigma* is the one of the barcodes of *Tipula* (*Lunatipula*) *selene* Meigen, 1830. The minimal p-distance between a barcode from the *T. peliostigma* and the *T. selene* cluster is 4.86%.

Tipula (*Yamatotipula*) *pierrei* Tonnoir, 1921

Three adult specimens from Germany of *Tipula* (*Yamatotipula*) *pierrei* Tonnoir, 1921 have been barcoded (2 males and 1 female).The barcodes form one unique cluster. The minimal p-distance between two barcodes in this cluster is 0.46% and the maximal p-distance is 1.07% (mean = 0.71%). The nearest neighboring cluster to the cluster of the barcodes of *T. pierrei* is the one of the barcodes of *Tipula* (*Yamatotipula*) *couckei* Tonnoir, 1921. The minimal p-distance between a barcode from the *T. pierrei* and the *T. couckei* cluster is 5.02%.

Tipula (*Yamatotipula*) *pruinosa* Wiedemann, 1817

One female, adult specimen from Germany of *Tipula* (*Yamatotipula*) *pruinosa* Wiedemann, 1817 has been barcoded (singleton). The nearest neighboring cluster to the barcode of *T. pruinosa* is the one of the barcodes of *Tipula* (*Lunatipula*) *vernalis* Meigen, 1804. The minimal p-distance between the barcode of *T. pruinosa* and a barcode from the *T. vernalis* cluster is 9.27%.

Tipula (*Pterelachisus*) *pseudovariipennis* Czizek, 1912

11 adult specimens from Germany of *Tipula* (*Pterelachisus*) *pseudovariipennis* Czizek, 1912 have been barcoded (9 males and 2 females).The barcodes form one unique cluster. The minimal p-distance between two barcodes in this cluster is 0.00% and the maximal p-distance is 1.82% (mean = 0.91%). The nearest neighboring cluster to the cluster of the barcodes of *T. pseudovariipennis* is one of the clusters of barcodes of *Tipula* (*Pterelachisus*) *varipennis* Meigen, 1818. The minimal p-distance between a barcode from the *T. pseudovariipennis* and one of the *T. varipennis* clusters is 1.02%. ASAP lumps the cluster of barcodes of *T. pseudovariipennis,* together with the cluster of barcodes of *T. varipennis* and the cluster of barcodes of *Tipula* (*Pterelachisus*) *submarmorata* Schummel, 1833 into one MOTU. TaxCI lumps the cluster of barcodes of *T. pseudovariipennis* together with the cluster of barcodes of *T. varipennis* into one MOTU.

Tipula (*Savtshenkia*) *rufina* Meigen, 1818

Four adult specimens from Germany of *Tipula* (*Savtshenkia*) *rufina* Meigen, 1818 have been barcoded (1 male and 3 females). The barcodes form one unique cluster. The minimal p-distance between two barcodes in this cluster is 0.15% and the maximal p-distance is 0.91% (mean = 0.58%). The nearest neighboring barcode to the cluster of the barcodes of *T. rufina* is the one of *Tipula* (*Savtshenkia*) *staegeri* Nielsen, 1922. The minimal p-distance between a barcode from the *T. rufina* cluster and the *T. staegeri* barcode is 6.69%.

Tipula (*Vestiplex*) *scripta* Meigen, 1830

Six male, adult specimens from Germany of *Tipula* (*Vestiplex*) *scripta* Meigen, 1830 have been barcoded. The barcodes form one unique cluster. The minimal p-distance between two barcodes in this cluster is 0.00% and the maximal p-distance is 0.46% (mean = 0.23%). The nearest neighboring cluster to the cluster of the barcodes of *T. scripta* is the one of the barcodes of *Tipula* (*Lunatipula*) *vernalis* Meigen, 1804. The minimal p-distance between a barcode from the *T. scripta* and the *T. vernalis* cluster is 8.84%.

Tipula (*Lunatipula*) *selene* Meigen, 1830

One male, adult specimen from Germany of *Tipula* (*Lunatipula*) *selene* Meigen, 1830 has been barcoded (singleton). The nearest neighboring cluster to the barcode of *T. selene* is the one of the barcodes of *Tipula* (*Lunatipula*) *peliostigma* Schummel, 1833. The minimal p-distance between the barcode of *T. selene* and a barcode from the *T. peliostigma* cluster is 4.86%.

Tipula (*Pterelachisus*) sp. aff. *trifascingulata, irrorata, submarmorata, varipennis, pseudovariipennis*

One female, adult specimen from Germany of *Tipula* (*Pterelachisus*) sp. has been barcoded (singleton). The nearest neighboring barcode to the barcode of *T.* sp. is the one of *Tipula* (*Pterelachisus*) *trifascingulata* Theowald, 1980. The p-distance between the barcode of *T.* sp. and the barcode of *T. trifascingulata* is 5.93%. mPTP lumps the barcode of *T.* sp. together with the barcode of *T. trifascingulata* into one MOTU.

Tipula (*Savtshenkia*) *staegeri* Nielsen, 1922

One male, adult specimen from Germany of *Tipula* (*Savtshenkia*) *staegeri* Nielsen, 1922 has been barcoded (singleton). The nearest neighboring barcode to the barcode of *T. staegeri* is the one of *Tipula* (*Savtshenkia*) *subvafra* Lackschewitz, 1936. The p-distance between the barcode of *T. staegeri* and the barcode of *T. subvafra* is 6.23%. mPTP lumps the barcode of *T. staegeri* together with the barcode of *T. subvafra* into one MOTU.

Tipula (*Mediotipula*) *stigmatella* Schummel, 1833

Two male, adult specimens from Germany of *Tipula* (*Mediotipula*) *stigmatella* Schummel, 1833 have been barcoded. The barcodes form one unique cluster. The p-distance between the two barcodes is 0.15%. The nearest neighboring cluster to the cluster of the barcodes of *T. stigmatella* is the one of the barcodes of *Tanyptera* (*Tanyptera*) *atrata* (Linnaeus, 1758). The minimal p-distance between a barcode from the *T. stigmatella* and the *T. atrata* cluster is 8.63%.

Tipula (*Tipula*) *subcunctans* Alexander, 1921

One male, adult specimen from Germany of *Tipula* (*Tipula*) *subcunctans* Alexander, 1921 has been barcoded (singleton). The nearest neighboring cluster to the barcode of *T. subcunctans* is the one of the barcodes of *Tipula* (*Tipula*) *paludosa* Meigen, 1830. The minimal p-distance between the barcode of *T. subcunctans* and a barcode from one of the *T. paludosa* clusters is 2.65%. ASAP lumps the barcode of *T. subcunctans* together with the barcodes of *T. paludosa* into one MOTU.

Tipula (*Pterelachisus*) *submarmorata* Schummel, 1833

Six male, adult specimens from Germany of *Tipula* (*Pterelachisus*) *submarmorata* Schummel, 1833 have been barcoded. The barcodes form one unique cluster. All barcodes are identical (no distance). The nearest neighboring cluster to the cluster of the barcodes of *T. submarmorata* is one of the clusters of barcodes of *Tipula* (*Pterelachisus*) *varipennis* Meigen, 1818. The minimal p-distance between a barcode from the *T. submarmorata* and one of the *T. varipennis* clusters is 2.89%. ASAP lumps the cluster of barcodes of *T. submarmorata* together with the cluster of barcodes of *T. varipennis* and the cluster of barcodes of *Tipula* (*Pterelachisus*) *pseudovariipennis* Czizek, 1912 into one MOTU.

Tipula (*Savtshenkia*) *subvafra* Lackschewitz, 1936

One male, adult specimen from Germany of *Tipula* (*Savtshenkia*) *subvafra* Lackschewitz, 1936 has been barcoded (singleton). The nearest neighboring barcode to the barcode of *T. subvafra* is the one of *Tipula* (*Savtshenkia*) *staegeri* Nielsen, 1922. The p-distance between the barcode of *T. subvafra* and the barcode of *T. staegeri* is 6.23%. mPTP lumps the barcode of *T. subvafra* together with the barcode of *T. staegeri* into one MOTU.

Tipula (*Pterelachisus*) *trifascingulata* Theowald, 1980

One male, adult specimen from Germany of *Tipula* (*Pterelachisus*) *trifascingulata* Theowald, 1980 has been barcoded (singleton). The nearest neighboring barcode to the barcode of *T. trifascingulata* is the one of *Tipula* sp. The p-distance between the barcode of *T. trifascingulata* and the barcode of *T.* sp. is 5.93%. mPTP lumps the barcode of *T. trifascingulata* together with the barcode of *T.* sp. into one MOTU.

Tipula (*Pterelachisus*) *truncorum* Meigen, 1830

Two adult specimens from Germany of *Tipula* (*Pterelachisus*) *truncorum* Meigen, 1830 have been barcoded (1 male and 1 female).The barcodes form one unique cluster. The p-distance between the barcodes is 1.22%. The nearest neighboring cluster to the cluster of the barcodes of *T. truncorum* is the one of the barcodes of *Tipula* (*Pterelachisus*) *pabulina* Meigen, 1818. The minimal p-distance between a barcode from the *T. truncorum* and the *T. pabulina* cluster is 8.16%.

Tipula (*Beringotipula*) *unca* Wiedemann, 1817

Six adult specimens from Germany of *Tipula* (*Beringotipula*) *unca* Wiedemann, 1817 have been barcoded (4 males and 2 females). The barcodes form one unique cluster. The minimal p-distance between two barcodes in this cluster is 0.00% and the maximal p-distance is 0.46% (mean = 0.20%). The nearest neighboring barcode to the cluster of the barcodes of *T. unca* is the one of *Tipula* (*Tipula*) *oleracea* Linnaeus, 1758. The minimal p-distance between a barcode from the *T. unca* cluster and the *T. oleracea* barcode is 10.94%.

Tipula (*Schummelia*) *variicornis* Schummel, 1833

15 adult specimens from Germany of *Tipula* (*Schummelia*) *variicornis* Schummel, 1833 have been barcoded (11 males and 4 females). The barcodes form one unique cluster. All barcodes are identical (no distance). The nearest neighboring barcode to the cluster of the barcodes of *T. variicornis* is the one of *Ctenophora* (*Ctenophora*) *pectinicornis* (Linnaeus, 1758). The minimal p-distance between a barcode from the *T. variicornis* cluster and the *C. pectinicornis* barcode is 9.73%.

Tipula (*Pterelachisus*) *varipennis* Meigen, 1818

14 male, adult specimens from Germany of *Tipula* (*Pterelachisus*) *varipennis* Meigen, 1818 have been barcoded. The barcodes form one unique cluster. The minimal p-distance between two barcodes of *T. varipennis* is 0.00% and the maximal p-distance is 1.08% (mean = 0.54%). The minimal p-distance between a barcode of *T. varipennis* and a barcode of *T. pseudovariipennis* is 1.02%. ASAP lumps the cluster of barcodes of *T. varipennis,* together with the cluster of barcodes of *T. pseudovariipennis* and the cluster of barcodes of *Tipula* (*Pterelachisus*) *submarmorata* Schummel, 1833 into one MOTU. TaxCI lumps the cluster of barcodes of *T. varipennis* together with the cluster of barcodes of *T. pseudovariipennis* into one MOTU.

Tipula (*Lunatipula*) *vernalis* Meigen, 1804

43 adult specimens of *Tipula* (*Lunatipula*) *vernalis* Meigen, 1804 have been barcoded (27 males and 16 females). 41 specimens are from Germany and 2 specimens are from Italy. The barcodes form one unique cluster. The minimal p-distance between two barcodes in this cluster is 0.00% and the maximal p-distance is 2.89% (mean = 0.51%). The nearest neighboring cluster to the cluster of the barcodes of *T. vernalis* is the one of the barcodes of *Tipula* (*Lunatipula*) *fascipennis* Meigen, 1818. The minimal p-distance between a barcode from the *T. vernalis* and the *T. fascipennis* cluster is 4.72%.

Tipula (*Lunatipula*) *verrucosa* Pierre, 1919

Four male, adult specimens from Germany of *Tipula* (*Lunatipula*) *verrucosa* Pierre, 1919 have been barcoded. The barcodes form one unique cluster. The minimal p-distance between two barcodes in this cluster is 0.00% and the maximal p-distance is 0.76% (mean = 0.51%). The nearest neighboring cluster to the cluster of the barcodes of *T. verrucosa* is the one of the barcodes of *Tipula* (*Lunatipula*)

alpina Loew, 1873. The minimal p-distance between a barcode from the *T. verrucosa* and the *T. alpina* cluster is 7.14%.

Tipula (*Acutipula*) *vittata* Meigen, 1804

10 adult specimens from Germany of *Tipula* (*Acutipula*) *vittata* Meigen, 1804 have been barcoded (8 males and 2 females). The barcodes form one unique cluster. The minimal p-distance between two barcodes in this cluster is 0.00% and the maximal p-distance is 1.06% (mean = 0.60%). The nearest neighboring cluster to the cluster of the barcodes of *T. vittata* is the one of the barcodes of *Tipula* (*Acutipula*) *luna* Westhoff, 1879. The minimal p-distance between a barcode from the *T. vittata* and the *T. luna* cluster is 11.15%.
